# Dapivirine vaginal ring for HIV prevention: modelling health outcomes, drug resistance and cost‐effectiveness

**DOI:** 10.1002/jia2.25282

**Published:** 2019-05-10

**Authors:** Robert Glaubius, Yajun Ding, Kerri J Penrose, Greg Hood, Erik Engquist, John W Mellors, Urvi M Parikh, Ume L Abbas

**Affiliations:** ^1^ Departments of Quantitative Health Sciences and Infectious Disease Cleveland Clinic Cleveland OH USA; ^2^ Department of Medicine Section of Infectious Diseases and Department of Molecular Virology and Microbiology Baylor College of Medicine Houston TX USA; ^3^ Division of Infectious Diseases School of Medicine University of Pittsburgh Pittsburgh PA USA; ^4^ Pittsburgh Supercomputing Center Carnegie Mellon University Pittsburgh PA USA; ^5^ Center for Research Computing Rice University Houston TX USA

**Keywords:** HIV prevention, pre‐exposure prophylaxis/Preexposure prophylaxis/PrEP, dapivirine/DPV, vaginal ring, cost‐effectiveness, drug resistance, mathematical model

## Abstract

**Introduction:**

A vaginal ring containing dapivirine is effective for HIV prevention as pre‐exposure prophylaxis (PrEP). We evaluated the potential epidemiological impact and cost‐effectiveness of dapivirine vaginal ring PrEP among 22‐ to 45‐year‐old women in KwaZulu‐Natal, South Africa.

**Methods:**

Using mathematical modelling, we studied dapivirine vaginal ring PrEP implementation, either unprioritized, or prioritized based on HIV incidence (≥3% per year), age (22 to 29 years) or female sex worker status, alongside the implementation of voluntary medical male circumcision and antiretroviral therapy scaled‐up to UNAIDS Fast‐Track targets. Outcomes over the intervention (2019 to 2030) and lifetime horizons included cumulative HIV infections, life‐years lived, costs and cost‐effectiveness. We assessed the incremental cost‐effectiveness ratios against the revealed willingness to pay ($500) and the standard (2017 per capita gross domestic product; $6161) cost‐effectiveness thresholds for South Africa.

**Results:**

Compared to a reference scenario without PrEP, implementation of dapivirine vaginal ring PrEP, assuming 56% effectiveness and covering 50% of 22 to 29‐year‐old or high‐incidence women, prevented 10% or 11% of infections by 2030 respectively. Equivalent, unprioritized coverage (30%) prevented fewer infections (7%), whereas 50% coverage of female sex workers had the least impact (4%). Drug resistance attributable to PrEP was modest (2% to 4% of people living with drug‐resistant HIV). Over the lifetime horizon, dapivirine PrEP implementation among female sex workers was cost‐saving, whereas incidence‐based PrEP cost $1898 per life‐year gained, relative to PrEP among female sex workers and $989 versus the reference scenario. In a scenario of 37% PrEP effectiveness, PrEP had less impact, but prioritization to female sex workers remained cost‐saving. In uncertainty analysis, female sex worker PrEP was consistently cost‐saving; and over the lifetime horizon, PrEP cost less than $6161 per life‐year gained in over 99% of simulations, whereas incidence‐ and age‐based PrEP cost below $500 per life‐year gained in 61% and 49% of simulations respectively. PrEP adherence and efficacy, and the effectiveness of antiretroviral therapy for HIV prevention, were the principal drivers of uncertainty in the cost‐effectiveness of PrEP.

**Conclusions:**

Dapivirine vaginal ring PrEP would be cost‐saving in KwaZulu‐Natal if prioritized to female sex workers. PrEP's impact on HIV prevention would be increased, with potential affordability, if prioritized to women by age or incidence.

## 1 Introduction

Pre‐exposure prophylaxis (PrEP), the use of antiretrovirals by HIV‐negative individuals to block HIV acquisition, is promising for HIV prevention. Oral PrEP is protective across populations, including men who have sex with men, people who inject drugs, heterosexual men and women and serodiscordant couples 1. Thus, oral PrEP is now recommended for people at substantial risk of HIV (incidence ≥ 3%) 2; available as a fixed‐dose combination of tenofovir disoproxil fumarate and emtricitabine (TDF/FTC) 3, and facilitated by an implementation‐support tool 4. However, daily oral TDF/FTC PrEP was ineffective in two clinical trials among African women (Fem‐PrEP and VOICE) 1, raising concerns about adherence in this population.

Antiretroviral drugs delivered via long‐acting injections or vaginal rings are anticipated to improve PrEP effectiveness by simplifying adherence. ASPIRE and the Ring Study demonstrated the partial effectiveness of a monthly vaginal ring (VR) containing the non‐nucleoside reverse transcriptase inhibitor (NNRTI) dapivirine (DPV) for PrEP among 22‐ to 45‐year‐old women 5, 6; this finding was confirmed by the HOPE trial, an ongoing open‐label extension of ASPIRE 7. However, DPV‐VR's potential long‐term impact on the HIV epidemic remains uncertain. Many countries have adopted the Joint United Nations Programme on HIV/AIDS Fast‐Track approach to ending the AIDS epidemic by 2030 8, including South Africa 9, at substantial anticipated expense 10, yet the impact and cost‐effectiveness of DPV‐VR augmenting the Fast‐Track response remain unclear. Furthermore, the potential drug resistance consequences from DPV‐VR implementation are undefined. Clinical trials 5, 6 did not show any significant selection of majority or minority DPV‐resistant virus in blood, though selection in the genital tract has not been excluded 11. Finally, NNRTI resistance is increasing throughout sub‐Saharan Africa 12, and our *in vitro* work suggests that DPV cross‐resistance is common after first‐line antiretroviral treatment (ART) failure in South Africa 13. Yet, it remains unknown if potential selection of DPV resistance could lead to its spread, and whether circulating drug resistance could limit DPV‐VR's efficacy.

To address these questions, we employed a mathematical model of the HIV epidemic in the hardest‐hit province of South Africa, KwaZulu‐Natal 14, to quantify the population‐level health outcomes, drug resistance consequences and cost‐effectiveness of DPV‐VR PrEP implementation.

## 2 Methods

We extended a mathematical model of the HIV epidemic in KwaZulu‐Natal, with detailed modelling of DPV‐VR PrEP. The model represents the dynamics of HIV transmission, disease progression and drug resistance; is calibrated to longitudinal, age‐ and sex‐stratified data on HIV prevalence and aggregate HIV incidence estimates from the Africa Centre's Demographic Surveillance Site; and supports the implementation of HIV interventions including condom use, voluntary medical male circumcision (VMMC), ART and PrEP. Complete model specification has been reported in the Supplementary Material, and elsewhere examining long‐acting injectable PrEP 15, 16. Model structure, assumptions and analytic design relevant to this study are highlighted below.

### 2.1 Model structure

The model's heterosexual population is stratified by gender, age (15 to 54 years), sexual behaviour, infection status, disease progression, intervention status including first‐ and second‐line ART, VMMC and PrEP, and HIV drug susceptibility.

#### 2.1.1 HIV drug resistance

The model characterizes HIV‐positive individuals by ARV use (not on ARVs, on PrEP or on ART), HIV drug susceptibility (drug‐sensitive or drug‐resistant), type of drug resistance (transmitted or acquired) and virus population dynamics of drug‐resistant HIV (majority or minority). Drug‐resistant virus is either acquired from selection pressure from PrEP or ART, or transmitted from a donor with drug‐resistant HIV. Drug‐resistant HIV may revert to drug‐sensitive wild‐type off of ARVs or in a new host, but archived resistance may re‐emerge with subsequent ARV exposure. For parsimony, we focus on the presence or absence of resistance to the NNRTIs used for first‐line ART, resistance to DPV, or cross‐resistance between the two, but do not characterize specific resistance‐associated mutations. The estimates related to dapivirine cross‐resistance (Table 1) are informed by our laboratory study of HIV isolates from patients failing first‐line ART in South Africa 13. We modelled the dynamics of HIV drug resistance in both blood and genital bodily compartments 17, and assumed that DPV‐VR could select for drug resistance in the female genital tract 18 but not in blood due to low systemic DPV concentrations 19, whereas ART promoted resistance in both compartments. Individuals with genital tract drug resistance could transmit drug‐resistant HIV to their HIV‐negative sexual partners 20, whereas systemic drug‐resistant infection reduced the efficacy of ART upon treatment.

**Table 1:** Key intervention‐related model parameters

Input	Base case	LHS range^a^	Reference
VMMC			
Male circumcision prevalence at Jan. 1, 2021, %	80	60 to 85	14, 26
VMMC effectiveness against male HIV acquisition, %	60	Not varied	23
ART			
Time universal treatment eligibility begins, year	Sep 1, 2016	Not varied	9
ART coverage by Jan 1, 2021, %	81	58 to 84	9, 27
ART coverage by Jan 1, 2031, %	90	72 to 96	27
ART effectiveness against HIV transmission while suppressed, %	96	73 to 99	24
Decrease in ART virologic failure due to adherence support, %	80	0 to 90	8
DPV cross‐resistance prevalence among persons with acquired resistance to first‐line ART, %	80	70 to 100	13
PrEP			
Time PrEP implementation begins, year	Jan 1, 2019	Not varied	Assumed
Time to reach target PrEP coverage, years	4	2 to 6	Assumed
PrEP coverage (as level of HIV‐negative adults aged 15 to 54), %	2.5 to 10	2.5 to 10	Assumed
PrEP coverage of female sex workers, %	25 to 75	10 to 75	Assumed
Duration of PrEP use, years	3	1 to 5	Assumed
PrEP dropout rate, per year	0.17	0.14 to 0.20	7
HIV testing frequency in the PrEP program, per year	2	1 to 12	Assumed
PrEP efficacy against wild‐type HIV, %	75	20 to 90	5, 6
PrEP efficacy against DPV‐resistant HIV, relative to wild‐type, %	100	50 to 100	13
Average PrEP adherence, %^b^	75 (ASPIRE), 49 (RING)	20 to 79	5, 6
Proportion of women who are adherent to PrEP, %^b^	80	33 to 83	5, 6
Adherence level of women who are adherent to PrEP, %^b^	94 (ASPIRE), 61 (RING)	60 to 95	5, 6
Average PrEP effectiveness against wild‐type HIV, %^c^	56 (ASPIRE), 37 (RING)	4 to 71	5, 6
Time until PrEP resistance emerges in an entire HIV‐positive cohort with perfect PrEP adherence, years	0.5	0.25 to 0.75	11
Costs (2017 US$)			
PrEP costs, $ per person‐year	131	119 to 143	28, 29
Outpatient first‐line ART costs (including ARVs), $ per person‐year	279	140 to 419	30, 31, 32
First‐line ARV costs (TDF + 3TC+EFV), $ per person‐year	99	82 to 115	32
Outpatient second‐line ART costs (including ARVs), $ per person‐year	558	279 to 837	30, 31, 32
Second‐line ARV costs (ZDV + 3TC+LPV/r), $ per person‐year	267	259 to 275	32
HIV testing (HIV+ result) and linkage to care, $ per ART initiator	27	Not varied	33
HIV testing (HIV– result), $ per test	12	Not varied	33
Adherence‐support costs, $ per person‐year	50	0 to 200	30, 33, 34
VMMC costs, $ per circumcision	149	135 to 162	35
Annual discount rate, %	3	1 to 5	36

Key references are included here, additional sources for parameter assumptions are provided in the Tables S1 and S2. 3TC, lamivudine; ART, antiretroviral therapy; DPV, dapivirine; EFV, efavirenz; LHS, Latin hypercube sampling; LPV/r, lopinavir/ritonavir; PrEP, pre‐exposure prophylaxis; TDF, tenofovir disoproxil fumarate; VMMC, voluntary medical male circumcision; ZDV, zidovudine.

^a^PrEP efficacy and average adherence were drawn from truncated normal distributions (with medians of 75% efficacy and 62% adherence and the stated ranges) in uncertainty analyses. All other inputs were uniformly distributed; ^b^in uncertainty analysis, the proportion of women who are adherent to PrEP and their level of adherence is calculated from average PrEP adherence by assuming the adherence level among adherent women is proportional to average adherence in the given range; ^c^average PrEP effectiveness against wild‐type virus is calculated as the product of average PrEP efficacy against wild‐type virus and average PrEP adherence.

### 2.2 Model‐based analyses

#### 2.2.1 Reference scenario

Our reference scenario without PrEP reflected the evolution of South African guidelines and targets for HIV treatment 21, including its National Strategic Plans for 2012 to 2016 22 and 2017 to 2022 9. This scenario incorporated the achievement of 80% VMMC coverage among men by 2020, plus universal ART eligibility beginning in September 2016 and reaching 90‐90‐90 targets for ART coverage and virologic suppression by 2020 (90% of HIV‐positive individuals know their status, 90% of whom are on ART, 90% of whom are virally suppressed) and 95‐95‐95 targets by 2030. We assumed that VMMC reduced the risk of HIV acquisition in men by 60% 23 and that suppressive ART reduced the transmission risk by 96% 24 and prolonged the survival of people living with HIV. To achieve the Fast‐Track treatment targets of 73% and 86% overall virologic suppression, we assumed scale‐up of an aspirational concurrent adherence‐support intervention that reduced virologic failure rates by 50% beyond 2020 and by 80% ultimately relative to 2016 rates 25.

#### 2.2.2 PrEP scenarios

We simulated the implementation of DPV‐VR PrEP among women aged 22 to 45 years, in combination with VMMC and universal ART, assuming two PrEP scenarios: either high (75%; ASPIRE scenario) or low (49%; Ring Study scenario) average adherence (Table 1). These scenarios were constructed to match PrEP effectiveness (the product of adherence and efficacy) observed among women aged 22 to 45 in ASPIRE (56%) and the Ring Study (37%), assuming 75% PrEP efficacy against wild‐type HIV 5, 6. In these trials, DPV levels in 80% of trial‐subjects’ plasma samples and in 80% of returned rings indicated VR use. Thus, we assumed that 20% of PrEP users were non‐adherent and never wore the ring, whereas the remaining 80% wore the ring 94% (ASPIRE) or 61% (RING) of the time. Furthermore, we assumed PrEP was equally efficacious against wild‐type and DPV‐resistant HIV, as DPV‐VR use yields genital tract DPV concentrations well above inhibitory levels for DPV‐resistant HIV 13, 19. We examined variable adherence and efficacy in sensitivity analysis (Table 1).

In each PrEP scenario, we simulated four different PrEP implementation strategies: (i) unprioritized, covering 10% to 40% of HIV‐negative women aged 22 to 45; (ii) age‐based, covering 20% to 75% of HIV‐negative women aged 22 to 29 years; (iii) incidence‐based, covering 20% to 75% of HIV‐negative women in age groups and behaviorial risk groups having HIV incidence ≥3% per year 37; or iv) FSW‐PrEP, covering 25% to 75% of HIV‐negative female sex workers (FSWs). We restricted implementation to 22‐ to 45‐year‐old women, as DPV‐VR PrEP was not significantly protective among 18‐ to 21‐year‐old women 5, 6. Our PrEP coverage levels corresponded to implementing PrEP among 5% to 20% of HIV‐negative adult women or 2.5% to 10% of HIV‐negative adults (15 to 54 year‐old), except for FSW‐PrEP reaching just <0.1% coverage of adults due to the group's small size (0.4% of women). Women enrolled in PrEP replaced rings monthly for a duration of three years (PrEP persistence), subject to a competing risk of programmatic dropout at a rate of 0.17 per year 7 with compensatory enrolment to maintain programmatic coverage. HIV testing occurred at enrolment and twice annually thereafter; women with detected HIV stopped PrEP immediately. Women with undetected HIV could enrol inadvertently. PrEP implementation began at 2019, reached target coverage after four years on average, and was then maintained through 2030.

#### 2.2.3 Outcomes and costs

We assumed a healthcare sector perspective and two different simulation time horizons for cost‐effectiveness analysis: PrEP intervention horizon (2019 to 2030) and lifetime horizon of the population extant during PrEP implementation. We included costs associated with VMMC, ART, HIV testing, HIV‐related care and baseline medical costs using published literature from South Africa (Table 1). ART adherence‐support intervention costs were based on community‐based support for adolescents on ART 34. PrEP costs ($131 per person‐year) were based on the fully loaded costs of oral PrEP in South Africa 29, excluding the cost of creatinine testing and replacing the cost of ARVs for oral PrEP with the anticipated cost of the dapivirine vaginal ring ($72 to $96 per person‐year) 28.

We employed gross domestic product (GDP) deflators for South Africa 38 to convert costs to 2017 US dollars, and discounted future costs and life‐years lived at 3% annually. We computed incremental cost‐effectiveness ratios (ICERs) as the change in discounted cost divided by the change in discounted life‐years gained for each intervention relative to the reference scenario or next‐best intervention. We compared ICERs for interventions to both revealed willingness‐to‐pay thresholds for South Africa (approximately $500 39) and to standard thresholds based on one and threefold South Africa's 2017 GDP 36 of about $6200 ($6161 40). We classified an intervention as cost‐saving if it decreased total costs and increased life‐years.

We assessed the budget impact of interventions after ten years of PrEP implementation (by 2029), using undiscounted costs, and an HIV programme perspective that included costs of HIV testing, adult ART and inpatient HIV care, PrEP and VMMC, and that excluded HIV‐unrelated healthcare costs.

#### 2.2.4 Base‐case analyses

We simulated scenarios without or with PrEP in combination with VMMC and ART using point estimates for all model inputs (Table 1). Outcomes were calculated by comparing data from different simulations to the reference scenario.

#### 2.2.5 Uncertainty and sensitivity analyses

We performed 10,000 simulations of the reference scenario and each PrEP strategy using intervention‐related inputs drawn via Latin hypercube sampling (Table 1 and Table S2). Using these data, we calculated outcomes’ medians and interquartile ranges (IQRs), to measure output uncertainty, and standardized regression coefficients (SRCs) to quantify the influence of model inputs on outputs. Response surfaces were used to visualize the effect of influential inputs on key outcomes. We calculated cost‐effectiveness acceptability curves to assess the sensitivity of findings to cost‐effectiveness thresholds 41.

## 3 Results

### 3.1 Base‐case analyses

#### 3.1.1 HIV prevention

Our model projected 412,399 undiscounted new HIV infections during 2019 to 2030 in the reference scenario (Table 2). HIV prevention increased with prioritization and expansion of PrEP (Table 2) and was highest from incidence‐based PrEP (incidence ≥ 3%/year) and next highest with age‐based PrEP (22 to 29 year‐olds). At 15% PrEP coverage of HIV‐negative women (equivalent to 50% age‐ or incidence‐based prioritized coverage and 30% unprioritized coverage of women aged 22 to 45 years), in the ASPIRE scenario (56% effectiveness) incidence‐based PrEP prevented 11.3% of infections compared to the reference scenario, whereas age‐based PrEP prevented 9.6% and unprioritized PrEP prevented 7.1%. FSW‐PrEP (50% FSW coverage) prevented the fewest infections (3.5%) given the group's small size. Table S4 presents results for all PrEP coverage levels. Compared to the ASPIRE scenario, PrEP strategies prevented approximately 40% fewer infections in the Ring Study scenario (37% effectiveness).

**Table 2:** Base‐case impact, cost and drug resistance outcomes

Intervention	Intervention horizon			Lifetime horizon		Prevalent drug‐resistance by 2030	
	New Infections, n	Life‐years lived, thousands	Total costs, millions $	Life‐years lived, thousands	Total costs, millions $	Total DR cases, n	PrEP DR cases, n
No PrEP (reference)	412,399	70,444	23,286	197,641	60,543	88,694	0

Intervention	Infections prevented, %	Life‐years gained, thousands	Cost increases, millions $	Life‐years gained, thousands	Cost increases, millions $	Total DR increase, %	PrEP DR cases, n
ASPIRE PrEP scenario^a^							
Unprioritized PrEP	7.1	6.3	438.2	135	319.6	−1.5	2045
Age‐based PrEP	9.6	8.5	422.5	195	252.5	−1.8	3097
Incidence‐based PrEP	11.3	10.0	418.4	223	220.4	−1.7	3895
PrEP to FSWs	3.5	2.5	−12.1	66	−77.5	1.5	2989
RING PrEP scenario^a^							
Unprioritized PrEP	4.3	4.0	451.2	82	380.8	−0.1	1962
Age‐based PrEP	5.8	5.3	440.2	118	339.1	0.1	2971
Incidence‐based PrEP	6.9	6.2	439.1	134	321.7	0.5	3729
PrEP to FSWs	2.0	1.5	−5.8	37	−42.2	2.0	2669

Absolute outcomes are shown for the reference scenario. Increases relative to the reference scenario are shown for PrEP interventions. Costs and life‐years lived are discounted 3% annually. Costs are in 2017 US$. Results shown are for 50% PrEP coverage of FSWs or 15% PrEP coverage of 15 to 54 women, reaching 30% of women aged 22 to 45 when unprioritized or 50% of prioritized women when age‐based or incidence‐based. Results for all PrEP coverage levels are provided in Table S4. DR, drug resistance; FSW, female sex worker; PrEP, pre‐exposure prophylaxis.

a ASPIRE and the Ring Study (RING) PrEP scenarios simulate 56% or 37% effective PrEP respectively.

#### 3.1.2 HIV survival

Survival gains over the intervention horizon from PrEP implementation were modest, but accumulated over the lifetime horizon (Table 2). Compared to the reference scenario, the discounted life‐years gained over the intervention horizon ranged from 1500 to 2500 by FSW‐PrEP, to 6200 to 10,000 by incidence‐based PrEP, at 15% coverage. Lifetime horizon gains were an order of magnitude larger, ranging from 37,000 to 66,000 by FSW‐PrEP to 134,000 to 223,000 by incidence‐based PrEP. Table S4 presents results for all PrEP coverage levels.

#### 3.1.3 HIV drug resistance

Drug resistance attributable to DPV‐VR PrEP was modest, comprising 1962 to 3895 prevalent cases of resistance or 2% to 4% of cases, compared to ART's contribution of 88,694 cases to total resistance in the reference scenario (Table 2). DPV‐VR PrEP implementation reduced total resistance by 1.5% to 1.8% in the ASPIRE scenario unless prioritized to FSWs, which increased resistance by 1.5%. In contrast, all prioritized PrEP strategies increased resistance in the Ring Study scenario. While resistance increases were modest (≤2%), larger increases occurred when PrEP was prioritized to higher incidence populations.

#### 3.1.4 Budget impact

**Figure 1:**
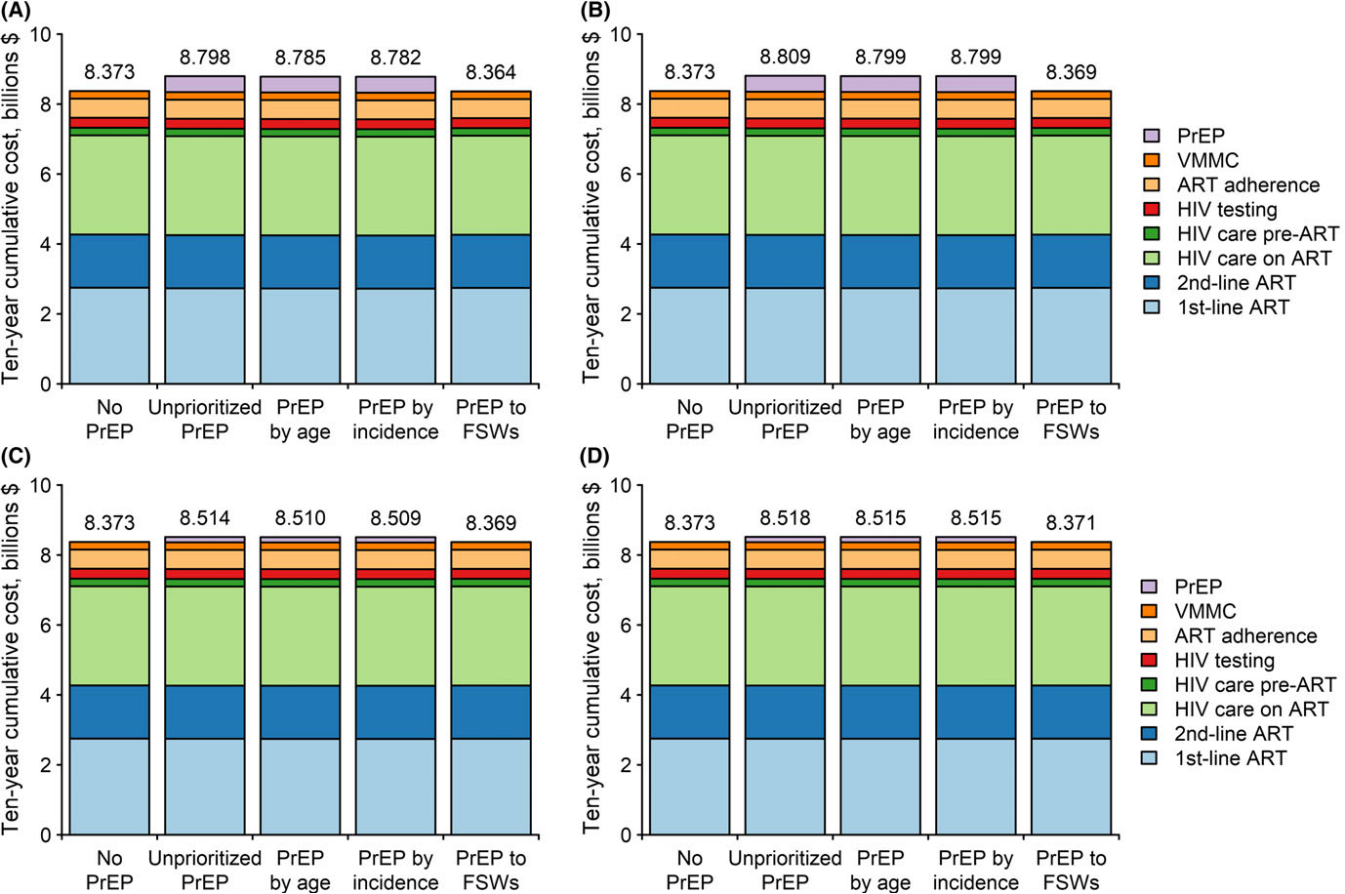
Ten‐year budget impact analysis of dapivirine ring PrEP implementation Costs are in undiscounted 2017 US dollars, and are shown for 15% PrEP coverage of HIV‐negative women (when unprioritized, age‐based, or incidence‐based) in the ASPIRE scenario **(A)**, 15% PrEP coverage in the Ring Study scenario **(B)**, 5% PrEP coverage in the ASPIRE scenario, **(C)** or 5% PrEP coverage in the Ring Study scenario **(D)**. PrEP to FSWs covered 50% of female sex workers in **(A** to **B)** and 25% in **(C** to **D)**. ART, antiretroviral therapy; FSW, female sex worker; PrEP, pre‐exposure prophylaxis; VMMC, voluntary medical male circumcision.

Cumulative ten‐year budget impact is shown in Figure 1. PrEP costs were proportional to PrEP coverage and comparable to VMMC expenditures ($217 million), which at 15% coverage reached $452 to $456 million from unprioritized, age‐based or incidence‐based PrEP implementation (Figure 1A,B) and increased HIV expenditures by 5% compared to the reference. In contrast, at minimal (5%) coverage, PrEP costs fell to $150 to $152 million, with less (1.6% to 1.7%) increase in overall HIV spending (Figure 1C,D). At 25% to 50% coverage, FSW‐PrEP incurred much lower costs of $1.3 to $2.7 million and reduced overall HIV spending slightly (Figure 1A,B,C,D).

#### 3.1.5 Cost‐effectiveness

**Figure 2:**
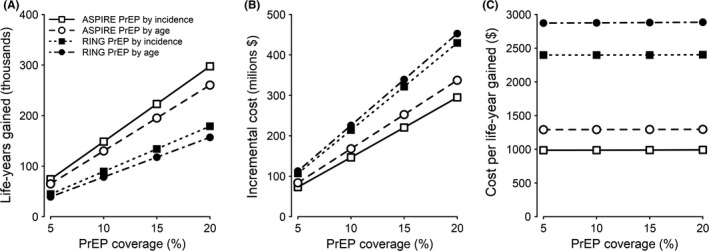
PrEP coverage and cost‐effectiveness Lifetime horizon life‐years gained **(A)**, incremental costs **(B)**, and costs per life‐year gained **(C)** relative to the reference scenario are plotted as a function of coverage for age‐based or incidence‐based PrEP implementation in scenarios of 56% (ASPIRE) or 37% (RING) PrEP effectiveness. PrEP, pre‐exposure prophylaxis.

Both costs and impact from PrEP implementation increased proportionately with coverage (Figure 2A,B), leading to stable cost‐effectiveness ratios across the range of PrEP coverage levels considered (Figure 2C).

**Figure 3:**
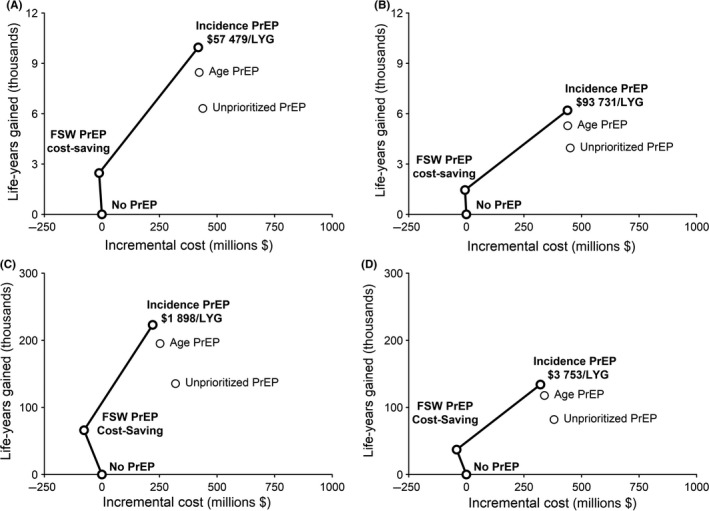
Cost‐effectiveness frontiers of dapivirine vaginal ring PrEP implementation We evaluated incremental costs and life‐years gained in the sexually active population during 2019 to 2030 in ASPIRE **(A)** and Ring Study **(B)** scenarios, and over the lifetime of the PrEP‐exposed cohort in ASPIRE **(C)** and Ring Study **(D)** scenarios. We assessed unprioritized PrEP implementation covering 30% of women aged 22 to 45, age‐based PrEP covering 50% of women aged 22 to 29, incidence‐based PrEP covering 50% of high‐incidence women aged 22 to 45, or PrEP covering 50% of female sex workers aged 22 to 45, in combination with ART implementation reaching UNAIDS Fast‐Track targets 8. Interventions on the cost‐effective frontier are shown in bold, labelled with incremental cost per life‐year gained relative to the next‐best strategy. Note that vertical axis scales differ for intervention **(A** to **B)** and lifetime **(C** to **D)** horizons. ART, antiretroviral therapy; FSW, female sex worker; LYG, life‐years gained; PrEP, pre‐exposure prophylaxis.

Over the intervention horizon, the cost‐effective frontier in the ASPIRE scenario consisted of FSW‐PrEP (cost‐saving relative to the reference scenario), followed by incidence‐based PrEP ($57,479 per life‐year gained relative to FSW‐PrEP) (Figure 3A). These same strategies comprised the cost‐effective frontier in the Ring Study scenario (Figure 3B); where FSW‐PrEP remained cost‐saving, whereas the ICER for incidence‐based PrEP increased ($93,731 per life‐year gained).

ICERs of PrEP implementation fell considerably over the lifetime horizon, as life‐years accumulated and PrEP costs were offset by decreases in ART need. In the ASPIRE scenario, FSW‐PrEP was cost‐saving relative to the reference scenario, whereas incidence‐based PrEP cost $1898 per life‐year gained compared to FSW‐PrEP (Figure 3C). Lifetime horizon results were qualitatively similar in the Ring Study scenario; FSW‐PrEP remained cost‐saving, whereas the incremental cost of incidence‐based PrEP was $3753.

### 3.2 Uncertainty and sensitivity analyses

These analyses include more conservative assumptions, compared to base‐case analyses that simulate the achievement of Fast‐Track ART and VMMC targets (Table 1); which are reflected in the results. Data on HIV prevention, survival and drug resistance for all strategies and horizons are described in Tables S4 to S7 and Figures S3 to S4.

#### 3.2.1 Cost‐effectiveness

**Figure 4:**
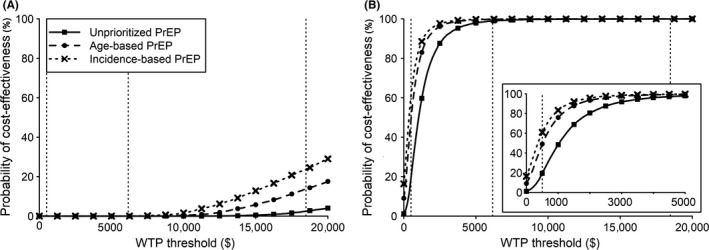
Probability that dapivirine vaginal ring PrEP implementation is cost‐effective at varying thresholds Results are from probabilistic sensitivity analysis. We assessed the cost per life‐year gained, relative to ART implementation without PrEP, in the sexually active population during 2019 to 2030 **(A)** and over the lifetime of the PrEP‐exposed cohort **(B)**. We assessed unprioritized PrEP implementation among women aged 22 to 45, age‐based PrEP among women aged 22 to 29, incidence‐based PrEP among high‐incidence women aged 22 to 45, or PrEP scaled‐up among FSWs aged 22 to 45. PrEP implementation among FSWs was cost‐saving in >99% of simulations (Table S5). Vertical dotted lines highlight a willingness‐to‐pay threshold of $500 39 and thresholds of about one or threefold South Africa's 2017 per‐capita gross domestic product ($6,200 or $18,500 respectively 40). The inset in **(B)** highlights the results for willingness‐to‐pay thresholds of $0 to $5000. ART, antiretroviral therapy; FSW, female sex worker; PrEP, pre‐exposure prophylaxis; WTP, willingness‐to‐pay.

FSW‐PrEP was cost‐saving in nearly 100% of simulations over both intervention and lifetime horizons (Table S5). Over the intervention horizon, ICERs for incidence‐based PrEP relative to the reference scenario were below threefold GDP ($18,500) in 24% of simulations, and less than onefold GDP ($6200) in <0.1% of simulations (Figure 4A), whereas ICERs for unprioritized or age‐based PrEP were less likely to meet these thresholds. Over the lifetime horizon, ICERs of all PrEP strategies were below $6200 in ≥99% of simulations. Incidence‐ and age‐based PrEP were cost‐saving in 16% and 9% of simulations, respectively, and cost <$500 per life‐year gained in 61% and 49% of simulations (Figure 4B); suggesting they may be affordable 39. In contrast, unprioritized PrEP met this threshold in only 19% of simulations. Uncertainty analysis included simulations in which Fast‐Track targets for ART and VMMC were not met, resulting in lower cost‐effectiveness ratios for PrEP compared to base‐case analyses (Figure S4). In simulations where PrEP was not cost‐saving, incidence‐based PrEP cost a median $464 per life‐year gained (IQR: $227 to $869) relative to the reference scenario and $695 (IQR: $369 to $1245) relative to FSW‐PrEP (Table S6). Costs per life‐year gained fell with higher PrEP adherence and efficacy, and rose with improved ART effectiveness for HIV prevention and more aggressive discount rates (Table S7).

## 4 Discussion

We projected the epidemiological impact, budget impact and cost‐effectiveness of DPV‐VR implementation either unprioritized or prioritized by incidence, age or FSW‐status, in combination with VMMC and ART, over different time horizons (2019 to 2030 and lifetime), in KwaZulu‐Natal, South Africa. The important insights from our mathematical modelling study are several. (1) DPV‐VR PrEP could make a substantial contribution to HIV prevention in South Africa if scaled‐up among women at high risk of infection 37, whereas reducing costs if prioritized to FSWs. (2) The changes in overall drug resistance prevalence would be modest from DPV‐VR implementation. (3) Lower PrEP effectiveness (37% vs. 56%) erodes DPV‐VR's impact and cost‐effectiveness and augments drug resistance. (4) PrEP and ART effectiveness and coverage principally determine DPV‐VR's epidemiological and economic impact.

The optimism for HIV PrEP is tempered by the failure of some trials of oral 1 and topical 42 PrEP to demonstrate effectiveness among African women overall, and of ASPIRE and the Ring Study among women ≤21 years old 5, 6. Though daily oral TDF/FTC PrEP is recommended 2 and approved 3 for substantially at‐risk individuals including women in South Africa, adherence is challenging and current use is limited 43. Therefore, extending previous work 15, 16, 44, this study focuses on DPV‐VR PrEP, a candidate for regulatory approval.

We found that the implementation of DPV‐VR PrEP could have substantial population‐level impact on HIV prevention if prioritized to at‐risk women. Over the intervention horizon, compared to the reference scenario without PrEP, at 15% overall coverage of HIV‐negative women, implementation of 56% effective PrEP prevented up to 11% of new infections when prioritized to women at “substantial risk” (HIV incidence ≥3%) 2, whereas prioritization to women aged 22 to 29 regardless of incidence had a similar effect (10% infections prevented). The impact of PrEP prioritized to female sex workers (<4% infections prevented) was limited by their small population size. Survival gains from PrEP by 2030 were modest (≤10,000 life‐years gained) due to the short intervention time horizon, but rose considerably (223,000) over the lifetime horizon. Our base‐case analyses optimistically assume timely and precise attainment of the ambitious treatment and treatment‐mediated prevention targets for 2020 and 2030 27, 43. However, attainment of these treatment targets may be insufficient for population‐level HIV control 45, 46, whereas the attainment itself is jeopardized by suboptimal retention between HIV diagnosis and treatment in South Africa 47; underscoring the need for additional investment in HIV prevention. Thus, a less than optimal Fast‐Track realization will bolster PrEP's epidemiological and economic efficiency (Table S7). PrEP's impact on prevention and survival were sensitive to PrEP efficacy and adherence, which we based on ASPIRE and the Ring Study. We may underestimate PrEP's impact and cost‐effectiveness if individuals adhere more readily to a product with demonstrated effectiveness 48, as indicated by the HOPE open‐label extension of ASPIRE 7.

Changes in prevalent drug resistance from PrEP implementation were modest, consistent with previous findings for oral and injectable PrEP 15, 49. At 37% PrEP effectiveness, prioritized PrEP strategies increased drug resistance at 2030 by ≤2% compared to the reference scenario. Improved PrEP adherence could mitigate these increases, as 56% effective PrEP decreased drug resistance by about 2% unless prioritized to female sex workers. As seen with injectable PrEP 15, PrEP prioritized to female sex workers tended to increase resistance regardless of PrEP effectiveness. Prioritization of FSWs is efficient for HIV prevention because of their high risk of HIV acquisition and onward transmission. However, the same factors elevate their risk of breakthrough infection on PrEP, and subsequent emergence and transmission of drug‐resistant HIV. Nevertheless, the observed increases in resistance were small (1.5% to 2%).

We considered PrEP coverage levels with budget requirements comparable to VMMC spending, representing 70% to 210% of VMMC costs unless prioritized to female sex workers. Excluding FSW‐PrEP, PrEP implementation increased the baseline HIV expenditures (VMMC plus inpatient and outpatient HIV care) by 5% over ten years when scaled‐up to cover 15% of adult HIV‐negative women, whereas lower (5%) PrEP coverage levels raised overall expenditures by <2% with minimal attenuation of cost‐effectiveness. Contrastingly, FSW‐PrEP decreased HIV expenditures overall. Thus, FSW‐PrEP was cost‐saving over both intervention and lifetime horizons of PrEP scenarios, in qualitative agreement with previous modelling of oral, injectable and vaginal ring PrEP 16, 50, 51, 52. Meanwhile, incidence‐based PrEP was more cost‐effective than age‐based or unprioritized PrEP. Its lifetime cost per life‐year gained relative to FSW‐PrEP was $1898 to $3753 depending on PrEP effectiveness, which compares favourably to South Africa's per‐capita gross domestic product (approximately $6200 40). However, without increases in funding levels and decreases in PrEP commodity costs ($4 to $6 per ring 28), this may not be affordable given that PrEP may need to cost less than $500 per life‐year gained to compete with South Africa's other HIV health investments. Contrastingly, if Fast‐Track targets are not achieved, incidence‐ and age‐based PrEP may become more affordable (Tables S5 to S6) 39.

As countries contemplate PrEP implementation, questions of impact and cost‐effectiveness, which mathematical modelling can address, must be considered alongside issues of equity and acceptability. While DPV‐VR PrEP prioritized by incidence or age may be less cost‐effective than ART, VMMC or condom provision 39, it could be a viable option for HIV‐negative women who may not benefit directly from ART or VMMC, nor ably negotiate condom use 53.

Incidence‐based prioritization maximized PrEP's impact among the strategies we considered. However, this strategy relies on accurate identification, enrolment and retention of at‐risk women, which may be logistically challenging 54, 55. Young women may be easier to identify and reach; DPV‐VR PrEP, at 50% prioritized coverage among 22‐ to 29‐year‐old women, had only marginally less impact (6% to 10% vs. 7% to 11% infections averted) and cost‐effectiveness ($1300 to $2900 vs. $1000 to $2400 per lifetime life‐year gained relative to no PrEP) than incidence‐based PrEP, due to high HIV incidence in KwaZulu‐Natal (approximately 4%) 14 and South Africa overall (2.75% to 3.5%) 14 among women aged 20 to 29.

We studied DPV‐VR PrEP in combination with recommended first‐ and second‐line ART regimens per South African treatment guidelines 21. We did not model interim guidelines that recommend dolutegravir‐containing regimens for people newly initiating first‐line ART or as second‐line therapy for patients failing non‐dolutegravir first‐line regimens 56. While dolutegravir implementation in South Africa appears imminent 57, incorporation of this scenario in our modelling is challenging for several reasons. The timing and scale of dolutegravir rollout is not precisely known 58, 59, and the policy may shift from implementation among people newly initiating treatment to use in all ART patients 60. Several gaps in the evidence base need to be addressed 61 that will also inform future modelling; in particular, more data are needed to determine the risk of adverse birth outcomes among women who initiate dolutegravir‐containing regimens before conception, and the effect of nucleoside reverse transcriptase inhibitor resistance on the long‐term efficacy of dolutegravir‐based regimens in first‐ and second‐line ART. Our present analyses do suggest that widespread switch to more potent 62, cheaper ($75/person‐year 57 vs. $99/person‐year in our study 32) ART would augment ART's cost‐effectiveness and attenuate PrEP's cost‐effectiveness, whereas the reduction in cross‐resistance between first‐line ART and DPV‐VR PrEP would further limit the modest resistance from DPV‐VR implementation.

This study has several limitations. Precise details of our model's projections will be affected by variations in its structural and parameter assumptions, especially those regarding sexual behaviour. Nevertheless, we used rigorous model construction, calibration, parameterization and analysis. We did not study PrEP among women aged 21 and younger, as ASPIRE and the Ring Study found that DPV‐VR was not effective at younger ages. However, DPV‐VR PrEP may also be cost‐effective among these women if our PrEP‐related assumptions are valid for younger age‐groups. Because DPV‐VR PrEP is yet to be implemented, its real‐world effectiveness and resistance potential remain unknown. To account for this knowledge gap, we considered different scenarios and strategies in our base‐case analyses and explored wide parameter estimate ranges in sensitivity analyses. Our assumptions regarding cross‐resistance between ART and PrEP and the potential efficacy of PrEP and ART against cross‐resistant HIV are primarily informed by laboratory studies involving a limited set of 102 HIV isolates from patients failing first‐line ART in South Africa 13; nevertheless, we examined a broad estimate range in uncertainty and sensitivity analyses. Finally, our modelling context is the mature, generalized, high‐prevalence HIV epidemic in KwaZulu‐Natal, South Africa. Thus, our quantitative findings may not generalize directly to other contexts. Nonetheless, the qualitative insights from our modelling are likely to be robust.

## 5 Conclusions

Implementation of dapivirine vaginal ring PrEP in KwaZulu‐Natal among female sex workers would be cost‐saving, whereas prioritization to women at substantial risk or to women aged 22 to 29 could have substantial impact on HIV prevention at affordable economic value. PrEP implementation will have limited effect on HIV drug resistance.

## Competing interests

RG, UMP, GH, KJP and ULA report grants from the Bill and Melinda Gates Foundation during the conduct of the study. JWM and UMP report grant support from USAID during conduct of the study. JWM reports personal fees from University of Pittsburgh, personal fees from Gilead Sciences, and has stock options in Cocrystal Pharma, Inc., outside the submitted work, and has been issued patent #8815,829.

## Authors’ contributions

RLG contributed to model formulation, implementation and simulation, data compilation, analyses and interpretation, and writing of first and subsequent manuscript drafts. YD assisted with data verification and interpretation and contributed to manuscript writing. UMP, KJP and JWM generated laboratory data on NNRTI cross‐resistance and estimates of *in vitro* DPR efficacy as PrEP, and assisted in interpretation and communication of findings and manuscript writing. GH and EE facilitated access to and use of computational resources and assisted in manuscript review. ULA conceived and designed the project and contributed to model formulation and experimental design, analyses and interpretation of data, and writing of first and subsequent manuscript drafts.

## Supporting information


**Table S1.** Model behaviorial, epidemiological and demographic input parameters
**Table S2.** Model intervention‐related input parameters
**Table S3.** Model drug resistance dynamics
**Table S4.** Results of base‐case analysis
**Table S5.** Uncertainty analysis outcomes
**Table S6.** PrEP cost‐effectiveness in uncertainty analysis simulations
**Table S7.** Results of sensitivity analysis: drivers of key model outcomes
**Figure S1.** Simplified model flow diagram of HIV disease progression and ART use.
**Figure S2.** Flow diagram of model drug resistance dynamics.
**Figure S3.** Changes in drug resistance after PrEP implementation.
**Figure S4.** Lifetime horizon cost‐effectiveness of PrEP implementation.
