# Supplementary material for: Dapivirine vaginal ring for HIV prevention: modelling health outcomes, drug resistance and cost‐effectiveness
Source: J Int AIDS Soc. 2019 May 10;22(5):e25282. doi: 10.1002/jia2.25282 (PMC6510112; doi:10.1002/jia2.25282)
Supplement: Supplementary file 1 — Table S1. Model behaviorial, epidemiological and demographic input parameters Table S2. Model intervention‐related input parameters Table S3. Model drug resistance dynamics Table S4. Results of base‐case analysis Table S5. Uncertainty analysis outcomes Table S6. PrEP cost‐effectiveness in uncertainty analysis simulations Table S7. Results of sensitivity analysis: drivers of key model outcomes Figure S1. Simplified model flow diagram of HIV disease progression and ART use. Figure S2. Flow diagram of model drug resistance dynamics. Figure S3. Changes in drug resistance after PrEP implementation. Figure S4. Lifetime horizon cost‐effectiveness of PrEP implementation. [file JIA2-22-e25282-s001.docx]

**Supplemental Text: Dapivirine Vaginal Ring for HIV Prevention: Modelling Health Outcomes, Drug Resistance and Cost-Effectiveness**

Robert Glaubius^1^; Yajun Ding^2^; Kerri J. Penrose^3^; Greg Hood^4^; Erik Engquist^5^; John W. Mellors^3^; Urvi M. Parikh^3^; Ume L. Abbas^1,2^

^1^Departments of Quantitative Health Sciences and Infectious Disease, Cleveland Clinic, Cleveland, OH 44195

^2^Department of Medicine, Section of Infectious Diseases and Department of Molecular Virology and Microbiology, Baylor College of Medicine, Houston, TX 77030

^3^Division of Infectious Diseases, School of Medicine, University of Pittsburgh, Pittsburgh, PA 15261

^4^Pittsburgh Supercomputing Center, Carnegie Mellon University, Pittsburgh, PA 15213

^5^Center for Research Computing, Rice University, Houston, TX 77005

Robert Glaubius is currently at Avenir Health, Glastonbury, CT 06033

Yajun Ding is currently at SMS Assist LLC, Chicago IL 60610, USA

1 INTRODUCTION 3

2 MODEL OVERVIEW 3

2.1 Demography and sexual behavior 3

2.2 HIV natural history 3

2.3 HIV drug resistance 4

2.4 Interventions 4

2.4.1 PrEP implementation 4

2.4.2 ART implementation 5

2.4.3 Medical male circumcision implementation 5

2.5 Analyses 5

3 MODEL EQUATIONS 6

3.1 Ordinary differential equations of model compartments 7

3.2 HIV transmission 11

3.2.1 Force of infection 11

3.2.2 Mixing and balancing 11

3.2.3 HIV transmission within partnerships 12

3.3 Intervention implementation 13

3.3.1 Condom use 13

3.3.2 Voluntary medical male circumcision 13

3.3.3 Antiretroviral therapy 13

3.3.4 Pre-exposure prophylaxis 14

4 MODEL CALIBRATION 15

5 SUPPLEMENTARY SENSITIVITY AND UNCERTAINTY ANALYSIS RESULTS 15

5.1 HIV prevention and survival 15

5.2 HIV drug resistance 15

5.3 PrEP cost-effectiveness 15

6 SUPPLEMENTARY TABLES 17

7 SUPPLEMENTARY FIGURES 27

# INTRODUCTION

In this study we examine the epidemiological and drug resistance outcomes of implementing dapivirine (DPV) vaginal ring pre-exposure prophylaxis (PrEP) alongside antiretroviral treatment (ART) and voluntary medical male circumcision (VMMC) in the KwaZulu-Natal province of South Africa. We extend and analyze a detailed mathematical model of the heterosexual HIV epidemic in KwaZulu-Natal, consisting of coupled nonlinear differential equations that describe demographic and epidemiological stratifications based on gender, age, sexual behavior, VMMC status, PrEP and ART use, HIV infection status and disease progression, and HIV drug resistance, to examine the implementation of DPV PrEP. Details of model design and calibration are described elsewhere [1, 2] and in the following, where we focus on our modeling of dapivirine ring PrEP implementation and HIV drug resistance. Section 2 provides a model overview. Complete model equations are provided in Section 3. Model calibration is summarized in Section 4. Supplementary results are presented in Section 5. Model input parameters are provided in Supplementary Tables 1 and 2. Supplementary Table 3 summarizes HIV drug resistance dynamics in our model. Supplementary Figure 1 shows the natural history of HIV infection with ART and PrEP use. Supplementary Figure 2 illustrates model HIV drug resistance dynamics. Supplementary results are provided in Supplementary Tables 4-7 and Supplementary Figures 3 and 4.

# MODEL OVERVIEW

## Demography and sexual behavior

Men and women enter the model upon sexual debut at age 15 and exit the sexually active population at age 55 or death. The population grows from about 2.5 million people at 1978. The sexually active population is stratified into one-year age bands (ages 15, 16, …, 54). Population growth trends, overall and by age and sex, are fit to estimates from Statistics South Africa [3].

Individuals are stratified into four sexual activity levels (least, low, medium, and high) that are distinguished by increasing rates of partner change. The high sexual activity level represents female sex workers (FSWs) and their male clients. The model represents details of balance in the supply and demand of sexual partnerships [1, 2], including those of FSWs [4]*.*

We simulate HIV transmission within heterosexual partnerships. Partnership formation follows a hybrid sexual mixing pattern that combines random mixing, preferential mixing by sexual activity level, and preferential mixing by age (Section 3.2.2). Sexual partnerships are categorized as regular, casual, or short. *Regular* partnerships involve at least one partner from the least or low sexual activity level. These partnerships have relatively long duration, and consequently involve a larger number of sexual acts. *Short* partnerships form between men and women in the high sexual activity level, and involve fewer sexual acts. All other partnerships are *casual*, having intermediate duration. Condoms use is relatively infrequent in regular partnerships [5]. We assume that condom use increases over time and then stabilizes across all partnership types based on historical data [5, 6], and do not assume future increase or decrease [7].

## HIV natural history

HIV-positive individuals pass through six successive stages of infection followed by death from AIDS (Supplementary Figure 1). The stages include acute infection pre- or post-seroconversion, chronic infection with >500, 351–500, or 201–350 CD4 cells/μL, and AIDS with ≤200 CD4 cells/μL. We assume HIV infection is undetectable by routine HIV testing during the window period before seroconversion, while individuals in subsequent stages have established, detectable infection. Stages are characterized by a disease progression rate and the probability of HIV transmission per sexual act. HIV transmission risks reflect changes in HIV plasma RNA (viral load) [8, 9], and are highest during acute infection, decrease to lower levels during chronic infection, then climb during AIDS (Supplementary Table 1). We assume women with more advanced HIV infection are less likely to initiate and more likely to cease sex work [10, 11], and that men and women with AIDS reduce their partner change rates due to poor health [12].

## HIV drug resistance

We stratify HIV-positive individuals by antiretroviral (ARV) use (not on ARVs, on PrEP, or on ART), HIV drug susceptibility (drug-sensitive or drug-resistant), type of drug resistance (transmitted or acquired), and virus population dynamics of drug-resistant HIV (majority or minority). For parsimony, we primarily focus on the presence or absence of resistance to the non-nucleoside reverse transcriptase inhibitors (NNRTIs) used for first-line ART, resistance to DPV, or cross-resistance between the two, but we do not characterize specific resistance-associated mutations. The virus population in an HIV-positive individual is comprised of a set of related variants, termed *quasispecies*. Drug-sensitive wild-type virus constitutes the majority before an antiretroviral-naïve virus population is exposed to ARVs. After exposure to ARVs, either drug-sensitive or drug-resistant virus may predominate. *Acquired drug resistance* (ADR) may occur from the selection of drug-resistant variants in individuals with drug-sensitive virus taking ARVs. *Transmitted drug resistance* (TDR) occurs among recipients who are infected by donors with a majority population of drug-resistant variants. Majority drug-resistant variants may revert to drug-sensitive type if drug pressure is removed, either through discontinuation of ARVs [13, 14] or transmission to a new host [15, 16]. Reversion may occur because of overgrowth of resistant variants by wild-type variants or genetic reversion of the resistant variants to wild-type [17]. Following reversion, drug resistance persists as a minority population [18]. Compared to individuals with wild-type virus, individuals with predominant drug-resistant virus may have reduced infectiousness on average, due to decreased transmission fitness [19, 20], continued ARV activity (if on ART) [21] or lower virus replicative capacity [13, 14, 22].

Clinical trials (ASPIRE and the Ring Study) did not find any significant selection of majority or minority DPV-resistant virus in blood, though selection in the genital tract has not been excluded. Selection of DPV-resistance may not occur in the genital tract with sustained high local drug levels; however, selection is likely with levels inadequate for viral suppression from DPV-VR use among women having breakthrough or undetected baseline infection. Therefore, our model tracks virus population dynamics in both blood and genital bodily compartments of HIV-positive individuals (Supplementary Table 3 and Supplementary Figure 2). We assume newly-infected individuals have the same variant in blood and genital compartments, since a single transmitted virus establishes most new HIV infections [23-25]. Whereas PrEP can only promote drug resistance in the genital tract in our model, resistance selected on ART emerges simultaneously in both bodily compartments [26-28]. We assume the predominant HIV variant in blood determines an individual’s rate of disease progression [29, 30] and ART virologic response [31], while the genital tract variant determines the risk of HIV transmission [32]. Model dynamics of drug resistance transmission, emergence, and reversion are enumerated in Supplementary Table 3 and illustrated in Supplementary Figure 2.

Our model distinguishes HIV variants by the presence or absence of resistance to NNRTI-based first-line ART, resistance to protease inhibitor (PI)-based second-line ART, resistance to DPV PrEP, or cross-resistance between DPV PrEP and first-line ART. We assume that 80% (range: 70%–100%) of ART patients who acquire resistance to first-line ART develop cross-resistance to PrEP [33], if they initiated treatment with PrEP-sensitive infection. By contrast, all patients who acquire ART resistance after initiating treatment with PrEP-resistant HIV develop cross-resistance.

## Interventions

### PrEP implementation

We simulate DPV PrEP rollout beginning at 2019 and continuing until the end of 2030. Women are tested for HIV at enrollment; women without detectable HIV infection initiate PrEP. Women enrolled in PrEP receive routine HIV testing; those who test positive discontinue PrEP immediately. In base-case analyses women complete their course of vaginal PrEP after three years on average (PrEP persistence), though some drop out early (rate: 0.17 per year), with continued enrollment to maintain coverage. We restrict enrollment to women aged ≥22 years, as DPV PrEP was not effective among younger women in ASPIRE and The Ring Study [34, 35].

In base-case analyses PrEP is 75% efficacious against wild-type HIV [34-36] and PrEP has equal efficacy against wild-type and PrEP-resistant HIV, as DPV levels achieved after 28 days of continuous ring use [37] were at least 23-fold higher than all IC_90_ (the DPV concentration required to reduce HIV replication by 90%) values observed in a study of 102 isolates from individuals failing first-line ART with NNRTI resistance in South Africa [33]. We explored 0%–50% reductions in PrEP efficacy against PrEP-resistant and cross-resistant HIV in sensitivity analysis. Animal studies suggest that topical PrEP use by HIV-positive individuals may reduce genital HIV shedding [38], potentially reducing HIV transmission by HIV-positive PrEP users. We assume no reduction in HIV transmission risk from HIV-positive PrEP users in base-case analyses, but include a 0%–50% reduction in HIV transmission among acutely HIV-positive women on PrEP in sensitivity analyses.

We model PrEP adherence as the proportion of time women wear the ring, and stratify PrEP users as either adherent (>0% adherence) or non-adherent (0% adherence). We define average PrEP effectiveness as the product of PrEP’s wild-type efficacy and average PrEP adherence. We simulate base-case scenarios of higher or lower average adherence (75% or 49%, respectively), with corresponding average PrEP effectiveness of 56% or 37%, respectively corresponding to estimates from the ASPIRE and Ring Study among women aged 22-45 years [34, 35] (Supplementary Table 2).

### ART implementation

We simulate an ART scenario that reflects the evolution of South African HIV treatment guidelines [39] and adoption of UNAIDS Fast-Track treatment targets [40, 41]. ART uptake rates are stratified by disease stage, and historic uptake rates are calibrated to match coverage trends in KwaZulu-Natal [42]. ART scale-up in our model reflects the adoption of increasing CD4 cell count thresholds for treatment eligibility over time, culminating in universal treatment regardless of CD4 count starting in September of 2016. To simulate achievement of Fast-Track treatment targets, we assume scale-up of routine HIV testing in the sexually active population and immediate ART initiation among individuals with detected HIV infection. Routine HIV testing begins on September 1, 2016, and scales up such that 81% and 90% of adults living with HIV are on ART by 2020 and 2030, respectively.

We assume ART reduces the risk of wild-type HIV transmission by 96% (range: 73%–99%) [43]. We distinguish individuals receiving first-year ART from those in subsequent years of ART in terms of their HIV-related mortality, dropout, and virologic failure. ART initiation at higher CD4 cell counts improves survival compared to initiation at CD4 ≤ 200 cells/μL [44-46]. Patients may experience virologic failure on ART due either to resistance emergence or non-adherence [47]. Pre-existing PrEP resistance or transmitted ART resistance increases the risk of virologic failure with resistance emergence among individuals who adhere to ART [31]. Patients on failing NNRTI-based first-line ART may switch to PI-based second-line ART at rates consistent with South African guidelines for management of treatment failure [39].

### Medical male circumcision implementation

We simulate voluntary medical male circumcision (VMMC) scale-up in KwaZulu-Natal beginning in 2011, reaching 23% at the beginning of 2012 [7] and 80% (range: 60%–85%) by 2020 [48, 49]. Circumcision reduces the probability of HIV acquisition per sexual contact by 60% [50-52], but we assume circumcision has no effect on an HIV-positive man’s probability of HIV transmission to a woman [53].

## Analyses

Model base-case, uncertainty and sensitivity analyses are described in the main manuscript under Methods and model outcomes are provided under Results in the main manuscript and supplementary Sections 5-7.

We assume two different simulation time horizons for our analyses: PrEP intervention horizon and lifetime horizon. The rationale for this approach is that the HIV preventive impact of PrEP is best assessed over the shorter intervention horizon, while the impact on HIV survival is appreciated over the longer lifetime horizon. Thus, economic evaluations of HIV prevention interventions are challenging because costs are incurred simultaneously with service delivery, but improved survival and reductions in treatment need manifest on longer timescales. Hence, we report impact and cost-effectiveness over the two time horizons: a shorter-term PrEP intervention horizon (2019-2030) and a long-term lifetime horizon. We evaluated lifetime horizon outcomes among the model population that was sexually active during the intervention period. We compartmentalized the model population according to sexual debut before or after 2030, and ran model simulations until the population that debuted before 2030 had died.

# MODEL EQUATIONS

The model population is stratified according to infection status (susceptible or HIV-positive). We use subscripts and superscripts to indicate population stratifications within compartments of susceptible (*X*) or HIV-positive (*Y* or *Z*) individuals:

$X_{g,a,k}^{j,z}$ Compartments of susceptible individuals are stratified by VMMC status *j* (*j* = 1, uncircumcised; *j* = 2; circumcised), ARV status *z* (*z* = *N*, not on ARVs; *z* = *P*_1_, non-adherent to PrEP; *z* = *P*_2_, adherent to PrEP), gender *g* (*g* = 1, women; *g* = 2, men), age *a* (15, 16, …, 54, 55+), and sexual activity level *k* (*k* = 1, least; *k* = 2, low; *k* = 3, medium; *k* = 4, high).

$Y_{g,a,k,h}^{j,z,v,v^{'}}$ Compartments of HIV-positive individuals who are ART-naïve or who have initiated first-line ART are also stratified by VMMC status *j*, gender *g*, age *a*, and sexual activity level *k*, as well as by HIV disease stage *h* (*h* = 1, acute infection pre-seroconversion; *h* = 2, acute infection post-seroconversion; *h* = 3, chronic infection with CD4 > 500 cells/µL; *h* = 4, chronic infection with 351–500 CD4 cells/µL; *h* = 5, chronic infection with 201–350 CD4 cells/µL; *h* = 6, AIDS), ARV status *z* (*z* = *N*, *P*_1_, or *P*_2_ as above; *z* = *T*_1_, adherent to first-year ART; *z* = *T*_2_, adherent to subsequent years of ART; *z* = *T*_3_, non-adherent to ART), and HIV variants in blood *v* and the genital tract *v’*.

$Z_{g,a,k,h}^{j,z,v,v^{'}}$ Compartments of treatment-experienced individuals who have initiated second-line ART have the same stratifications as other HIV-positive individuals above; however, the ARV status *z* may only be *z* = *N*, not on ARVs; *z* = *T*_2_, adherent to ART; or *z* = *T*_3_, non-adherent to ART.

Our model tracks seventeen HIV variants (*W*, *R*_1_, *C*_1_, *S*_1_, *Q*_1_, *R*_2_, *C*_2_, *S*_2_, *Q*_2_, *r*_1_, *c*_1_, *s*_1_, *q*_1_, *r*_2_, *c*_2_, *s*_2_ or *q*_2_). *W* denotes wild-type HIV; $v = R_{\bullet}$ and $C_{\bullet}$ denote resistance to first-line ART, either without or with cross-resistance to PrEP, respectively; $v = S_{\bullet}$ denotes resistance to second-line ART, and $v = Q_{\bullet}$ denotes resistance to PrEP. Capital letters (*R*, *C*, *S*, *Q*) indicate majority drug resistant variants while lower-case letters (*r*, *c*, *s*, *q*) denote corresponding reverted-to-wild-type variants. Subscripts distinguish transmitted resistance from acquired resistance (e.g., *R*_1_ and *R*_2_, respectively).

Demographic and behavioral dynamics in the model equations below are denoted by $\Gamma X_{g,a,k}^{j,z}$ or $\Gamma Y_{g,a,k,h}^{j,z,v,v^{'}}$ for compartments of susceptible or HIV-positive individuals, respectively:

$\Gamma X_{g,a,k}^{j,z}=\sum_{k^{'}=1}^{4} \nu_{g,k^{'},k}X_{g,a,k^{'}}^{j,z}-\left( \mu_{g,a}+1+\sum_{k^{'}=1}^{4} \nu_{g,k,k^{'}} \right)X_{g,a,k}^{j,z}+\left\{ \begin{matrix} f\left( t \right)\varphi_{g,k}U_{g,j}^{M}(t), & a=15,z=N \\ 0, & a=15,z\neq N \\ X_{g,a-1,k}^{j,z}, & a>15 \end{matrix} \right.$ (1)

$\Gamma Y_{g,a,k,h}^{j,z,v,v^{'}}=\sum_{k^{'}=1}^{4} \nu_{g,k^{'},k}^{h,z}Y_{g,a,k^{'}}^{j,z,v,v^{'}}-\left( \mu_{g,a}+1+\sum_{k^{'}=1}^{4} \nu_{g,k,k^{'}}^{h,z} \right)Y_{g,a,k,h}^{j,z,v,v^{'}}+\left\{ \begin{matrix} 0, & a=15 \\ Y_{g,a-1,k,h}^{j,z,v,v^{'}}, & a>15 \end{matrix} \right.$ (2)

Demographic and behavioral dynamics of $\Gamma Z_{g,a,k,h}^{j,z,v,v^{'}}$ are defined as in Equation 2. Equations 1 and 2 consist of sexual debut ($f$), aging (rate: 1 per year), background mortality ($\mu$), and changes in sexual behavior ($\nu$). Individuals enter the model HIV-susceptible upon sexual debut at age 15; $\varphi_{g,k}$ is the proportion of model entrants having gender *g* and sexual activity level *k*. The proportion of individuals who debut with circumcision status *j* at time *t* is $U_{g,j}^{M}(t)$ ($U_{1,2}^{M}\left( t \right)=0$, as we do not model female circumcision). Rates of sexual behavior change represent discontinuation of commercial sex work by FSWs, and replenishment of the female sex worker population from lower sexual activity levels. Women with advanced HIV infection leave sex work more rapidly and are less likely to initiate sex work than their HIV-negative counterparts [10, 11].

The rate individuals enter the sexually active population, $f\left( t \right)$, is proportional to $N(t)$, the size of the model population aged 15–54 years at time *t*:

$f\left( t \right)=\left[ 2\left( 0.058+\frac{0.038-0.058}{1+e^{-(t-1978)/10}} \right)-0.038 \right]\times N(t)$ (3)

The coefficients in Equation 3 are tuned to fit KwaZulu-Natal’s population size and age distribution [2].

## Ordinary differential equations of model compartments

Our mathematical model is comprised of 59 ordinary differential equations (Equations 4–62), presented below using Newton’s dot notation ($\dot{X}\equiv dX/dt$) for temporal derivatives. For clarity, compartment indices for sex, age, sexual activity level and VMMC status are condensed into an index vector $i=(g,a,k,j)$; e.g., $Y_{g,a,k,h}^{j,z,v,v^{'}}$ is abbreviated as $Y_{i,h}^{z,v,v^{'}}$.

Equations 4 and 5 describe the dynamics acting on HIV-susceptible individuals. PrEP-naïve women may initiate PrEP at rate $u_{i}^{P}$ or become infected at rate $\lambda_{i}^{N,V}$ by HIV variant $V$, which may be any of the five variants $\mathbf{V}=\{W,R_{1},C_{1},S_{1},Q_{1}\}$. Women who initiate PrEP enter adherence stratum $P_{z}$ with probability $\chi_{z}$ ($\chi_{1}+\chi_{2}=1)$, stop PrEP at rate $\eta^{P}$, or become infected at rate $\lambda_{i}^{P_{z},V}$.

Equations 6–17 describe the dynamics acting on HIV-positive individuals during pre-seroconversion acute infection. In these equations, $V\in\left\{ W,R_{1},C_{1},S_{1},Q_{1} \right\}$ denotes any transmitted HIV variant, while $v\in\left\{ r_{1},c_{1},s_{1},q_{1} \right\}$ denotes corresponding reverted-to-wild-type variants. Newly infected individuals have the same HIV variant in blood and genital bodily compartments. Individuals with HIV variant $V$ in blood experience disease progression at rate $\gamma_{h,V}$. HIV-positive women who have not seroconverted may start or stop PrEP as though HIV-negative; PrEP use by HIV-positive women may either maintain transmitted resistance (in case of variants $C_{1}$ or $Q_{1}$) or cause PrEP resistance to emerge at rate $\zeta_{V}^{P_{z}}$ (in case of other variants) in the genital tract. Meanwhile, transmitted resistant variants in blood may revert at rate $\xi_{V}$ regardless of PrEP status ($\xi_{W}=0$ for wild-type HIV). Transmitted resistance reverts synchronously in blood and the genital tract i) while PrEP-naïve, or ii) while on PrEP with DPV-sensitive variants $R_{1}$ or $S_{1}$. Acquired PrEP resistance (variant $Q_{2}$) may revert (variant $q_{2}$) after stopping PrEP, but re-emerges rapidly with renewed PrEP exposure. HIV variants that result from resistance emergence and reversion in the model are enumerated in Supplementary Table 3.

Equations 18–29 define the dynamics acting on ART-naïve individuals with established HIV infection. In these equations, $V\in\left\{ W,R_{1},C_{1},S_{1},Q_{1} \right\}$ denotes any transmitted HIV variant, while $v\in\left\{ r_{1},c_{1},s_{1},q_{1} \right\}$ denotes corresponding reverted-to-wild-type variants. Equations for these compartments are similar to those for pre-seroconversion acute infection with the following exceptions. Women with established HIV infection do not initiate PrEP; however, women who seroconvert on PrEP continue to receive new rings until they test positive for HIV at rate $\sigma$. Individuals with established infection who are not on PrEP may initiate first-line antiretroviral therapy at stage-specific rates $u_{h}^{T}$. ART patients who drop out (rate $\eta^{T_{\bullet}}$) before acquiring drug resistance return to ART-naïve status.

Equations 30–47 describe the dynamics of first-line ART use. Individuals may have blood HIV variants $V\in\left\{ W,R_{1},C_{1},S_{1},Q_{1} \right\}$ (or corresponding minority variants $v\in\left\{ r_{1},c_{1},s_{1},q_{1} \right\}$) and genital tract variants $V^{'}\in\left\{ W,R_{1},C_{1},S_{1},Q_{1},Q_{2} \right\}$ (or corresponding minority variants $v^{'}\in\left\{ r_{1},c_{1},s_{1},q_{1},q_{2} \right\}$) at first-line ART initiation. Individuals on ART are stratified into three ARV states ($z=T_{1}$, adherent to first-year ART; $z=T_{2}$, adherent to subsequent years of ART; or ${z=T}_{3}$, non-adherent to ART). Individuals who are virologically suppressed on ART (i.e., who are adherent and have not acquired ART resistance) may experience HIV-related mortality at rate $\mu_{h}^{T_{\bullet}}$ or virologic failure at rate $\omega_{V}^{T_{\bullet}}$. A proportion $\psi_{V}^{T_{\bullet}}$ of virologic failures are due to resistance emergence, while the rest ($1-\psi_{V}^{T_{\bullet}}$) are due to non-adherence. Virologic failure rates depend on the HIV variant present in blood. A proportion $(x_{V,V^{'}}$) of individuals who acquire drug resistance on first-line ART develop cross-resistant variant $C_{2}$, while the rest acquire PrEP-sensitive variant $R_{2}$. ART-resistant variants emerge simultaneously in blood and genital compartments, and may revert to wild-type after ART dropout. Rates of ART non-adherence are calculated to be the same regardless of baseline HIV variant. Non-adherent individuals experience HIV disease progression and drug resistance reversion as though not on ART. Patients who fail first-line ART, whether due to non-adherence or resistance emergence, switch to second-line ART at rate $u^{Z}$.

The dynamics of second-line ART use are defined in equations 48–62. Individuals in these equations may have majority HIV variants $V\in\left\{ W,R_{1},C_{1},S_{1},Q_{1},R_{2},C_{2} \right\}$ or minority variants $v\in\left\{ r_{1},c_{1},s_{1},q_{1},r_{2},c_{2} \right\}$ in blood; likewise, they may have variants $V^{'}\in\left\{ W,R_{1},C_{1},S_{1},Q_{1},R_{2},C_{2},Q_{2} \right\}$ or $v^{'}\in\left\{ r_{1},c_{1},s_{1},q_{1},r_{2},c_{2},q_{2} \right\}$ in the genital tract. Individuals who stopped first-line ART due to resistance have concordant ART-resistant variants, either $R_{2}$ or $C_{2}$, in both bodily compartments, while individuals switched for non-adherence have other, potentially discordant, variants. Individuals on second-line ART may drop out (rate $\eta^{Z}$) or experience HIV-related mortality ($\mu_{h}^{Z}$) or virologic failure ($\omega_{V}^{Z}$). Virologic failure may occur because of new resistance emergence (proportion $\psi_{V}^{Z}$) or non-adherence ($1-\psi_{V}^{Z}$); rates of resistance emergence are determined by the HIV variant in blood. We assume resistance to PrEP or first-line ART may revert to wild-type on second-line ART [54].

**Compartments of HIV-susceptible individuals**

$\dot{X}_{i}^{N}=\Gamma X_{i}^{N}+\eta^{P}\sum_{z=1}^{2} X_{i}^{P_{z}}-\left( u_{i}^{P}+\sum_{V\in\mathbf{V}} \lambda_{i}^{N,V} \right)X_{i}^{N}, \mathbf{V}=\{W,R_{1},C_{1},S_{1},Q_{1}\}$ (4)

$\dot{X}_{i}^{P_{z}}=\Gamma X_{i}^{P_{z}}+u_{i}^{P}\chi_{z}X_{i}^{N}-\left( \eta^{P}+\sum_{V\in\mathbf{V}} \lambda_{i}^{P_{z},V} \right)X_{i}^{P_{z}}, \mathbf{V}=\{W,R_{1},C_{1},S_{1},Q_{1}\}$ (5)

**Compartments of individuals with pre-seroconversion acute HIV infection (*h* = 1)**

$\dot{Y}_{i,1}^{N,V,V}=\Gamma Y_{i,1}^{N,V,V}+\lambda_{i}^{N,V}X_{i}^{N}+\eta^{P}\sum_{z=1}^{2} Y_{i,1}^{P_{z},V,V}-\left( u_{i}^{P}+\gamma_{1,V}+\xi_{V} \right)Y_{i,1}^{N,V,V}$ (6)

$\dot{Y}_{i,1}^{P_{z},V,V}=\Gamma Y_{i,1}^{P_{z},V,V}+\lambda_{i}^{P_{z},V}X_{i}^{P_{z}}+u_{i}^{P}\chi_{z}Y_{i,1}^{N,V,V}-\left( \eta^{P}+\gamma_{1,V}+\zeta_{V}^{P_{z}}+\xi_{V} \right)Y_{i,1}^{P_{z},V,V}$ (7)

$\dot{Y}_{i,1}^{N,v,V}=\Gamma Y_{i,1}^{N,v,V}+\eta^{P}\sum_{z=1}^{2} Y_{i,1}^{P_{z},v,V}-\left( u_{i}^{P}+\gamma_{1,W}+\xi_{V} \right)Y_{i,1}^{N,v,V}$ (8)

$\dot{Y}_{i,1}^{P_{z},v,V}=\Gamma Y_{i,1}^{P_{z},v,V}+u_{i}^{P}\chi_{z}Y_{i,1}^{N,v,V}+\xi_{V}Y_{i,1}^{P_{z},V,V}-\left( \eta^{P}+\gamma_{1,W} \right)Y_{i,1}^{P_{z},v,V}$ (9)

$\dot{Y}_{i,1}^{N,v,v}=\Gamma Y_{i,1}^{N,v,v}+\eta^{P}\sum_{z=1}^{2} Y_{i,1}^{P_{z},v,v}+\xi_{V}\left( Y_{i,1}^{N,V,V}+Y_{i,1}^{N,v,V} \right)-\left( u_{i}^{P}+\gamma_{1,W} \right)Y_{i,1}^{N,v,v}$ (10)

$\dot{Y}_{i,1}^{P_{z},v,v}=\Gamma Y_{i,1}^{P_{z},v,v}+u_{i}^{P}\chi_{z}Y_{i,1}^{N,v,v}+\xi_{V}Y_{i,1}^{P_{z},V,V}-\left( \eta^{P}+\gamma_{1,W}+\zeta_{v}^{P_{z}} \right)Y_{i,1}^{P_{z},v,v}$ (11)

$\dot{Y}_{i,1}^{N,V,Q_{2}}=\Gamma Y_{i,1}^{N,V,Q_{2}}+\eta^{P}\sum_{z=1}^{2} Y_{i,1}^{P_{z},V,Q_{2}}-\left( u_{i}^{P}+\gamma_{1,V}+\xi_{V}+\xi_{Q_{2}} \right)Y_{i,1}^{N,V,Q_{2}}$ (12)

$\dot{Y}_{i,1}^{P_{z},V,Q_{2}}=\Gamma Y_{i,1}^{P_{z},V,Q_{2}}+u_{i}^{P}\chi_{z}\left( Y_{i,1}^{N,V,q_{2}}+Y_{i,1}^{N,V,Q_{2}} \right)+\zeta_{V}^{P_{z}}Y_{i,1}^{P_{z},V,V}-\left( \eta^{P}+\gamma_{1,V}+\xi_{V} \right)Y_{i,1}^{P_{z},V,Q_{2}}$ (13)

$\dot{Y}_{i,1}^{N,v,Q_{2}}=\Gamma Y_{i,1}^{N,v,Q_{2}}+\eta^{P}\sum_{z=1}^{2} Y_{i,1}^{P_{z},v,Q_{2}}+\xi_{V}Y_{i,1}^{N,V,Q_{2}}-\left( u_{i}^{P}+\gamma_{1,W}+\xi_{Q_{2}} \right)Y_{i,1}^{N,v,Q_{2}}$ (14)

$\dot{Y}_{i,1}^{P_{z},v,Q_{2}}=\Gamma Y_{i,1}^{P_{z},v,Q_{2}}+u_{i}^{P}\chi_{z}\left( Y_{i,1}^{N,v,q_{2}}+Y_{i,1}^{N,v,Q_{2}} \right)+\xi_{V}Y_{i,1}^{P_{z},V,Q_{2}}+\zeta_{v}^{P_{z}}Y_{i,1}^{P_{z},v,v}-\left( \eta^{P}+\gamma_{1,W} \right)Y_{i,1}^{P_{z},v,Q_{2}}$ (15)

$\dot{Y}_{i,1}^{N,V,q_{2}}=\Gamma Y_{i,1}^{N,V,q_{2}}+\xi_{Q_{2}}Y_{i,1}^{N,V,Q_{2}}-\left( u_{i}^{P}+\gamma_{1,V}+\xi_{V} \right)Y_{i,1}^{N,V,q_{2}}$ (16)

$\dot{Y}_{i,1}^{N,v,q_{2}}=\Gamma Y_{i,1}^{N,v,q_{2}}+\xi_{V}Y_{i,1}^{N,V,q_{2}}+\xi_{Q_{2}}Y_{i,1}^{N,v,Q_{2}}-\left( u_{i}^{P}+\gamma_{1,W} \right)Y_{i,1}^{N,v,q_{2}}$ (17)

**Compartments of ART-naïve individuals with established HIV infection (*h* > 1)**

$\dot{Y}_{i,h}^{N,V,V}=\Gamma Y_{i,h}^{N,V,V}+\gamma_{h-1,V}Y_{i,h-1}^{N,V,V}+\left( \eta^{P}+\sigma\right)\sum_{z=1}^{2} Y_{i,h}^{P_{z},V,V}+\sum_{z=1}^{3} \eta^{T_{z}}Y_{i,h}^{T_{z},V,V}-\left( u_{h}^{T}+\gamma_{h,V}+\xi_{V} \right)Y_{i,h}^{N,V,V}$ (18)

$\dot{Y}_{i,h}^{P_{z},V,V}=\Gamma Y_{i,h}^{P_{z},V,V}+\gamma_{h-1,V}Y_{i,h-1}^{P_{z},V,V}-\left( \eta^{P}+\sigma+\gamma_{h,V}+\zeta_{V}^{P_{z}}+\xi_{V} \right)Y_{i,h}^{P_{z},V,V}$ (19)

$\dot{Y}_{i,h}^{N,v,V}=\Gamma Y_{i,h}^{N,v,V}+\gamma_{h-1,W}Y_{i,h-1}^{N,v,V}+\left( \eta^{P}+\sigma\right)\sum_{z=1}^{2} Y_{i,h}^{P_{z},v,V}+\sum_{z=1}^{3} \eta^{T_{z}}Y_{i,h}^{T_{z},v,V}-\left( u_{h}^{T}+\gamma_{h,W}+\xi_{V} \right)Y_{i,h}^{N,v,V}$ (20)

$\dot{Y}_{i,h}^{P_{z},v,V}=\Gamma Y_{i,h}^{P_{z},v,V}+\gamma_{h-1,W}Y_{i,h-1}^{P_{z},v,V}+\xi_{V}Y_{i,h}^{P_{z},V,V}-\left( \eta^{P}+\sigma+\gamma_{h,W} \right)Y_{i,h}^{P_{z},v,V}$ (21)

$\dot{Y}_{i,h}^{N,v,v}=\begin{matrix} \Gamma Y_{i,h}^{N,v,v}+\gamma_{h-1,W}Y_{i,h-1}^{N,v,v}+\left( \eta^{P}+\sigma\right)\sum_{z=1}^{2} Y_{i,h}^{P_{z},v,v}+\sum_{z=1}^{3} \eta^{T_{z}}Y_{i,h}^{T_{z},v,v}+\xi_{V}\left( Y_{i,h}^{N,V,V}+Y_{i,h}^{N,v,V} \right) \\ -\left( u_{h}^{T}+\gamma_{h,W} \right)Y_{i,h}^{N,v,v} \end{matrix}$ (22)

$\dot{Y}_{i,h}^{P_{z},v,v}=\Gamma Y_{i,h}^{P_{z},v,v}+\gamma_{h-1,W}Y_{i,h-1}^{P_{z},v,v}+\xi_{V}Y_{i,h}^{P_{z},V,V}-\left( \eta^{P}+\sigma+\gamma_{h,W}+\zeta_{v}^{P_{z}} \right)Y_{i,h}^{P_{z},v,v}$ (23)

$\dot{Y}_{i,h}^{N,V,Q_{2}}=\begin{matrix} \Gamma Y_{i,h}^{N,V,Q_{2}}+\gamma_{h-1,V}Y_{i,h-1}^{N,V,Q_{2}}+\left( \eta^{P}+\sigma\right)\sum_{z=1}^{2} Y_{i,h}^{P_{z},V,Q_{2}}+\sum_{z=1}^{3} \eta^{T_{z}}Y_{i,h}^{T_{z},V,Q_{2}} \\ -\left( u_{h}^{T}+\gamma_{h,V}+\xi_{V}+\xi_{Q_{2}} \right)Y_{i,h}^{N,V,Q_{2}} \end{matrix}$ (24)

$\dot{Y}_{i,h}^{P_{z},V,Q_{2}}=\Gamma Y_{i,h}^{P_{z},V,Q_{2}}+\gamma_{h-1,V}Y_{i,h-1}^{P_{z},V,Q_{2}}+\zeta_{V}^{P_{z}}Y_{i,h}^{P_{z},V,V}-\left( \eta^{P}+\sigma+\gamma_{h,V}+\xi_{V} \right)Y_{i,h}^{P_{z},V,Q_{2}}$ (25)

$\dot{Y}_{i,h}^{N,v,Q_{2}}=\begin{matrix} \Gamma Y_{i,h}^{N,v,Q_{2}}+\gamma_{h-1,W}Y_{i,h-1}^{N,v,Q_{2}}+\left( \eta^{P}+\sigma\right)\sum_{z=1}^{2} Y_{i,h}^{P_{z},v,Q_{2}}+\sum_{z=1}^{3} \eta^{T_{z}}Y_{i,h}^{T_{z},v,Q_{2}}+\xi_{V}Y_{i,h}^{N,V,Q_{2}} \\ -\left( u_{h}^{T}+\gamma_{h,W}+\xi_{Q_{2}} \right)Y_{i,h}^{N,v,Q_{2}} \end{matrix}$ (26)

$\dot{Y}_{i,h}^{P_{z},v,Q_{2}}=\Gamma Y_{i,h}^{P_{z},v,Q_{2}}+\gamma_{h-1,W}Y_{i,h-1}^{P_{z},v,Q_{2}}+\xi_{V}Y_{i,h}^{P_{z},V,Q_{2}}+\zeta_{v}^{P_{z}}Y_{i,h}^{P_{z},v,v}-\left( \eta^{P}+\sigma+\gamma_{h,W} \right)Y_{i,h}^{P_{z},v,Q_{2}}$ (27)

$\dot{Y}_{i,h}^{N,V,q_{2}}=\Gamma Y_{i,h}^{N,V,q_{2}}+\gamma_{h-1,V}Y_{i,h-1}^{N,V,q_{2}}+\xi_{Q_{2}}Y_{i,h}^{N,V,Q_{2}}+\sum_{z=1}^{3} \eta^{T_{z}}Y_{i,h}^{T_{z},V,q_{2}}-\left( u_{h}^{T}+\gamma_{h,V}+\xi_{V} \right)Y_{i,h}^{N,V,q_{2}}$ (28)

$\dot{Y}_{i,h}^{N,v,q_{2}}=\Gamma Y_{i,h}^{N,v,q_{2}}+\gamma_{h-1,W}Y_{i,h-1}^{N,v,q_{2}}+\xi_{V}Y_{i,h}^{N,V,q_{2}}+\xi_{Q_{2}}Y_{i,h}^{N,v,Q_{2}}+\sum_{z=1}^{3} \eta^{T_{z}}Y_{i,h}^{T_{z},v,q_{2}}-\left( u_{h}^{T}+\gamma_{h,W} \right)Y_{i,h}^{N,v,q_{2}}$ (29)

**Compartments of individuals on first-line ART (*h* > 1)**

$\dot{Y}_{i,h}^{T_{1},V,V^{'}}=\Gamma Y_{i,h}^{T_{1},V,V^{'}}+u_{h}^{T}Y_{i,h}^{N,V,V^{'}}-\left( \omega_{V}^{T_{1}}+\eta^{T_{1}}+1+\mu_{h}^{T_{1}} \right)Y_{i,h}^{T_{1},V,V^{'}}$ (30)

$\dot{Y}_{i,h}^{T_{2},V,V^{'}}=\Gamma Y_{i,h}^{T_{2},V,V^{'}}+Y_{i,h}^{T_{1},V,V^{'}}-\left( \omega_{V}^{T_{2}}+\eta^{T_{2}}+\mu_{h}^{T_{2}} \right)Y_{i,h}^{T_{2},V,V^{'}}$ (31)

$\dot{Y}_{i,h}^{T_{3},V,V^{'}}=\begin{matrix} \Gamma Y_{i,h}^{T_{3},V,V^{'}}+\gamma_{h-1,V}Y_{i,h-1}^{T_{3},V,V^{'}}+\sum_{z=1}^{2} \omega_{V}^{T_{z}}\left( 1-\psi_{V}^{T_{z}} \right)Y_{i,h}^{T_{z},V,V^{'}} \\ -\left( \eta^{T_{3}}+\gamma_{h,V}+\xi_{V}+\xi_{V^{'}}+u^{Z} \right)Y_{i,h}^{T_{3},V,V^{'}} \end{matrix}$ (32)

$\dot{Y}_{i,h}^{T_{1},v,V^{'}}=\Gamma Y_{i,h}^{T_{1},v,V^{'}}+u_{h}^{T}Y_{i,h}^{N,v,V^{'}}-\left( \omega_{v}^{T_{1}}+\eta^{T_{1}}+1+\mu_{h}^{T_{1}} \right)Y_{i,h}^{T_{1},v,V^{'}}$ (33)

$\dot{Y}_{i,h}^{T_{2},v,V^{'}}=\Gamma Y_{i,h}^{T_{2},v,V^{'}}+Y_{i,h}^{T_{1},v,V^{'}}-\left( \omega_{v}^{T_{2}}+\eta^{T_{2}}+\mu_{h}^{T_{2}} \right)Y_{i,h}^{T_{2},v,V^{'}}$ (34)

$\dot{Y}_{i,h}^{T_{3},v,V^{'}}=\begin{matrix} \Gamma Y_{i,h}^{T_{3},v,V^{'}}+\gamma_{h-1,W}Y_{i,h-1}^{T_{3},v,V^{'}}+\sum_{z=1}^{2} \omega_{v}^{T_{z}}\left( 1-\psi_{v}^{T_{z}} \right)Y_{i,h}^{T_{z},v,V^{'}}+\xi_{V} \\ -\left( \eta^{T_{3}}+\gamma_{h,W}+\xi_{V^{'}}+u^{Z} \right)Y_{i,h}^{T_{3},v,V^{'}} \end{matrix}Y_{i,h}^{T_{3},V,V^{'}}$ (35)

$\dot{Y}_{i,h}^{T_{1},V,v^{'}}=\Gamma Y_{i,h}^{T_{1},V,v^{'}}+u_{h}^{T}Y_{i,h}^{N,V,v^{'}}-\left( \omega_{V}^{T_{1}}+\eta^{T_{1}}+1+\mu_{h}^{T_{1}} \right)Y_{i,h}^{T_{1},V,v^{'}}$ (36)

$\dot{Y}_{i,h}^{T_{2},V,v^{'}}=\Gamma Y_{i,h}^{T_{2},V,v^{'}}+Y_{i,h}^{T_{1},V,v^{'}}-\left( \omega_{V}^{T_{2}}+\eta^{T_{2}}+\mu_{h}^{T_{2}} \right)Y_{i,h}^{T_{2},V,v^{'}}$ (37)

$\dot{Y}_{i,h}^{T_{3},V,v^{'}}=\begin{matrix} \Gamma Y_{i,h}^{T_{3},V,v^{'}}+\gamma_{h-1,V}Y_{i,h-1}^{T_{3},V,v^{'}}+\sum_{z=1}^{2} \omega_{V}^{T_{z}}\left( 1-\psi_{V}^{T_{z}} \right)Y_{i,h}^{T_{z},V,v^{'}}+\xi_{V^{'}} \\ -\left( \eta^{T_{3}}+\gamma_{h,V}+\xi_{V}+u^{Z} \right)Y_{i,h}^{T_{3},V,v^{'}} \end{matrix}Y_{i,h}^{T_{3},V,V^{'}}$ (38)

$\dot{Y}_{i,h}^{T_{1},v,v^{'}}=\Gamma Y_{i,h}^{T_{1},v,v^{'}}+u_{h}^{T}Y_{i,h}^{N,v,v^{'}}-\left( \omega_{v}^{T_{1}}+\eta^{T_{1}}+1+\mu_{h}^{T_{1}} \right)Y_{i,h}^{T_{1},v,v^{'}}$ (39)

$\dot{Y}_{i,h}^{T_{2},v,v^{'}}=\Gamma Y_{i,h}^{T_{2},v,v^{'}}+Y_{i,h}^{T_{1},v,v^{'}}-\left( \omega_{v}^{T_{2}}+\eta^{T_{2}}+\mu_{h}^{T_{2}} \right)Y_{i,h}^{T_{2},v,v^{'}}$ (40)

$\dot{Y}_{i,h}^{T_{3},v,v^{'}}=\begin{matrix} \Gamma Y_{i,h}^{T_{3},v,v^{'}}+\gamma_{h-1,W}Y_{i,h-1}^{T_{3},v,v^{'}}+\sum_{z=1}^{2} \omega_{v}^{T_{z}}\left( 1-\psi_{v}^{T_{z}} \right)Y_{i,h}^{T_{z},v,v^{'}}+\xi_{V^{'}} \\ -\left( \eta^{T_{3}}+\gamma_{h,W}+u^{Z} \right)Y_{i,h}^{T_{3},v,v^{'}} \end{matrix}Y_{i,h}^{T_{3},v,V^{'}}+\xi_{V}Y_{i,h}^{T_{3},V,v^{'}}$ (41)

$\dot{Y}_{i,h}^{T_{2},R_{2},R_{2}}=\begin{matrix} \Gamma Y_{i,h}^{T_{2},R_{2},R_{2}}+\gamma_{h-1,R_{2}}Y_{i,h-1}^{T_{2},R_{2},R_{2}}+u_{h}^{T}\left( Y_{i,h}^{N,R_{2},R_{2}}+Y_{i,h}^{N,r_{2},r_{2}} \right)-\left( \eta^{T_{2}}+\gamma_{h,R_{2}}+u^{Z} \right)Y_{i,h}^{T_{2},R_{2},R_{2}} \\ +\sum_{u\in\mathbf{V}} \sum_{u^{'}\in\mathbf{V}} \sum_{z=1}^{2} \omega_{u}^{T_{z}}\psi_{u}^{T_{z}}\left( 1-x_{u,u^{'}} \right)Y_{i,h}^{T_{z},u,u^{'}}, \mathbf{V}=\{W,R_{1},C_{1},{S_{1},Q}_{1},Q_{2},r_{1},c_{1},s_{1},q_{1},q_{2}\} \end{matrix}$ (42)

$\dot{Y}_{i,h}^{N,R_{2},R_{2}}=\Gamma Y_{i,h}^{N,R_{2},R_{2}}+\gamma_{h-1,R_{2}}Y_{i,h-1}^{N,R_{2},R_{2}}+\eta^{T_{2}}Y_{i,h}^{T_{2},R_{2},R_{2}}-\left( u_{h}^{T}+\gamma_{h,R_{2}}+\xi_{R_{2}} \right)Y_{i,h}^{N,R_{2},R_{2}}$ (43)

$\dot{Y}_{i,h}^{N,r_{2},r_{2}}=\Gamma Y_{i,h}^{N,r_{2},r_{2}}+\gamma_{h-1,W}Y_{i,h-1}^{N,r_{2},r_{2}}+\xi_{R_{2}}Y_{i,h}^{N,R_{2},R_{2}}-\left( u_{h}^{T}+\gamma_{h,W} \right)Y_{i,h}^{N,r_{2},r_{2}}$ (44)

$\dot{Y}_{i,h}^{T_{2},C_{2},C_{2}}=\begin{matrix} \Gamma Y_{i,h}^{T_{2},C_{2},C_{2}}+\gamma_{h-1,C_{2}}Y_{i,h-1}^{T_{2},C_{2},C_{2}}+u_{h}^{T}\left( Y_{i,h}^{N,C_{2},C_{2}}+Y_{i,h}^{N,c_{2},c_{2}} \right)-\left( \eta^{T_{2}}+\gamma_{h,C_{2}}+u^{Z} \right)Y_{i,h}^{T_{2},C_{2},C_{2}} \\ +\sum_{u\in\mathbf{V}} \sum_{u^{'}\in\mathbf{V}} \sum_{z=1}^{2} \omega_{u}^{T_{z}}\psi_{u}^{T_{z}}x_{u,u^{'}}Y_{i,h}^{T_{z},u,u^{'}}, \mathbf{V}=\{W,R_{1},C_{1},{S_{1},Q}_{1},Q_{2},r_{1},c_{1},s_{1},q_{1},q_{2}\} \end{matrix}$ (45)

$\dot{Y}_{i,h}^{N,C_{2},C_{2}}=\Gamma Y_{i,h}^{N,C_{2},C_{2}}+\gamma_{h-1,C_{2}}Y_{i,h-1}^{N,C_{2},C_{2}}+\eta^{T_{2}}Y_{i,h}^{T_{2},C_{2},C_{2}}-\left( u_{h}^{T}+\gamma_{h,C_{2}}+\xi_{C_{2}} \right)Y_{i,h}^{N,C_{2},C_{2}}$ (46)

$\dot{Y}_{i,h}^{N,c_{2},c_{2}}=\Gamma Y_{i,h}^{N,c_{2},c_{2}}+\gamma_{h-1,W}Y_{i,h-1}^{N,c_{2},c_{2}}+\xi_{C_{2}}Y_{i,h}^{N,C_{2},C_{2}}-\left( u_{h}^{T}+\gamma_{h,W} \right)Y_{i,h}^{N,c_{2},c_{2}}$ (47)

**Compartments of individuals on second-line ART (*h* > 1)**

$\dot{Z}_{i,h}^{T_{2},V,V^{'}}=\Gamma Z_{i,h}^{T_{2},V,V^{'}}+u_{h}^{T}Z_{i,h}^{N,V,V^{'}}-\left( \omega_{V}^{Z}+\eta^{Z}+\mu_{h}^{Z}+\xi_{V}+\xi_{V^{'}} \right)Z_{i,h}^{T_{2},V,V^{'}}+u^{Z}\left\{ \begin{matrix} Y_{i,h}^{T_{2},V,V^{'}}, & V\in\{R_{2},C_{2}\} \\ Y_{i,h}^{T_{3},V,V^{'}}, & V\notin\{R_{2},C_{2}\} \end{matrix} \right.$ (48)

$\dot{Z}_{i,h}^{T_{3},V,V^{'}}=\Gamma Z_{i,h}^{T_{3},V,V^{'}}+\gamma_{h-1,V}Z_{i,h-1}^{T_{3},V,V^{'}}+\omega_{V}^{Z}\left( 1-\psi_{V}^{Z} \right)Z_{i,h}^{T_{2},V,V^{'}}-\left( \eta^{Z}+\gamma_{h,V}+\xi_{V}+\xi_{V^{'}} \right)Z_{i,h}^{T_{3},V,V^{'}}$ (49)

$\dot{Z}_{i,h}^{N,V,V^{'}}=\Gamma Z_{i,h}^{N,V,V^{'}}+\gamma_{h-1,V}Z_{i,h-1}^{N,V,V^{'}}+\eta^{Z}\sum_{z=2}^{3} Z_{i,h}^{T_{z},V,V^{'}}-\left( \gamma_{h,V}+\xi_{V}+\xi_{V^{'}}+u_{h}^{T} \right)Z_{i,h}^{N,V,V^{'}}$ (50)

$\dot{Z}_{i,h}^{T_{2},v,V^{'}}=\Gamma Z_{i,h}^{T_{2},v,V^{'}}+u_{h}^{T}Z_{i,h}^{N,v,V^{'}}+\xi_{V}Z_{i,h}^{T_{2},V,V^{'}}-\left( \omega_{v}^{Z}+\eta^{Z}+\mu_{h}^{Z}+\xi_{V^{'}} \right)Z_{i,h}^{T_{2},v,V^{'}}+u^{Z}Y_{i,h}^{T_{3},v,V^{'}}$ (51)

$\dot{Z}_{i,h}^{T_{3},v,V^{'}}=\Gamma Z_{i,h}^{T_{3},v,V^{'}}+\gamma_{h-1,W}Z_{i,h-1}^{T_{3},v,V^{'}}+\xi_{V}Z_{i,h}^{T_{3},V,V^{'}}+\omega_{v}^{Z}\left( 1-\psi_{v}^{Z} \right)Z_{i,h}^{T_{2},v,V^{'}}-\left( \eta^{Z}+\gamma_{h,W}+\xi_{V^{'}} \right)Z_{i,h}^{T_{3},v,V^{'}}$ (52)

$\dot{Z}_{i,h}^{N,v,V^{'}}=\Gamma Z_{i,h}^{N,v,V^{'}}+\gamma_{h-1,W}Z_{i,h-1}^{N,v,V^{'}}+\xi_{V}Z_{i,h}^{N,V,V^{'}}+\eta^{Z}\sum_{z=2}^{3} Z_{i,h}^{T_{z},v,V^{'}}-\left( \gamma_{h,W}+\xi_{V^{'}}+u_{h}^{T} \right)Z_{i,h}^{N,v,V^{'}}$ (53)

$\dot{Z}_{i,h}^{T_{2},V,v^{'}}=\Gamma Z_{i,h}^{T_{2},V,v^{'}}+u_{h}^{T}Z_{i,h}^{N,V,v^{'}}+\xi_{V^{'}}Z_{i,h}^{T_{2},V,V^{'}}-\left( \omega_{V}^{Z}+\eta^{Z}+\mu_{h}^{Z}+\xi_{V} \right)Z_{i,h}^{T_{2},V,v^{'}}+u^{Z}Y_{i,h}^{T_{3},v,V^{'}}$ (54)

$\dot{Z}_{i,h}^{T_{3},V,v^{'}}=\Gamma Z_{i,h}^{T_{3},V,v^{'}}+\gamma_{h-1,V}Z_{i,h-1}^{T_{3},V,v^{'}}+\xi_{V^{'}}Z_{i,h}^{T_{3},V,V^{'}}+\omega_{V}^{Z}\left( 1-\psi_{V}^{Z} \right)Z_{i,h}^{T_{2},V,v^{'}}-\left( \eta^{Z}+\gamma_{h,V}+\xi_{V} \right)Z_{i,h}^{T_{3},V,v^{'}}$ (55)

$\dot{Z}_{i,h}^{N,V,v^{'}}=\Gamma Z_{i,h}^{N,V,v^{'}}+\gamma_{h-1,V}Z_{i,h-1}^{N,V,v^{'}}+\xi_{V^{'}}Z_{i,h}^{N,V,V^{'}}+\eta^{Z}\sum_{z=2}^{3} Z_{i,h}^{T_{z},V,v^{'}}-\left( \gamma_{h,V}+\xi_{V}+u_{h}^{T} \right)Z_{i,h}^{N,V,v^{'}}$ (56)

$\dot{Z}_{i,h}^{T_{2},v,v^{'}}=\Gamma Z_{i,h}^{T_{2},v,v^{'}}+u_{h}^{T}Z_{i,h}^{N,v,v^{'}}+\xi_{V}Z_{i,h}^{T_{2},V,v^{'}}+\xi_{V^{'}}Z_{i,h}^{T_{2},v,V^{'}}-\left( \omega_{v}^{Z}+\eta^{Z}+\mu_{h}^{Z} \right)Z_{i,h}^{T_{2},v,v^{'}}+u^{Z}Y_{i,h}^{T_{3},v,v^{'}}$ (57)

$\dot{Z}_{i,h}^{T_{3},v,v^{'}}=\Gamma Z_{i,h}^{T_{3},v,v^{'}}+\gamma_{h-1,W}Z_{i,h-1}^{T_{3},v,v^{'}}+\xi_{V}Z_{i,h}^{T_{3},V,v^{'}}+\xi_{V^{'}}^{N}Z_{i,h}^{T_{3},v,V^{'}}+\omega_{v}^{Z}\left( 1-\psi_{v}^{Z} \right)Z_{i,h}^{T_{2},v,v^{'}}-\left( \eta^{Z}+\gamma_{h,W} \right)Z_{i,h}^{T_{3},v,v^{'}}$ (58)

$\dot{Z}_{i,h}^{N,v,v^{'}}=\Gamma Z_{i,h}^{N,v,v^{'}}+\gamma_{h-1,W}Z_{i,h-1}^{N,v,v^{'}}+\xi_{V}Z_{i,h}^{N,V,v^{'}}+\xi_{V^{'}}Z_{i,h}^{N,v,V^{'}}+\eta^{Z}\sum_{z=2}^{3} Z_{i,h}^{T_{z},v,v^{'}}-\left( \gamma_{h,W}+u_{h}^{T} \right)Z_{i,h}^{N,v,v^{'}}$ (59)

$\dot{Z}_{i,h}^{T_{2},S_{2},S_{2}}=\begin{matrix} \Gamma Z_{i,h}^{T_{2},S_{2},S_{2}}+\gamma_{h-1,S_{2}}Z_{i,h-1}^{T_{2},S_{2},S_{2}}+u_{h}^{T}\left( Z_{i,h}^{N,S_{2},S_{2}}+Z_{i,h}^{N,s_{2},s_{2}} \right)-\left( \gamma_{h,S_{2}}+\eta^{Z} \right)Z_{i,h}^{T_{2},S_{2},S_{2}} \\ +\sum_{u\in\mathbf{V}} \sum_{u^{'}\in\mathbf{V}} \omega_{u}^{Z}\psi_{u}^{Z}Z_{i,h}^{T_{2},S_{2},S_{2}}, \mathbf{V}=\{W,R_{1},C_{1},{S_{1},Q}_{1},R_{2},C_{2},Q_{2},r_{1},c_{1},s_{1},q_{1},r_{2},c_{2},q_{2}\} \end{matrix}$ (60)

$\dot{Z}_{i,h}^{N,S_{2},S_{2}}=\Gamma Z_{i,h}^{N,S_{2},S_{2}}+\gamma_{h-1,S_{2}}Z_{i,h-1}^{N,S_{2},S_{2}}+\eta^{Z}Z_{i,h}^{T_{2},S_{2},S_{2}}-\left( \gamma_{h,S_{2}}+\xi_{S_{2}}+u_{h}^{T} \right)Z_{i,h}^{N,S_{2},S_{2}}$ (61)

$\dot{Z}_{i,h}^{N,s_{2},s_{2}}=\Gamma Z_{i,h}^{N,s_{2},s_{2}}+\gamma_{h-1,W}Z_{i,h-1}^{N,s_{2},s_{2}}+\xi_{S_{2}}Z_{i,h}^{N,S_{2},S_{2}}-\left( \gamma_{h,W}+u_{h}^{T} \right)Z_{i,h}^{N,s_{2},s_{2}}$ (62)

## HIV transmission

### Force of infection

Our model represents heterosexual transmission of five different HIV variants: drug-sensitive wild-type ($V=W$), transmitted resistance to first-line ART with ($C_{1}$) or without ($R_{1}$) cross-resistance to DPV, transmitted resistance to second-line ART ($S_{1}$), or transmitted resistance to PrEP ($Q_{1}$). When calculating the force of infection, we aggregate sexually active individuals into five-year age bands (15–19, 20–24, …, 50–54) [1, 2], and aggregate HIV-positive male donors by VMMC status, as VMMC has no effect on an HIV-positive male’s probability of HIV transmission [53]. The force of infection $\lambda_{i}^{z,V}$ of HIV variant $V$ acting on HIV-negative individuals in risk group $i=(g,a,k,j)$ (consisting of sex *g*, five-year age band *a*, sexual activity level *k*, and VMMC status *j*) having PrEP status $z$ is

$\lambda_{i}^{z,V}=c_{i}\sum_{i^{'}} \left( m_{i,i^{'}}\Delta_{i,i^{'}}/N_{i^{'}} \right)\sum_{h=1}^{6} \sum_{v\in\mathbf{V}_{V}} \sum_{v^{'}} \sum_{z^{'}} \left( Y_{i^{'},h}^{z^{'},v^{'},v}+Z_{i^{'},h}^{z^{'},v^{'},v} \right)\beta_{i,i^{'},h}^{z,z^{'},v}$ , (63)

where $c_{i}$ is the rate that individuals in risk group *i* change sexual partners, $m_{i,i^{'}}$ is the mixing coefficient between risk groups *i* and $i'$, $\Delta_{i,i^{'}}$ adjusts for imbalances in partnership supply and demand between risk groups, $N_{i^{'}}$ is the number of individuals in risk group $i'$, $\mathbf{V}_{V}$ is the set of donor HIV variants that transmit variant $V$ ($\mathbf{V}_{W}=\{W,r_{1},c_{1},q_{1},s_{1},r_{2},c_{2},q_{2},s_{2}\}$, $\mathbf{V}_{R_{1}}=\{R_{1},R_{2}\}$, $\mathbf{V}_{C_{1}}=\{C_{1},C_{2}\}$, $\mathbf{V}_{S_{1}}=\left\{ S_{1},S_{2} \right\}$, and $\mathbf{V}_{Q_{1}}=\{Q_{1},Q_{2}\}$), and $\beta_{i,i^{'},h}^{z,z^{'},v}$ is the per-partnership risk of HIV transmission from an HIV-positive donor in risk group *i'* and disease stage *h*, having ARV status $z^{'}$ and genital tract HIV variant *v*, to a recipient in risk group *i* who has PrEP status $z$. We assume individuals with AIDS reduce their partner change rates by 35% due to poor health [12]. The remaining terms of the force of infection are described in detail below.

### Mixing and balancing

Sexual mixing in our model is a hybrid of random mixing and preferential mixing by sexual activity level and/or age [55, 56]. Age-based preferential mixing occurs between men and women in the same five-year age band (*e.g.*, 20–24 year-old men and women) or men who are five years older than their partners (*e.g*., 20–24 year-old men and 15–19 year-old women). Let $i=(g,a,k)$ and $i^{'}=(g^{'},a^{'},k^{'})$ be the respective genders, age band, and sexual activity levels of individuals in risk groups *i* and $i^{'}$ (we assume VMMC status does not influence partner preferences, and aggregate males by VMMC status when calculating mixing and balancing terms). The mixing coefficients $m_{i,i^{'}}$ quantify the extent of preferential versus random mixing. We model heterosexual mixing only ($m_{i,i^{'}}=0$ if $g=g^{'}$). Mixing coefficients between risk group *i* of men and risk group $i^{'}$ of women are defined as follows:

$m_{i,i^{'}}=m_{\left( g,a,k \right),\left( g^{'},a^{'},k^{'} \right)}=\left\{ \begin{matrix} \left( 1-\alpha_{D} \right)m_{i,i^{'}}^{A}m_{i,i^{'}}^{K}, & a>1 \mathrm{and} a=a^{'}, \\ \left( m_{i,i^{'}}^{A}+\alpha^{D}m_{i,\left( g^{'},a^{'}+1,k^{'} \right)}^{A} \right)m_{i,j}^{K}, & a>1 \mathrm{and} a=a^{'}+1, \\ m_{i,i^{'}}^{A}m_{i,i^{'}}^{K}, & otherwise. \end{matrix} \right.$ (64)

Age bands in Equation 64 have indices $a=1$ for ages 15–19, $a=2$ for ages 20–24, continuing up to $a=8$ for ages 50–54. Mixing coefficients for women are defined analogously, except that women mix preferentially with men who are five years older whereas men mix preferentially with younger women. The parameter $\alpha^{D}$ quantifies the degree of preferential mixing between older men and younger women. The terms $m_{i,i^{'}}^{A}$ and $m_{i,i^{'}}^{K}$ factor the mixing coefficient $m_{i,i^{'}}$ according to preferential mixing by age and by sexual activity level, respectively:

$m_{i,i^{'}}^{A}=\left( 1-\alpha^{A} \right)\frac{\sum_{l=1}^{4} c_{\left( g^{'},a^{'},l \right)}N_{\left( g^{'},a^{'},l \right)}}{\sum_{l=1}^{4} \sum_{b=1}^{8} c_{\left( g^{'},b,l \right)}N_{\left( g^{'},b,l \right)}}+\alpha^{A}\delta_{a,a^{'}}$ (65)

$m_{i,i^{'}}^{K}=\left( 1-\alpha^{K} \right)\frac{c_{\left( g^{'},a^{'},k^{'} \right)}N_{\left( g^{'},a^{'},k^{'} \right)}}{\sum_{l=1}^{4} c_{\left( g^{'},a^{'},l \right)}N_{\left( g^{'},a^{'},l \right)}}+\alpha^{K}\delta_{k,k^{'}}$ (66)

The terms $\alpha^{A}$ and $\alpha^{K}$ quantify the extent of preferential mixing by (same) age and sexual activity level, respectively. $\delta_{a,a^{'}}$ is the Kronecker delta ($\delta_{a,a^{'}}=1$ if $a=a^{'}$, $\delta_{a,a^{'}}=0$ otherwise).

Supply and demand for partnerships between men and women may become unbalanced due to behavioral change and differential HIV mortality. For each pair of risk groups *i* and *i'*, the balancing terms $\Delta_{i,i^{'}}=\sqrt{({c_{i^{'}}m_{i^{'},i}N_{i^{'}})}/{{(c}_{i}m_{i,i^{'}}N_{i})}}$ dynamically adjust partner change rates to match supply to demand [55, 56],

$c_{i}m_{i,i^{'}}\Delta_{i,i^{'}}N_{i}=c_{i^{'}}m_{i^{'},i}\Delta_{i^{'},i}N_{i^{'}}.$ (67)

### HIV transmission within partnerships

HIV transmission within serodiscordant partnerships in our model depends on each partner’s behavioral risk, including condom use, the HIV-negative recipient’s VMMC status if male or PrEP status if female, and the HIV-positive donor’s disease stage, HIV variant, and ARV use. We partition sexual acts into four categories according to PrEP and/or condom use:

1. DPV ring not worn, condom not used,
2. DPV ring not worn, condom used,
3. DPV ring worn, condom not used,
4. DPV ring worn, condom used,

We use a Bernoulli model to define $\beta_{i,i^{'},h}^{z,z^{'},v}$, the probability of HIV transmission from a donor in risk group $i^{'}$, disease stage *h*, ARV status $z^{'}$, and harboring HIV variant $v$ to a recipient in risk group *i* with PrEP status $z$:

$\beta_{i,i^{'},h}^{z,z^{'},v}=1-\prod_{l=1}^{4} \left( 1-\rho_{i,l}^{v}\tilde{\beta}_{h}^{z^{'},v} \right)^{n_{i,i^{'}}\pi_{l,i,i^{'}}^{z}}$, (68)

where $l$ is the PrEP and condom use category of sexual acts, $\rho_{i,l}^{v}$ is the recipient’s relative risk of HIV acquisition, $\tilde{\beta}_{h}^{z^{'},v}$ is the donor’s transmission probability per sexual act, and $n_{i,i^{'}}$ is the number of sexual acts per partnership. The proportion of sexual acts in category *l*, $\pi_{l,i,i^{'}}^{z}$, depends on whether or not the recipient is on PrEP:

*Recipients not on PrEP*. In this case, sexual acts fall into either category 1 or 2, depending on whether a condom is used (proportion $\pi_{i,i^{'}}^{C}$, defined in Section 3.3.1):

$\pi_{l,i,i^{'}}^{N}=\left\{ \begin{matrix} 1-\pi_{i,i^{'}}^{C}, & l=1 \\ \pi_{i,i^{'}}^{C}, & l=2 \\ 0, & otherwise. \end{matrix} \right.$ (69)

*Recipients on PrEP*. In this case, sexual acts may fall into any category, depending on whether a condom is used (proportion $\pi_{i,i^{'}}^{C}$) or the DPV vaginal ring is worn (proportion $\kappa_{z}$ for recipients in PrEP adherence stratum $P_{z}$):

$\pi_{l,i,j}^{P_{z}}=\left\{ \begin{matrix} (1-\kappa_{z})\left( 1-\pi_{i,j}^{C} \right), & l=1 \\ \left( 1-\kappa_{z} \right)\pi_{i,j}^{C}, & l=2 \\ \kappa_{z}\left( 1-\pi_{i,j}^{C} \right), & l=3 \\ \kappa_{z}\pi_{i,j}^{C}, & l=4 \end{matrix} \right.$ (70)

A recipient’s relative risk of HIV acquisition during a sexual act ($\rho_{i,l}^{v})$is reduced by condom use (efficacy $\varepsilon^{C}$), PrEP use if female (efficacy $\varepsilon_{v}^{P}$), and VMMC status if male (efficacy $\varepsilon^{M}$). PrEP efficacy may be reduced if the donor’s genital HIV variant $v$ is PrEP-resistant or cross-resistant. We assume the overall efficacy of PrEP, condoms, and VMMC is the product of their individual efficacies:

$\rho_{i,l}^{v}=\left\{ \begin{matrix} 1-\varepsilon^{M}, & circumcised male recipient \\ 1, & \mathrm{otherwise} \end{matrix} \right\}\times\left\{ \begin{matrix} 1-\varepsilon^{C}, & l\in\{2,4\} \\ 1, & \mathrm{otherwise} \end{matrix} \right\}\times\left\{ \begin{matrix} 1-\varepsilon_{v}^{P}, & l\in\{3,4\} \\ 1, & \mathrm{otherwise} \end{matrix} \right\}$ (71)

A donor’s risk of transmitting HIV during a given sexual act ($\tilde{\beta}_{h}^{z^{'},v})$depends on his or her ARV status $z^{'}$, disease stage $h$, and genital tract HIV variant $v$:

$\tilde{\beta}_{h}^{z^{'},v}=\left\{ \begin{matrix} 1-\varepsilon^{T}, & virologically suppressed on ART \\ 1-{\kappa_{z}\varepsilon}_{v}^{S}, & acute infection, on PrEP \left( adherence stratum P_{z} \right) \\ 1, & \mathrm{otherwise} \end{matrix} \right\}\times\tilde{\beta}_{h}^{N,v}$ (72)

A donor who is adherent to first- or second-line ART and who has not acquired resistance to his or her current regimen is considered virologically suppressed. In sensitivity analysis, HIV-positive women may have a reduced risk of HIV transmission during acute infection while on PrEP (efficacy $\varepsilon_{v}^{S}$, assumed $\varepsilon_{v}^{S}=0$ in base-case analyses) due to reduced viral load in the genital tract [32, 38].

## Intervention implementation

### Condom use

The proportion of sexual acts in which condoms are used ($\pi_{i,i^{'}}^{C}$ for partners in risk groups $i$ and $i^{'}$), depends on the sexual activity level of each participant in a sexual partnership. We model increases in condom use over time [5, 7, 57] and define the level of condom use at time $t$ as

$\pi_{i,i^{'}}^{C}\left( t \right)=\pi_{i,i^{'}}^{C}\left( t_{0} \right)+\left[ 1-\pi_{i,i^{'}}^{C}\left( t_{0} \right) \right]\left[ 1-p^{C} \right]\times\left\{ \begin{matrix} 0, & t\leq t_{1}^{C} \\ \frac{t-t_{1}^{C}}{t_{2}^{C}-t_{1}^{C}}, & t_{1}^{C}<t\leq t_{2}^{C} \\ 1, & t>t_{2}^{C}. \end{matrix} \right.$ (73)

Condom use begins to increase from initial levels ($\pi_{i,i^{'}}^{C}\left( t_{0} \right)$) at $t_{1}^{C}=1999.3$ and stabilizes at $t_{2}^{C}=2006.5$. During this interval the proportion of sexual acts without condom use decreases by 45% ($p^{C}=0.55$) [2] (Supplementary Table 1).

### Voluntary medical male circumcision

We simulate VMMC scale-up by increasing the proportion of males who are circumcised prior to sexual debut (proportion $U_{2,2}^{M}\left( t \right)$). Scale-up is supplemented by VMMC uptake among sexually active men (rate $u^{M}\left( t \right)$) during 2011–2021, as described elsewhere [2]. For clarity, flows for circumcision uptake are omitted in Equations 4–62. For HIV-negative men these flows consist of an outflow $-u^{M}\left( t \right)X_{2,a,k}^{1,N}$ from each compartment of uncircumcised men, paired with inflow $+u^{M}\left( t \right)X_{2,a,k}^{2,N}$ to the corresponding compartment of circumcised men. Circumcision uptake flows for compartments of HIV-positive men are defined analogously.

### Antiretroviral therapy

The model represents temporal changes in ART availability and eligibility. Per-capita ART uptake rates are calculated as $u_{h}^{T}\left( t \right)= -log(1-U_{h}^{T}\left( t \right))$, where $U_{h}^{T}\left( t \right)$ is the proportion of HIV-positive individuals in disease stage $h$ who initiate ART at time $t$:

$U_{h}^{T}(t)=\left\{ \begin{matrix} 0, & t<t_{h,1}^{T} \\ p_{h,1}^{T}\frac{t-t_{h,1}^{T}}{t_{h,m}^{T}-t_{h,1}^{T}}, & t_{h,1}^{T}\leq t<t_{h,m}^{T} \\ p_{h,1}^{T}+(p_{h,2}^{T}-p_{h,1}^{T})\frac{t-t_{h,m}^{T}}{t_{h,2}^{T}-t_{h,m}^{T}}, & t_{h,m}^{T}\leq t<t_{h,2}^{T} \\ p_{h,2}^{T}, & t\geq t_{h,2}^{T}. \end{matrix} \right.$ (74)

Individuals in disease stage $h$ become eligible for ART at time $t_{h,1}^{T}$. Stage-specific uptake rates peak at time $t_{h,m}^{T}=(t_{h,1}^{T}+t_{h,2}^{T})/2$, then remain constant from time $t_{h,2}^{T}$ onward. Coverage among HIV-positive individuals with CD4 ≤ 200 cells/µL (stage $h=6$) begins at the start of 2004 and scales up through the start of 2012; coverage of individuals with 201–350 CD4 cells/µL ($h=5$) begins at 2010 and scales up through 2017 [48, 58], while coverage of individuals with 351–500 CD4 cells/µL ($h=4$) begins at 2015 and scales up through 2021 [39]. The parameters $p_{h,\bullet}^{T}$ are fit to trends in ART coverage in KwaZulu-Natal [42], and so that ~80% coverage is reached among HIV-positive individuals i) with CD4 ≤ 200 cells/µL at 2012, ii) with CD4 ≤ 350 cells/µL at 2017, and iii) with CD4 ≤ 500 cells/µL from 2021 onward. To simulate achievement of Fast-Track treatment targets, we include scale-up of routine HIV testing to increase treatment coverage, and an adherence support intervention to improve virologic suppression on ART. Both interventions are introduced at September 1, 2016 and scale-up linearly thereafter. Routine HIV testing increases ART uptake rates by $-\ln(1-test(t))$, where the proportion tested annually, $test\left( t \right)$, reaches 50% per year at 2031 and remains constant thereafter. Adherence support reduces virologic failure rates on first- and second-line ART. Virologic failure rates decrease until 2023, falling by 50% at 2020 and by 80% ultimately.

Individuals failing first-line ART switch to second-line regimens at rate $u^{Z}$. This rate increases over time to simulate expansion of second-line ART reaching universal access at 2021:

$u^{Z}(t)=\left\{ \begin{matrix} 0, & t\leq2004 \\ p^{Z}(t-2004)/12, & 2004<t\leq2016 \\ p^{Z}+(1-p^{Z})(t-2016)/5, & 2016<t<2021 \\ 1, & t>2021. \end{matrix} \right.$ (75)

The parameter $p^{Z}$ is set so that ~6% of ART patients are on second-line at 2016 [59]. The switching rate of 1 per year from 2021 on is consistent with South African guidelines for managing first-line ART virologic failure [39].

### Pre-exposure prophylaxis

We simulate PrEP implementation strategies that prioritize the DPV vaginal ring to specific female populations: i) women aged 22–45 years, ii) women aged 22–29 years, iii) women aged 22–45 years and having HIV incidence of at least 3% per year at 2017, or iv) female sex workers (FSWs) aged 22–45. The incidence-based strategy prioritizes PrEP to women aged 22–34 in the low and medium sexual activity levels, and to women aged 22–45 in the high sexual activity level. For the first three strategies, we specify a time-varying population-level coverage target $U^{P}\left( t \right)$ (the target proportion of the HIV-negative population aged 15–54 to cover):

$U^{P}\left( t \right)=p^{P}\times\left\{ \begin{matrix} 0, & t\leq t_{1}^{P} \\ \frac{t-t_{1}^{P}}{t_{2}^{P}-t_{1}^{P}}, & t_{1}^{P}<t\leq t_{2}^{P} \\ 1, & t_{2}^{P}<t\leq t_{3}^{P}. \end{matrix} \right.$ (76)

The PrEP coverage target increases linearly from time $t_{1}^{P}$ until $t_{2}^{P}$, then remains constant at level $p^{P}$ (range: 2.5%–10% of HIV-negative adult population overall) until PrEP rollout ends at time $t_{3}^{P}$. We then calculate the group-specific coverage targets $U_{i}^{P}\left( t \right)\propto U^{P}(t)$ needed to reach the population-level target; these group-specific target coverage levels are the same for each prioritized risk group $i$ of women, regardless of age or sexual activity level. We dynamically calculate uptake rates $u_{i}^{P}\left( t \right)$ so that PrEP coverage tracks the specified coverage targets:

$u_{i}^{P}\left( t \right)=max\left\{ 0, \left[ D_{i}^{P}(t)+\frac{d}{dt}D_{i}^{P}(t) \right]/X_{i}^{N}(t) \right\}$ . (77)

In Equation 77, $D_{i}^{P}(t)=\left[ U_{i}^{P}(t)(X_{i}^{N}\left( t \right)+\sum_{z=1}^{2} X_{i}^{P_{z}}(t))-\sum_{z=1}^{2} X_{i}^{P_{z}}(t) \right]$ is the difference between the target number and the actual number on PrEP at time $t$. To avoid circular dependencies when calculating the uptake rate $u_{i}^{P}\left( t \right)$, the temporal derivatives $\frac{d}{dt}D_{i}^{P}(t)$ are evaluated using Equations 4 and 5 with $u_{i}^{P}\left( t \right)=0$. An analogous approach is used to achieve PrEP coverage targets among FSWs.

# MODEL CALIBRATION

Our model is calibrated to longitudinal, age- and gender-stratified HIV prevalence [60] data and aggregate HIV incidence [61] data collected in the Africa Centre’s Demographic Surveillance site, as well as cross-sectional behavioral risk-stratified HIV prevalence data from South Africa’s Modes of Transmission Study [62]. We use Bayesian melding [63, 64] to estimate a posterior distribution over behavioral and epidemiological model parameters. Details of model calibration and validation have been described elsewhere [2]. We use the posterior mode input estimates in our analyses (Supplementary Table 1).

# SUPPLEMENTARY SENSITIVITY AND UNCERTAINTY ANALYSIS RESULTS

## HIV prevention and survival

A median 721,000 (interquartile range [IQR]: 623,000-811,000) infections occurred over the intervention horizon in the reference scenario. Implementation of 4%-71% effective PrEP [34, 35] when unprioritized among 22-45 year-olds (covering 5%-20% of adult women overall) prevented 4.1% (IQR: 2.8%-5.7%) of these infections. Impact increased to 5.5% (IQR: 3.8%-7.6%) when equivalent PrEP coverage levels were prioritized to 22-29 year-old women, and to 6.5% (IQR: 4.4%-9.0%) when PrEP was prioritized by incidence. Enrolling 10%-75% of 22-45 year-old FSWs in PrEP prevented 1.5% (IQR: 0.9%-2.3%) of infections, less than one quarter the impact of incidence-based PrEP (Supplementary Table 5). HIV prevention from incidence-based PrEP increased with greater PrEP coverage (standardized regression coefficient [SRC]: 0.75), average adherence (SRC: 0*.*42), and wild-type efficacy (SRC: 0.39); these inputs exerted similar influence on other PrEP strategies (Supplementary Table 7).

Over the lifetime horizon, the increases in discounted survival were maximal for incidence-based PrEP, having a median 327,000 life-years gained (IQR: 195,000-532,000; Supplementary Table 5). Lifetime survival estimates were most sensitive to discount rates; otherwise, drivers were similar to those for prevention (Supplementary Table 7).

## HIV drug resistance

By 2030, prevalent drug-resistant infections decreased compared to the reference scenario in 78%-85% of the simulations of unprioritized, age-based, or incidence-based PrEP strategies (Supplementary Table 5). Meanwhile, FSW-PrEP increased drug-resistance in 74% of simulations. Nevertheless, the number of drug-resistant cases did not change much compared to the reference scenario in sensitivity simulations, due to PrEP’s modest contribution to total resistance. Unprioritized PrEP decreased prevalent drug-resistant cases by a median of 1.2% (IQR: 0.4%-2%), while age-based and incidence-based PrEP decreased resistance by 1.4% (IQR: 0.3%-2.6%) and 1.5% (0.2%-2.9%), respectively. FSW-PrEP increased resistance by 0.4% (IQR 0%-1.2%).

Decreases in prevalent drug-resistant cases from incidence-based PrEP were larger with higher PrEP efficacy against wild-type HIV (SRC: 0.48), while decreases were smaller with more effective ART adherence support (SRC: -0.33) and more rapid second-line ART initiation after first-line ART failure (SRC: -0.23), as these factors reduce resistance from ART. These inputs had similar effect on resistance from other prioritized PrEP strategies (Supplementary Table 7). When prioritized by incidence, PrEP having wild-type efficacy ≥60% tended to decrease resistance (Supplementary Figure 3C). Higher PrEP efficacy was required for resistance to decrease at lower adherence levels. Results were similar for unprioritized and age-based PrEP (Supplementary Figure 3A-B), though the magnitude of changes in resistance was smaller compared to incidence-based PrEP, while PrEP prioritized to FSWs tended to increase resistance at any efficacy level (Supplementary Figure 3D).

## PrEP cost-effectiveness

Costs per life-year gained were lower in uncertainty analyses than in base-case analyses. As in base-case analyses, FSW-PrEP was cost-saving relative to the reference scenario without PrEP over both intervention and lifetime horizons in uncertainty analysis simulations (Supplementary Table 6). Meanwhile, incidence-based PrEP had lower cost and greater impact than either unprioritized PrEP or age-based PrEP over both horizons, and cost $33,920 (IQR: $23,799-$49,436) relative to FSW-PrEP over the intervention horizon. Over the lifetime horizon, incidence-based PrEP achieved greater impact and reduced costs compared to FSW-PrEP in 6% (605/10,000) of simulations. In the remaining 94% of simulations incidence-based PrEP cost $695 (IQR: $369-$1,245) per life-year gained relative to FSW-PrEP. The cost per life-year gained from PrEP decreased with rising PrEP adherence and efficacy but increased with greater ART effectiveness against HIV transmission and mortality (Supplementary Table 7).

The lower cost-effectiveness ratios seen in uncertainty analyses compared to base-case analyses are primarily due to inclusion in the base-case assumptions of 80% VMMC coverage and achievement of 90-90-90 targets for ART coverage and virologic suppression by 2020 and 95-95-95 by 2030. Thus, while our model estimated 412,399 new HIV infections during 2019-2030 without PrEP in the base-case reference scenario, it projected a median of 721,000 new infections over this same period in uncertainty analyses, which allowed for the possibility that South Africa may not reach these ambitious targets. Under unprioritized, incidence-based, or age-based PrEP strategies, costs per life-year gained were lowest when other prevention programs were less potent, resulting in more infections without PrEP implementation and less competition between PrEP and other prevention methods (Supplementary Figure 4A-C). Conversely, as other prevention interventions scaled-up in coverage and effectiveness, the number of new infections in the reference scenario without PrEP decreased, and PrEP's cost-effectiveness declined. Nevertheless, FSW-PrEP remained cost-saving regardless of scale-up of other prevention programs (Supplementary Figure 4D).

# SUPPLEMENTARY TABLES

Supplementary Table 1. Model behavioral, epidemiological, and demographic input parameters

| Parameter | Symbol | Value | Source |
| --- | --- | --- | --- |
| **Sexual behavior parameters** |  |  |  |
| Partner change rate (sex *g*, age *a*, activity level *k*) | $c_{g,a,k}$ | $\bar{c}\times c_{g,a}^{A}\times c_{g,k}^{K}$ |  |
| Average partner change rate, per year | $\bar{c}$ | 1.15 | [5] |
| Relative partner change rate by age (women, men) |  |  | [56, 65] |
| 15–19 | $c_{g,1}^{A}$ | 0.81, 0.10 |  |
| 20–24 | $c_{g,2}^{A}$ | 1.41, 0.76 |  |
| 25–29 | $c_{g,3}^{A}$ | 1.55, 1.87 |  |
| 30–34 | $c_{g,4}^{A}$ | 1.24, 2.18 |  |
| 35–39 | $c_{g,5}^{A}$ | 0.80, 1.49 |  |
| 40–44 | $c_{g,6}^{A}$ | 0.43, 0.69 |  |
| 45–49 | $c_{g,7}^{A}$ | 0.21, 0.23 |  |
| 50–54 | $c_{g,8}^{A}$ | 0.09, 0.06 |  |
| Relative partner change rate by sexual activity level (women, men) |  |  | [62, 66] |
| Least | $c_{g,1}^{K}$ | 0.33, 0.01 |  |
| Low | $c_{g,2}^{K}$ | 0.63, 0.32 |  |
| Medium | $c_{g,3}^{K}$ | 1.45, 3.88 |  |
| High | $c_{g,4}^{K}$ | 114.23, 13.54 |  |
| Proportion of individuals initially in each sexual activity level (women, men), % |  |  |  |
| Least | $\varphi_{g,1}$ | 10.4, 8.0 | [5] |
| Low | $\varphi_{g,2}$ | 38.6, 35.5 | Calculated |
| Medium | $\varphi_{g,3}$ | 0.8, 5.2 | [5, 7] |
| High | $\varphi_{g,4}$ | 0.2, 1.4 | [67, 68] |
| Average duration of female sex work, years | $1/\tau$ | 2.7 | [4] |
| Assortativity of sexual mixing by sexual activity level | $\alpha^{K}$ | 0.43 | [56] |
| Assortativity of sexual mixing between men and women of the same age | $\alpha^{A}$ | 0.30 | [56] |
| Assortativity of sexual mixing between younger women and men five years older | $\alpha^{D}$ | 0.76 | [56] |
| Number of sexual acts per regular partnership* | $n_{i,i^{'}}$ | 115 | [8, 69, 70] |
| Number of sexual acts per casual partnership* | $n_{i,i^{'}}$ | 12.4 | [62] |
| Number of sexual acts per short partnership* | $n_{i,i^{'}}$ | 3.3 | [62] |
| Condom use |  |  |  |
| Effectiveness of condoms for HIV prevention, % | $\varepsilon^{C}$ | 90 | [71] |
| Year that condom use starts to increase | $t_{1}^{C}$ | 1999.3 | [57] |
| Year that condom use stabilizes | $t_{2}^{C}$ | 2006.5 | [57] |
| Condom use frequency in regular partnerships* (initially, after increase), % | $\pi_{i,i^{'}}^{C}$ | 2, 46 | [5, 57] |
| Condom use frequency in casual partnerships* (initially, after increase), % | $\pi_{i,i^{'}}^{C}$ | 13, 52 | [5, 57] |
| Condom use frequency in short partnerships* (initially, after increase), % | $\pi_{i,i^{'}}^{C}$ | 34, 64 | [5, 57] |
|  |  |  |  |
| **Epidemiological parameters** |  |  |  |
| Epidemic start date | None | 1982 | Assumed |
| HIV transmission probability, % per sexual act |  |  |  |
| Acute infection ($h\in\{1,2\}$) | $\tilde{\beta}_{h}^{N,W}$ | 1.395 | [9, 72-75] |
| Chronic infection ($h\in\{3,4,5\}$) | $\tilde{\beta}_{h}^{N,W}$ | 0.247 | [9, 72-74, 76] |
| AIDS ($h=6$) | $\tilde{\beta}_{h}^{N,W}$ | 0.489 | [9, 72-74, 76] |
| HIV disease progression |  |  |  |
| Duration of acute infection pre-seroconversion, weeks | $1/\gamma_{1,W}$ | 3.01 | [77, 78] |
| Duration of acute infection post-seroconversion, weeks | $1/\gamma_{2,W}$ | 9.50 | [77, 78] |
| Time from onset of chronic infection until CD4 ≤ 500 cells/µL, years | $1/\gamma_{3,W}$ | 1.01 | [79] |
| Time from CD4 ≤ 500 cells/µL until CD4 ≤ 350 cells/µL, years | $1/\gamma_{4,W}$ | 3.00 | [79] |
| Time from CD4 ≤ 350 cells/µL until CD4 ≤ 200 cells/µL, years | $1/\gamma_{5,W}$ | 3.75 | [79] |
| Time from CD4 ≤ 200 cells/µL until death, years | $1/\gamma_{6,W}$ | 3.32 | [80] |
|  |  |  |  |
| **Demographic parameters** |  |  |  |
| Initial population size (simulation begins at 1978) | None | 2,466,000 |  |
| Initial population age distribution, % |  |  | [81] |
| 15–19 | None | 19 |  |
| 20–24 | None | 18 |  |
| 25–29 | None | 16 |  |
| 30–34 | None | 13 |  |
| 35–39 | None | 11 |  |
| 40–44 | None | 9 |  |
| 45–49 | None | 8 |  |
| 50–54 | None | 6 |  |
| HIV-unrelated mortality rates (women, men), per year |  |  | [82] |
| 15–19 | $\mu_{g,1}$ | 0.00076, 0.00179 |  |
| 20–24 | $\mu_{g,2}$ | 0.00129, 0.00370 |  |
| 25–29 | $\mu_{g,3}$ | 0.00182, 0.00480 |  |
| 30–34 | $\mu_{g,4}$ | 0.00250, 0.00574 |  |
| 35–39 | $\mu_{g,5}$ | 0.00313, 0.00687 |  |
| 40–44 | $\mu_{g,6}$ | 0.00444, 0.00930 |  |
| 45–49 | $\mu_{g,7}$ | 0.00572, 0.01161 |  |
| 50–54 | $\mu_{g,8}$ | 0.00958, 0.01690 |  |
| 55+ |  | 0.04694, 0.05828 |  |

Behavioral and epidemiological parameter values were calibrated via Bayesian melding as described in detail elsewhere [2]. Prior distributions on these inputs were informed by the sources listed.

* Partnerships are categorized as “regular” if at least one partner is in the least or low sexual activity level, “short” if both partners are in the high sexual activity level, and “casual” otherwise.

Supplementary Table 2. Model intervention-related input parameters

| Parameter | Symbol | Base-case value | LHS Range | | Source |
| --- | --- | --- | --- | --- | --- |
| **VMMC** |  |  |  | |  |
| Male circumcision prevalence at Jan. 1, 2021, % | None | 80 | 60–85 | | [7, 48, 49, 83] |
| VMMC effectiveness against male HIV acquisition, % | $\varepsilon^{M}$ | 60 | Not varied | | [50-52, 84] |
|  |  |  |  | |  |
| **ART** |  |  |  | |  |
| ART coverage by Jan 1, 2021, % | None | 81 | 58-84 | | [40, 41] |
| ART coverage by Jan 1, 2031, % | None | 90 | 72-96 | | [40] |
| ART effectiveness against HIV transmission while suppressed, % | $\varepsilon^{T}$ | 96 | 73–99 | | [43] |
| Rate individuals switch to 2^nd^-line ART after 1^st^-line ART virologic failure, per year | $u^{Z}$ | 1 | 0–2 | | [39] |
| Dropout rate during the first year of 1^st^-line ART, per year | $\eta^{T_{1}}$ | 0.15 | 0.1–0.2 | | [85] |
| Dropout rate during subsequent years of 1^st^-line ART, per year | $\eta^{T_{2}}$ | 0.08 | 0.04–0.12 | | [85] |
| Dropout rate while non-adherent to 1^st^-line ART, per year | $\eta^{T_{3}}$ | 0.08 | 0.04–0.12 | | [85] |
| Dropout rate on 2^nd^-line ART, per year | $\eta^{Z}$ | 0.091 | 0.06–0.13 | | [86, 87] |
| HIV mortality rate during the first year of 1^st^-line ART if ART initiated at CD4 ≤ 200 cells/µL, per year | $\mu_{6}^{T_{1}}$ | 0.15 | 0.1–0.2 | | [45] |
| HIV mortality rate during subsequent years of 1^st^-line ART if ART initiated at CD4 ≤ 200 cells/µL, per year | $\mu_{6}^{T_{2}}$ | 0.03 | 0.02–0.06 | | [45] |
| HIV mortality rate on 2^nd^-line ART if ART initiated at CD4 ≤ 200 cells/µL, per year | $\mu_{6}^{Z}$ | 0.036 | 0.02–0.07 | | [86, 87] |
| HIV mortality rate on ART if ART initiated at 201–350 CD4 cells/µL, relative to ART initiated at CD4 ≤ 200 cells/µL, % | $\mu_{5}^{T_{\bullet}}/\mu_{6}^{T_{\bullet}}$ | 33 | 15–85 | | [44] |
| HIV mortality rate on ART if ART initiated at 351–500 CD4 cells/µL, relative to ART initiated at 201–350 CD4 cells/µL, % | $\mu_{4}^{T_{\bullet}}/\mu_{5}^{T_{\bullet}}$ | 88 | 62.5–100 | | [46] |
| Virologic failure rate during the first year of 1^st^-line ART while harboring wild-type HIV, % per year | $1-exp(-\omega_{W}^{T_{1}})$ | 20 | 10–30 | | [47] |
| Virologic failure rate during subsequent years of 1^st^-line ART while harboring wild-type HIV, % per year | $1-exp(-\omega_{W}^{T_{2}})$ | 5 | 2.5–7.5 | | [88] |
| Virologic failure rate during the first year of 1^st^-line ART while harboring drug-resistant HIV, % per year | ${1-exp(-\omega}_{v}^{T_{1}})$ | 40 | 15–75 | | [31, 89] |
| Virologic failure rate during subsequent years of 1^st^-line ART while harboring drug-resistant HIV, % per year | ${1-exp(-\omega}_{v}^{T_{2}})$ | 10 | 3.75–22.5 | | [31] |
| Virologic failure rate on 2^nd^-line ART after 1^st^-line virologic failure due to non-adherence, % per year | $1-exp(-\omega_{v}^{Z})$ | 57 | 40–65 | | [86, 87] |
| Virologic failure rate on 2^nd^-line ART after 1^st^-line virologic failure due to drug resistance, % per year | $1-exp(-\omega_{v}^{Z})$ | 18 | 15–30 | | [86, 87] |
| Decrease in ART virologic failure due to adherence support, % | None | 80 | 0-90 | | [90] |
| Proportion of 1^st^-line ART virologic failure that is due to non-adherence, % | $1-\psi_{W}^{T_{\bullet}}$ | 30 | 20–40 | | [47, 91-93] |
| Proportion of 2^nd^-line ART virologic failure that is due to non-adherence after 1^st^-line virologic failure due to non-adherence, % | $1-\psi_{v}^{z}$ | 96 | 85–99 | | [54, 86, 87, 94] |
| Proportion of 2^nd^-line ART virologic failure that is due to non-adherence after 1^st^-line virologic failure due to drug resistance, % | $1-\psi_{v}^{z}$ | 92 | 80–95 | | [54, 86, 87, 94] |
| Fold increase in drug resistance risk on 2^nd^-line ART among persons with transmitted 2^nd^-line ART resistance (relative to wild-type HIV) | None | 7 | 2.5–18 | | [95] |
| DPV cross-resistance prevalence among persons with acquired resistance to 1^st^-line ART, % | $x_{W,W}$ | 80 | 70–100 | | [33] |
|  |  |  |  | |  |
| **PrEP** |  |  |  | |  |
| Time PrEP implementation begins, year | $t_{1}^{P}$ | 2019 | Not varied | | Assumed |
| Time PrEP implementation ends, year | $t_{3}^{P}$ | 2031 | Not varied | | Assumed |
| Time to reach target PrEP coverage, years | $t_{2}^{P}-t_{1}^{P}$ | 4 | 2–6 | | Assumed |
| PrEP uptake rate, per year | $u_{i}^{P}$ | Calculated | Calculated | |  |
| PrEP coverage (as level of HIV-negative adults aged 15–54), % | None | 2.5-10 | 2.5–10 | | Assumed |
| PrEP coverage of female sex workers (when prioritized), % | None | 25-75 | 10–75 | | Assumed |
| Duration of PrEP use, years | $1/\eta_{1}^{P}$ | 3 | 1–5 | | Assumed |
| PrEP dropout rate, per year | $\eta_{2}^{P}$ | 0.17 | 0.14–0.20 | | [96] |
| Rate that women leave the PrEP program, per year | $\eta^{P}$ | $\eta_{1}^{P}+\eta_{2}^{P}$ | $\eta_{1}^{P}+\eta_{2}^{P}$ | |  |
| Doses per year | None | 12 | Not varied | | [34, 35] |
| HIV testing frequency in the PrEP program, per year | $\sigma$ | 2 | 1–12 | | Assumed |
| PrEP efficacy against wild-type HIV, % | $\varepsilon_{W}^{P}$ | 75 | 20–90* | | [36] |
| PrEP efficacy against 1^st^-line ART-resistant HIV without PrEP cross-resistance, % | $\varepsilon_{R_{1}}^{P}$ | $\varepsilon_{W}^{P}$ | $\varepsilon_{W}^{P}$ | | [33] |
| PrEP efficacy against 1^st^-line ART-resistant HIV with PrEP cross-resistance, % | $\varepsilon_{C_{1}}^{P}$ | $\varepsilon_{W}^{P}$ | $0.5\varepsilon_{W}^{P}-\varepsilon_{W}^{P}$ | | [33] |
| PrEP efficacy against 2^nd^-line ART-resistant HIV, % | $\varepsilon_{S_{1}}^{P}$ | $\varepsilon_{W}^{P}$ | $\varepsilon_{W}^{P}$ | | Assumed |
| PrEP efficacy against PrEP-resistant HIV, % | $\varepsilon_{Q_{1}}^{P}$ | $\varepsilon_{W}^{P}$ | $0.5\varepsilon_{W}^{P}-\varepsilon_{W}^{P}$ | | [33] |
| Reduction in infectivity on PrEP during acute HIV infection, % | $\varepsilon_{v}^{S}$ | 0 | 0–0.5 | | [32, 38] |
| Average PrEP adherence (ASPIRE or RING scenario) | $\bar{\kappa}$ | 75 or 49 | 20–79* | | [34, 35] |
| Proportion of women who are non-adherent to PrEP, % | $\chi_{1}$ | 20 | 17–67 | | [34, 35] |
| Proportion of women who are adherent to PrEP, % | $\chi_{2}$ | 80 | 33–83 | | [34, 35] |
| PrEP adherence level if non-adherent, % | $\kappa_{1}$ | 0 | 0 | | [34, 35] |
| PrEP adherence level if adherent (ASPIRE or RING scenario), % | $\kappa_{2}$ | 94 or 61 | 60–95 | | [34, 35] |
| Time until PrEP resistance emerges in an entire HIV-positive cohort with perfect PrEP adherence, years | $\theta$ | 0.5 | 0.25–0.75 | | [38, 97, 98] |
| Rate that PrEP resistance emerges while harboring wild-type HIV or ART-resistant HIV without PrEP cross-resistance, per year | $\zeta_{W}^{P_{z}}$, $\zeta_{R_{1}}^{P_{z}}$, $\zeta_{S_{1}}^{P_{z}}$ | $-\ln\left( 1-0.99\kappa_{z} \right)/\theta$ | | | Calculated |
| Rate that PrEP resistance emerges while harboring reverted to wild-type PrEP-resistant or cross-resistant HIV, per year | $\zeta_{c_{1}}^{P_{z}}$, $\zeta_{q_{1}}^{P_{z}}$ | $-2\ln\left( 1-0.99\kappa_{z} \right)/\theta$ | | | Assumed |
|  |  |  | |  |  |
| **HIV Drug Resistance** |  |  | |  |  |
| Persistence time of transmitted resistance to PrEP, years | $1/\xi_{Q_{1}}$ | 18 | | 5–65 | [16] |
| Persistence time of acquired resistance to PrEP, years | $1/\xi_{Q_{2}}$ | 1 | | 0.5–5.0 | [14, 18] |
| Persistence time of transmitted resistance to 1^st^-line ART, years | $1/{\xi_{R}}_{1}$, $1/\xi_{C_{1}}$ | 18 | | 5–65 | [16] |
| Persistence time of acquired resistance to 1^st^-line ART, years | $1/{\xi_{R}}_{2}$, $1/\xi_{C_{2}}$ | 1 | | 0.5–5.0 | [14, 18] |
| Persistence time of transmitted resistance to 2^nd^-line ART, years | $1/\xi_{S_{1}}$ | 5 | | 2–14 | [16, 54, 94, 99] |
| Persistence time of acquired resistance to 2^nd^-line ART, years | $1/\xi_{S_{2}}$ | 0.6 | | 0.3–1.7 | [13, 14, 54, 94, 99] |
| Infectivity of HIV with transmitted resistance to 1^st^- or 2^nd^-line ART, relative to wild-type HIV ($v\in\{R_{1},C_{1},S_{1}\}$), % | $\tilde{\beta}_{h}^{N,v}/\tilde{\beta}_{h}^{N,W}$ | 100 | | 50–100 | Assumed |
| Infectivity of HIV with acquired resistance to 1^st^- or 2^nd^-line ART, relative to wild-type HIV ($v\in\{R_{2},C_{2},S_{2}\})$, % | $\tilde{\beta}_{h}^{N,v}/\tilde{\beta}_{h}^{N,W}$ | 75 | | 50–100 | [30, 99, 100] |
| Infectivity of HIV with transmitted resistance to PrEP, relative to wild-type HIV, % | $\tilde{\beta}_{h}^{N,Q_{1}}/\tilde{\beta}_{h}^{N,W}$ | 100 | | 75–100 | Assumed |
| Infectivity of HIV with acquired resistance to PrEP, relative to wild-type HIV, % | $\tilde{\beta}_{h}^{N,Q_{2}}/\tilde{\beta}_{h}^{N,W}$ | 90 | | 75–100 | [101-103] |
| Disease progression rate with HIV having transmitted resistance to 1^st^- or 2^nd^-line ART, relative to wild-type HIV ($v\in\{R_{1},C_{1},S_{1}\}$), % | ${\gamma_{h,v}}/{\gamma_{h,W}}$ | 100 | | 50–100 | Assumed |
| Disease progression rate with HIV having acquired resistance to 1^st^- or 2^nd^-line ART, relative to wild-type HIV ($v\in\{R_{2},C_{2},S_{2}\}$), % | ${\gamma_{h,v}}/{\gamma_{h,W}}$ | 75 | | 50–100 | [30, 99, 100] |
| Disease progression rate with HIV having transmitted resistance to PrEP, relative to wild-type HIV, % | ${\gamma_{h,Q_{1}}}/{\gamma_{h,W}}$ | 100 | | 75–100 | Assumed |
|  |  |  | |  |  |
| **Costs (2017 US$)** |  |  | |  |  |
| Annual discount rate, % | None | 3 | | 1–5 | [104] |
| Outpatient ART costs, $ per person-year |  |  | |  |  |
| 1^st^-line ART costs (includes ARVs), $ per person-year | None | 279 | | 140–419 | [105-107] |
| 2^nd^-line ART costs (includes ARVs), $ per person-year | None | 558 | | 279–837 | [105-107] |
| 1^st^-line ARV costs (TDF+3TC+EFV), $ per person-year | None | 99 | | 82–115 | [107] |
| 2^nd^-line ARV costs (ZDV+3TC+LPV/r), $ per person-year | None | 267 | | 259–275 | [107] |
| HIV testing (HIV+ result) and linkage to care, $ per ART initiator | None | 27 | | Not varied | [108] |
| HIV testing (HIV– result), $ per test | None | 12 | | Not varied | [108] |
| Adherence support costs, $ per person-year | None | 50 | | 0–200 | [105, 108, 109] |
| Inpatient HIV healthcare costs, $ per person-year |  |  | |  |  |
| Not on ART, CD4 > 350 cells/µL | None | 62 | | 36–120 | [110] |
| Not on ART, CD4 201–350 cells/µL | None | 92 | | 62–154 | [110] |
| Not on ART, CD4 ≤ 200 cells/µL | None | 192 | | 98–314 | [110] |
| On ART, CD4 > 350 cells/µL | None | 71 | | 36–127 | [110] |
| On ART, CD4 201–350 cells/µL | None | 176 | | 106–268 | [110] |
| On ART, CD4 ≤ 200 cells/µL | None | 413 | | 168–815 | [110] |
| VMMC costs, $ per circumcision | None | 149 | | 135–162 | [111] |
| PrEP costs, $ per person-year | None | 131 | | 119–143 | [112, 113] |
| HIV-unrelated healthcare costs, $ per person-year | None | 225 | | 113–338 | [114] |

3TC, lamivudine; ART, antiretroviral therapy; ARV, antiretroviral drug; EFV, efavirenz; LHS, Latin hypercube sampling; LPV/r, lopinavir/ritonavir; PrEP, pre-exposure prophylaxis; VMMC, voluntary medical male circumcision; ZDV, zidovudine.

* PrEP efficacy and average adherence were drawn from truncated normal distributions (with medians of 75% efficacy and 62% adherence and the stated ranges) in sensitivity analysis. All other inputs were uniformly distributed.

Supplementary Table 3. Model drug resistance dynamics

| Preexisting variant | DR emerges on PrEP | DR emerges on 1^st^-line ART | DR emerges on 2^nd^-line ART | DR reverts off ARVs | DR reverts on PrEP | DR reverts on 1^st^-line ART | DR reverts on 2^nd^-line ART | HIV Transmission |
| --- | --- | --- | --- | --- | --- | --- | --- | --- |
| $C_{1}/C_{1}$ | … | $C_{2}/C_{2}$ | $S_{2}/S_{2}$ | $c_{1}/c_{1}$ | $c_{1}/C_{1}$ | … | $c_{1}/c_{1}$ | $C_{1}/C_{1}$ |
| $c_{1}/C_{1}$ | … | $C_{2}/C_{2}$ | $S_{2}/S_{2}$ | $c_{1}/c_{1}$ | … | … | $c_{1}/c_{1}$ | $C_{1}/C_{1}$ |
| $c_{1}/c_{1}$ | $c_{1}/Q_{2}$ | $C_{2}/C_{2}$ | $S_{2}/S_{2}$ | … | … | … | … | $W/W$ |
| $c_{1}/Q_{2}$ | … | $C_{2}/C_{2}$ | $S_{2}/S_{2}$ | $c_{1}/q_{2}$ | … | … | $c_{1}/q_{2}$ | $Q_{1}/Q_{1}$ |
| $c_{1}/q_{2}$ | $c_{1}/Q_{2}$ | $C_{2}/C_{2}$ | $S_{2}/S_{2}$ | … | … | … | … | $W/W$ |
| $C_{2}/C_{2}$ | … | … | $S_{2}/S_{2}$ | $c_{2}/c_{2}$ | … | … | $c_{2}/c_{2}$ | $C_{1}/C_{1}$ |
| $c_{2}/c_{2}$ | … | $C_{2}/C_{2}$ | $S_{2}/S_{2}$ | … | … | … | … | $W/W$ |
| $Q_{1}/Q_{1}$ | … | $C_{2}/C_{2}$ | $S_{2}/S_{2}$ | $q_{1}/q_{1}$ | $q_{1}/Q_{1}$ | … | $q_{1}/q_{1}$ | $Q_{1}/Q_{1}$ |
| $q_{1}/Q_{1}$ | … | $C_{2}/C_{2}$ | $S_{2}/S_{2}$ | $q_{1}/q_{1}$ | … | … | $q_{1}/q_{1}$ | $Q_{1}/Q_{1}$ |
| $q_{1}/q_{1}$ | $q_{1}/Q_{2}$ | $C_{2}/C_{2}$ | $S_{2}/S_{2}$ | … | … | … | … | $W/W$ |
| $q_{1}/Q_{2}$ | … | $C_{2}/C_{2}$ | $S_{2}/S_{2}$ | $q_{1}/q_{2}$ | … | … | $q_{1}/q_{2}$ | $Q_{1}/Q_{1}$ |
| $q_{1}/q_{2}$ | $q_{1}/Q_{2}$ | $C_{2}/C_{2}$ | $S_{2}/S_{2}$ | … | … | … | … | $W/W$ |
| $R_{1}/R_{1}$ | $R_{1}/Q_{2}$ | $R_{2}/R_{2}$ or $C_{2}/C_{2}$ | $S_{2}/S_{2}$ | $r_{1}/r_{1}$ | $r_{1}/r_{1}$ | … | $r_{1}/r_{1}$ | $R_{1}/R_{1}$ |
| $R_{1}/Q_{2}$ | … | $C_{2}/C_{2}$ | $S_{2}/S_{2}$ | $r_{1}/Q_{2}$ or $R_{1}/q_{2}$ | $r_{1}/Q_{2}$ | … | $r_{1}/Q_{2}$ or $R_{1}/q_{2}$ | $Q_{1}/Q_{1}$ |
| $R_{1}/q_{2}$ | $R_{1}/Q_{2}$ | $C_{2}/C_{2}$ | $S_{2}/S_{2}$ | $r_{1}/q_{2}$ | … | … | $r_{1}/q_{2}$ | $R_{1}/R_{1}$ |
| $r_{1}/r_{1}$ | $r_{1}/Q_{2}$ | $R_{2}/R_{2}$ or $C_{2}/C_{2}$ | $S_{2}/S_{2}$ | … | … | … | … | $W/W$ |
| $r_{1}/Q_{2}$ | … | $C_{2}/C_{2}$ | $S_{2}/S_{2}$ | $r_{1}/q_{2}$ | … | … | $r_{1}/q_{2}$ | $Q_{1}/Q_{1}$ |
| $r_{1}/q_{2}$ | $r_{1}/Q_{2}$ | $C_{2}/C_{2}$ | $S_{2}/S_{2}$ | … | … | … | … | $W/W$ |
| $R_{2}/R_{2}$ | … | … | $S_{2}/S_{2}$ | $r_{2}/r_{2}$ | … | … | $r_{2}/r_{2}$ | $R_{1}/R_{1}$ |
| $r_{2}/r_{2}$ | … | $R_{2}/R_{2}$ | $S_{2}/S_{2}$ | … | … | … | … | $W/W$ |
| $S_{1}/S_{1}$ | $S_{1}/Q_{2}$ | $R_{2}/R_{2}$ or $C_{2}/C_{2}$ | $S_{2}/S_{2}$ | $s_{1}/s_{1}$ | $s_{1}/s_{1}$ | … | … | $S_{1}/S_{1}$ |
| $S_{1}/Q_{2}$ | … | $C_{2}/C_{2}$ | $S_{2}/S_{2}$ | $s_{1}/Q_{2}$ or $S_{1}/q_{2}$ | $s_{1}/Q_{2}$ | … | $S_{1}/q_{2}$ | $Q_{1}/Q_{1}$ |
| $S_{1}/q_{2}$ | $S_{1}/Q_{2}$ | $C_{2}/C_{2}$ | $S_{2}/S_{2}$ | $s_{1}/q_{2}$ | … | … | … | $S_{1}/S_{1}$ |
| $s_{1}/s_{1}$ | $s_{1}/Q_{2}$ | $R_{2}/R_{2}$ or $C_{2}/C_{2}$ | $S_{2}/S_{2}$ | … | … | … | … | $W/W$ |
| $s_{1}/Q_{2}$ | … | $C_{2}/C_{2}$ | $S_{2}/S_{2}$ | $s_{1}/q_{2}$ | … | … | $s_{1}/q_{2}$ | $Q_{1}/Q_{1}$ |
| $s_{1}/q_{2}$ | $s_{1}/Q_{2}$ | $C_{2}/C_{2}$ | $S_{2}/S_{2}$ | … | … | … | … | $W/W$ |
| $S_{2}/S_{2}$ | … | … | … | $s_{2}/s_{2}$ | … | … | … | $S_{1}/S_{1}$ |
| $s_{2}/s_{2}$ | … | … | $S_{2}/S_{2}$ | … | … | … | … | $W/W$ |
| $W/W$ | $W/Q_{2}$ | $R_{2}/R_{2}$ or $C_{2}/C_{2}$ | $S_{2}/S_{2}$ | … | … | … | … | $W/W$ |
| $W/Q_{2}$ | … | $C_{2}/C_{2}$ | $S_{2}/S_{2}$ | $W/q_{2}$ | … | … | $W/q_{2}$ | $Q_{1}/Q_{1}$ |
| $W/q_{2}$ | $W/Q_{2}$ | $C_{2}/C_{2}$ | $S_{2}/S_{2}$ | … | … | … | … | $W/W$ |

ART, antiretroviral therapy; DR, drug resistance; PrEP, pre-exposure prophylaxis.

Each table row lists the blood/genital tract HIV variant combinations that may result from model dynamic processes acting on a preexisting variant combination. Processes that do not apply to a given combination, such as PrEP resistance emergence in a host who has already acquired PrEP resistance, are indicated with ellipses (“…”).

See Section 3 for explanation of abbreviations.

Supplementary Table 4. Results of base-case analysis

|  | Intervention horizon | | | Lifetime horizon | | Prevalent drug-resistance at 2031 | |
| --- | --- | --- | --- | --- | --- | --- | --- |
| **Intervention** | **New Infections, n** | **Life-years lived, thousands** | **Total costs, millions $** | **Life-years lived, thousands** | **Total costs, millions $** | **Total DR cases, n** | **PrEP DR cases, n** |
| No PrEP (reference) | 412,399 | 70,444 | 23,286 | 197,641 | 60,543 | 88,694 | 0 |
|  |  |  |  |  |  |  |  |
| **Intervention (PrEP coverage^a^)** | **Infections prevented, %** | **Life-years gained, thousands** | **Cost increases, millions $** | **Life-years gained, thousands** | **Cost increases, millions $** | **Total DR increase, %** | **PrEP DR cases, n** |
| ASPIRE PrEP scenario ^b^ |  |  |  |  |  |  |  |
| Unprioritized PrEP (2.5%) | 2.3 | 2.1 | 145.8 | 45 | 106.6 | -0.5 | 690 |
| Unprioritized PrEP (5%) | 4.7 | 4.2 | 291.9 | 90 | 213.2 | -1.0 | 1,372 |
| Unprioritized PrEP (7.5%) | 7.1 | 6.3 | 438.2 | 135 | 319.6 | -1.5 | 2,045 |
| Unprioritized PrEP (10%) | 9.5 | 8.4 | 584.8 | 181 | 426.0 | -2.0 | 2,708 |
|  |  |  |  |  |  |  |  |
| Age-based PrEP (2.5%) | 3.2 | 2.8 | 140.5 | 65 | 83.9 | -0.6 | 1,068 |
| Age-based PrEP (5%) | 6.4 | 5.6 | 281.3 | 130 | 168.0 | -1.1 | 2,102 |
| Age-based PrEP (7.5%) | 9.6 | 8.5 | 422.5 | 195 | 252.5 | -1.8 | 3,097 |
| Age-based PrEP (10%) | 12.7 | 11.3 | 564.2 | 260 | 337.4 | -2.4 | 4,049 |
|  |  |  |  |  |  |  |  |
| Incidence-based PrEP (2.5%) | 3.8 | 3.3 | 139.0 | 74 | 73.1 | -0.5 | 1,363 |
| Incidence-based PrEP (5%) | 7.6 | 6.6 | 278.4 | 149 | 146.6 | -1.1 | 2,664 |
| Incidence-based PrEP (7.5%) | 11.3 | 10.0 | 418.4 | 223 | 220.4 | -1.7 | 3,895 |
| Incidence-based PrEP (10%) | 15.1 | 13.3 | 558.9 | 297 | 295.0 | -2.4 | 5,047 |
|  |  |  |  |  |  |  |  |
| PrEP to FSWs (25%) | 1.7 | 1.2 | -5.8 | 32 | -37.0 | 0.8 | 1,468 |
| PrEP to FSWs (50%) | 3.5 | 2.5 | -12.1 | 66 | -77.5 | 1.5 | 2,989 |
| PrEP to FSWs (75%) | 5.6 | 3.9 | -18.9 | 104 | -121.9 | 2.2 | 4,531 |
|  |  |  |  |  |  |  |  |
| RING PrEP scenario ^b^ |  |  |  |  |  |  |  |
| Unprioritized PrEP (2.5%) | 1.4 | 1.3 | 150.2 | 27 | 127.0 | 0.0 | 656 |
| Unprioritized PrEP (5%) | 2.9 | 2.6 | 300.6 | 54 | 253.9 | -0.1 | 1,311 |
| Unprioritized PrEP (7.5%) | 4.3 | 4.0 | 451.2 | 82 | 380.8 | -0.1 | 1,962 |
| Unprioritized PrEP (10%) | 5.8 | 5.3 | 601.9 | 110 | 507.7 | -0.2 | 2,610 |
|  |  |  |  |  |  |  |  |
| Age-based PrEP (2.5%) | 1.9 | 1.8 | 146.5 | 39 | 112.7 | 0.1 | 1,012 |
| Age-based PrEP (5%) | 3.9 | 3.5 | 293.2 | 78 | 225.8 | 0.1 | 2,003 |
| Age-based PrEP (7.5%) | 5.8 | 5.3 | 440.2 | 118 | 339.1 | 0.1 | 2,971 |
| Age-based PrEP (10%) | 7.7 | 7.0 | 587.4 | 157 | 452.7 | 0.1 | 3,913 |
|  |  |  |  |  |  |  |  |
| Incidence-based PrEP (2.5%) | 2.3 | 2.1 | 146.1 | 45 | 107.0 | 0.2 | 1,282 |
| Incidence-based PrEP (5%) | 4.6 | 4.1 | 292.4 | 89 | 214.2 | 0.4 | 2,527 |
| Incidence-based PrEP (7.5%) | 6.9 | 6.2 | 439.1 | 134 | 321.7 | 0.5 | 3,729 |
| Incidence-based PrEP (10%) | 9.1 | 8.3 | 586.2 | 179 | 429.6 | 0.6 | 4,882 |
|  |  |  |  |  |  |  |  |
| PrEP to FSWs (25%) | 1.0 | 0.7 | -2.8 | 18 | -20.4 | 1.0 | 1,312 |
| PrEP to FSWs (50%) | 2.0 | 1.5 | -5.8 | 37 | -42.2 | 2.0 | 2,669 |
| PrEP to FSWs (75%) | 3.1 | 2.2 | -9.1 | 58 | -65.5 | 2.9 | 4,057 |

DR, drug resistance; FSW, female sex worker; PrEP, pre-exposure prophylaxis

Absolute outcomes are shown for the reference scenario. Increases relative to the reference scenario are shown for PrEP interventions. Costs and life-years lived are discounted 3% annually. Costs are in 2017 US$.

^a^PrEP coverage levels are stated as a proportion of adults (2.5%, 5%, 7.5%, or 10%) for unprioritized, age-based, or incidence-based PrEP (corresponding to 10%-40% coverage of women aged 22-45 when unprioritized or 20%-75% of prioritized populations), or as a proportion of FSWs (25%-75% of FSWs).

^b^ASPIRE and The Ring Study (RING) PrEP scenarios simulate 56% or 37% effective PrEP, respectively.

Supplementary Table 5. Uncertainty analysis outcomes

|  | Unprioritized PrEP | Age-based PrEP | Incidence-based PrEP | PrEP to FSWs |
| --- | --- | --- | --- | --- |
| ***Intervention horizon*** |  |  |  |  |
| Undiscounted infections prevented, % | 4.1 (2.8-5.7) | 5.5 (3.8-7.6) | 6.5 (4.4-9.0) | 1.5 (0.9-2.3) |
| Discounted life-years gained | 7,000 (4,444-10,708) | 9,342 (5,942-14,276) | 10,989 (6,978-16,788) | 2,020 (1,149-3,329) |
| Discounted incremental cost, millions $ | 332.2 (232.4 to 433.5) | 316.3 (221.6 to 413.2) | 309.7 (216.8 to 405.8) | -8.8 (-14.4 to -5.0) |
| % of simulations in which PrEP cost <$500 per life-year gained | 0  (0/10,000) | 0  (0/10,000) | 0  (0/10,000) | 100  (9,997/10,000) |
| % of simulations in which PrEP was cost-saving | 0 (0/10,000) | 0 (0/10,000) | 0 (0/10,000) | 100 (9,996/10,000) |
| Cost per life-year gained, $* | 44,865 (32,026-64,500) | 32,112 (22,694-46,238) | 26,808 (18,867-38,829) | cost-saving |
| % of simulations in which DR decreased by 2030 | 85 (8,482/10,000) | 80 (8,023/10,000) | 78 (7,800/10,000) | 26 (2,646/10,000) |
| Decrease in prevalent DR cases by 2030, % | 1.2 (0.4 to 2.0) | 1.4 (0.3 to 2.6) | 1.5 (0.2 to 2.9) | -0.4 (-1.2 to 0.0) |
|  |  |  |  |  |
| ***Lifetime horizon*** |  |  |  |  |
| Discounted life-years gained | 199,110 (119,254-321,946) | 286,763 (171,284-466,232) | 327,491 (195,427-531,699) | 68,804 (37,410-119,126) |
| Discounted incremental cost, millions $ | 203.3 (133.8 to 296.1) | 144.7 (74.5 to 235.1) | 115.7 (38.6 to 205.4) | -48.3 (-81.9 to -26.4) |
| % of simulations in which PrEP cost <$500 per life-year gained | 19  (1,943/10,000) | 49  (4,910/10,000) | 61  (6,099/10,000) | 100  (9,997/10,000) |
| % of simulations in which PrEP was cost-saving | 1 (119/10,000) | 9 (905/10,000) | 16 (1,637/10,000) | 100 (9,982/10,000) |
| Cost per life-year gained, $* | 1,045 (603-1,741) | 580 (295-1,040) | 464 (227-869) | cost-saving |

DR, drug resistance; FSW, female sex workers; PrEP, pre-exposure PrEP

Medians (interquartile ranges) of model outcomes or proportions (number/10,000) of simulations are shown

* ICER statistics exclude simulations in which PrEP was cost-saving.

Supplementary Table 6. PrEP cost-effectiveness in uncertainty analysis simulations

|  | Cost per life-year gained relative to reference scenario, $ | Cost per life-year gained relative to the next best strategy, $ |
| --- | --- | --- |
| ***Intervention horizon*** |  |  |
| No PrEP | (reference) | (reference) |
| PrEP to FSWs | cost-saving | cost-saving |
| Unprioritized PrEP | 44,865 (32,026-64,500) | dominated |
| Age-based PrEP | 32,112 (22,694-46,238) | dominated |
| Incidence-based PrEP | 26,808 (18,867-38,829) | 33,920 (23,799-49,436) |
|  |  |  |
| ***Lifetime horizon*** |  |  |
| No PrEP | (reference) | (reference) |
| PrEP to FSWs | cost-saving | cost-saving |
| Unprioritized PrEP | 1,045 (603-1,741) | dominated |
| Age-based PrEP | 580 (295-1,040) | dominated |
| Incidence-based PrEP | 464 (227-869) | 695 (369-1,245) |

* ICER statistics exclude simulations in which PrEP was cost-saving (Supplementary Table 5). Incidence-based PrEP was cost-saving relative to FSW PrEP in 6% (605/10,000) of simulations over the lifetime horizon and 0% (0/10,000) simulations over the intervention horizon.

Supplementary Table 7. Results of sensitivity analysis: drivers of key model outcomes

|  | Standardized Regression Coefficients (SRCs) | | | |
| --- | --- | --- | --- | --- |
| **Model input** | **Unprioritized PrEP** | **Age-based PrEP** | **Incidence-based PrEP** | **PrEP to FSWs** |
| ***Infections prevented, intervention horizon*** |  |  |  |  |
| PrEP coverage | 0.76 | 0.75 | 0.75 | 0.78 |
| Average PrEP adherence | 0.42 | 0.42 | 0.42 | 0.34 |
| PrEP efficacy against wild-type HIV | 0.39 | 0.39 | 0.39 | 0.30 |
|  |  |  |  |  |
| ***Life-years gained, intervention horizon*** |  |  |  |  |
| PrEP coverage | 0.55 | 0.55 | 0.55 | 0.65 |
| Mortality on ART initiated at CD4>200 cells/µL | 0.45 | 0.45 | 0.45 | 0.40 |
| Average PrEP adherence | 0.30 | 0.30 | 0.30 | 0.27 |
| PrEP efficacy against wild-type HIV | 0.27 | 0.27 | 0.27 | 0.23 |
| Time to reach target PrEP coverage | -0.27 | -0.27 | -0.27 | -0.22 |
|  |  |  |  |  |
| ***Incremental cost, intervention horizon*** |  |  |  |  |
| PrEP coverage | 0.94 | 0.93 | 0.92 | -0.69 |
| Average PrEP adherence | … | … | … | -0.34 |
| PrEP efficacy against wild-type HIV | … | … | … | -0.31 |
| 1^st^-line ART costs | … | … | … | -0.24 |
|  |  |  |  |  |
| ***Cost per life-year gained, intervention horizon**** |  |  |  |  |
| Mortality on ART initiated at CD4>200 cells/µL | -0.51 | -0.50 | -0.49 |  |
| Average PrEP adherence | -0.42 | -0.43 | -0.43 |  |
| PrEP efficacy against wild-type HIV | -0.40 | -0.40 | -0.40 |  |
| ART effectiveness against HIV transmission | 0.28 | 0.28 | 0.28 |  |
|  |  |  |  |  |
| ***Decrease in drug resistance cases by 2030*** |  |  |  |  |
| PrEP efficacy against wild-type HIV | 0.50 | 0.48 | 0.48 | 0.33 |
| Reduction in virologic failure due to ART adherence support | -0.33 | -0.33 | -0.33 | -0.31 |
| PrEP coverage | 0.27 | … | … | -0.23 |
| Rate of 2^nd^-line ART initiation after 1^st^-line ART failure | … | -0.23 | -0.23 | -0.24 |
|  |  |  |  |  |
| ***Life-years gained, lifetime horizon*** |  |  |  |  |
| Discount rate | -0.55 | -0.55 | -0.55 | -0.49 |
| PrEP coverage | 0.49 | 0.48 | 0.49 | 0.59 |
| Average PrEP adherence | 0.27 | 0.27 | 0.27 | 0.26 |
| PrEP efficacy against wild-type HIV | 0.26 | 0.26 | 0.26 | 0.24 |
|  |  |  |  |  |
| ***Incremental cost, lifetime horizon*** |  |  |  |  |
| PrEP coverage | 0.66 | 0.41 | 0.28 | -0.63 |
| ART effectiveness against HIV transmission | 0.27 | 0.30 | 0.31 | … |
| Average PrEP adherence | -0.27 | -0.33 | -0.35 | -0.27 |
| PrEP efficacy against wild-type HIV | -0.25 | -0.31 | -0.33 | -0.26 |
| Mortality on ART initiated at CD4>200 cells/µL | 0.23 | 0.29 | 0.30 | … |
| 1^st^-line ART costs | … | -0.24 | -0.25 | … |
| ART adherence support costs | … | -0.23 | -0.24 | … |
| Discount rate | … | … | … | 0.29 |
|  |  |  |  |  |
| ***Cost per life-year gained, lifetime horizon**** |  |  |  |  |
| PrEP efficacy against wild-type HIV | -0.41 | -0.36 | -0.30 |  |
| Average PrEP adherence | -0.39 | -0.33 | -0.26 |  |
| Discount rate | 0.38 | 0.29 | … |  |
| ART effectiveness against HIV transmission | 0.31 | 0.25 | … |  |

ART, antiretroviral therapy; PrEP, pre-exposure prophylaxis

Model inputs that explained at least 5% (SRC^2^≥0.05) of outcome variance are shown.

* Cost-saving simulations were excluded from regression analysis of ICERs, including all simulations of PrEP prioritized to FSWs, as magnitudes of these ICERs are meaningless [115]. Regression analysis of ICERs of FSW-PrEP are not reported because PrEP was cost-saving in almost all simulations (Supplementary Table 5).

# SUPPLEMENTARY FIGURES


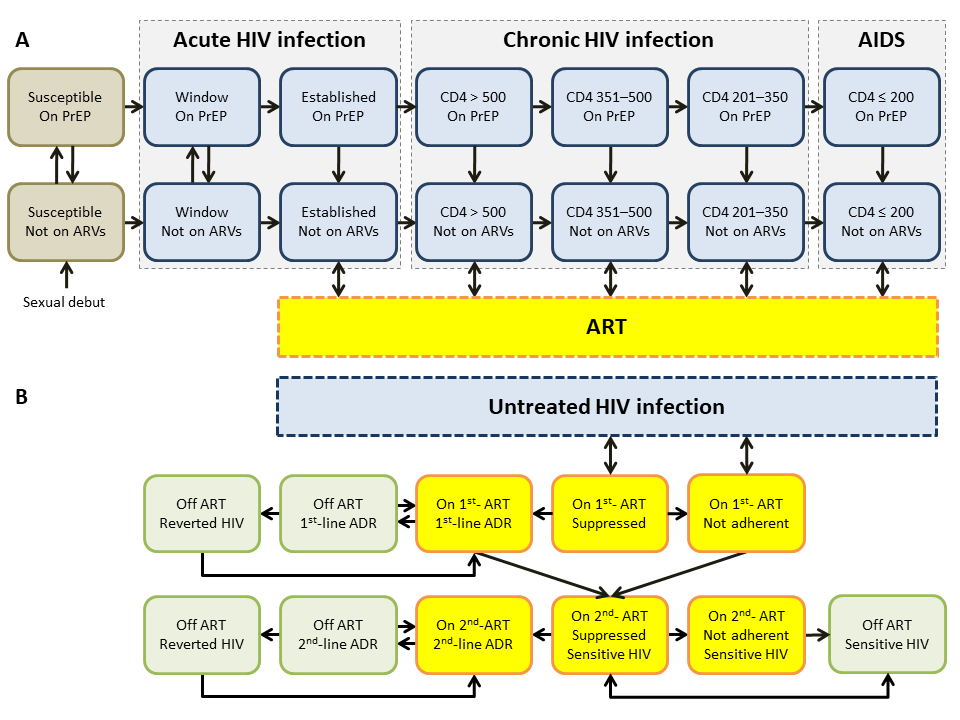


Supplementary Figure 1. Simplified model flow diagram of HIV disease progression and ART use. Individuals debut in the model HIV-negative and ARV-naïve (A). HIV-negative individuals and individuals with undetected window-period acute infection may initiate PrEP. HIV-positive individuals proceed through six consecutive stages of HIV infection. Individuals with detectable HIV may initiate ART (B). Individuals who fail first-line ART (due to non-adherence or drug resistance emergence) may switch to second-line ART. PrEP-related resistance dynamics are omitted for clarity. Compartments of ART-naïve people living with HIV are shown in blue, compartments on ART are shown in yellow, and compartments of individuals who have stopped ART after virologic failure are shown in green. ADR, acquired drug resistance. ART, antiretroviral therapy; ARVs, antiretroviral drugs; PrEP, pre-exposure prophylaxis.

**
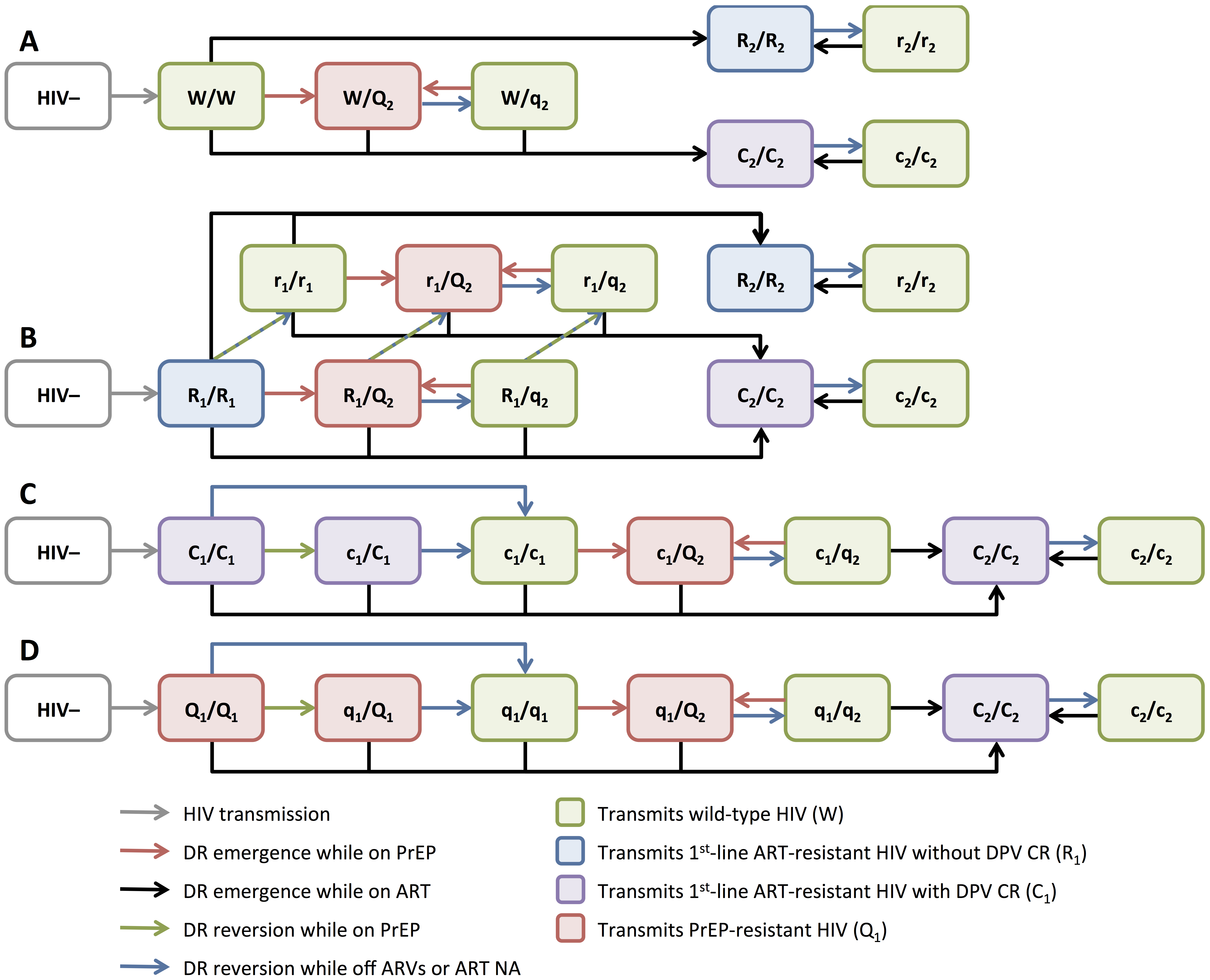
**

Supplementary Figure 2. Flow diagram of model drug resistance dynamics. Boxes represent HIV-negative individuals [HIV–] or HIV-positive individuals, stratified by HIV variants in blood/genital bodily compartments. Box colors indicate the variant that individuals transmit. The HIV variants shown consist of wild-type [W], resistance to first-line ART without [R_•_] or with [C_•_] DPV cross-resistance, or resistance to PrEP [Q_•_]. Subscripts distinguish transmitted [e.g., R_1_] from acquired [R_2_] resistance. Lower-case letters denote minority variants. HIV-negative individuals may become infected with wild-type HIV (A), HIV with TDR to first-line ART without or with DPV cross-resistance (B and C, respectively) or TDR to DPV PrEP (D). HIV resistance dynamics for individuals harboring TDR to second-line ART are analogous to first-line ART without cross-resistance (B). ART, antiretroviral therapy; CR, cross-resistance; DPV, dapivirine; DR, drug resistance; NA, non-adherent; PrEP, pre-exposure prophylaxis; TDR, transmitted drug resistance.


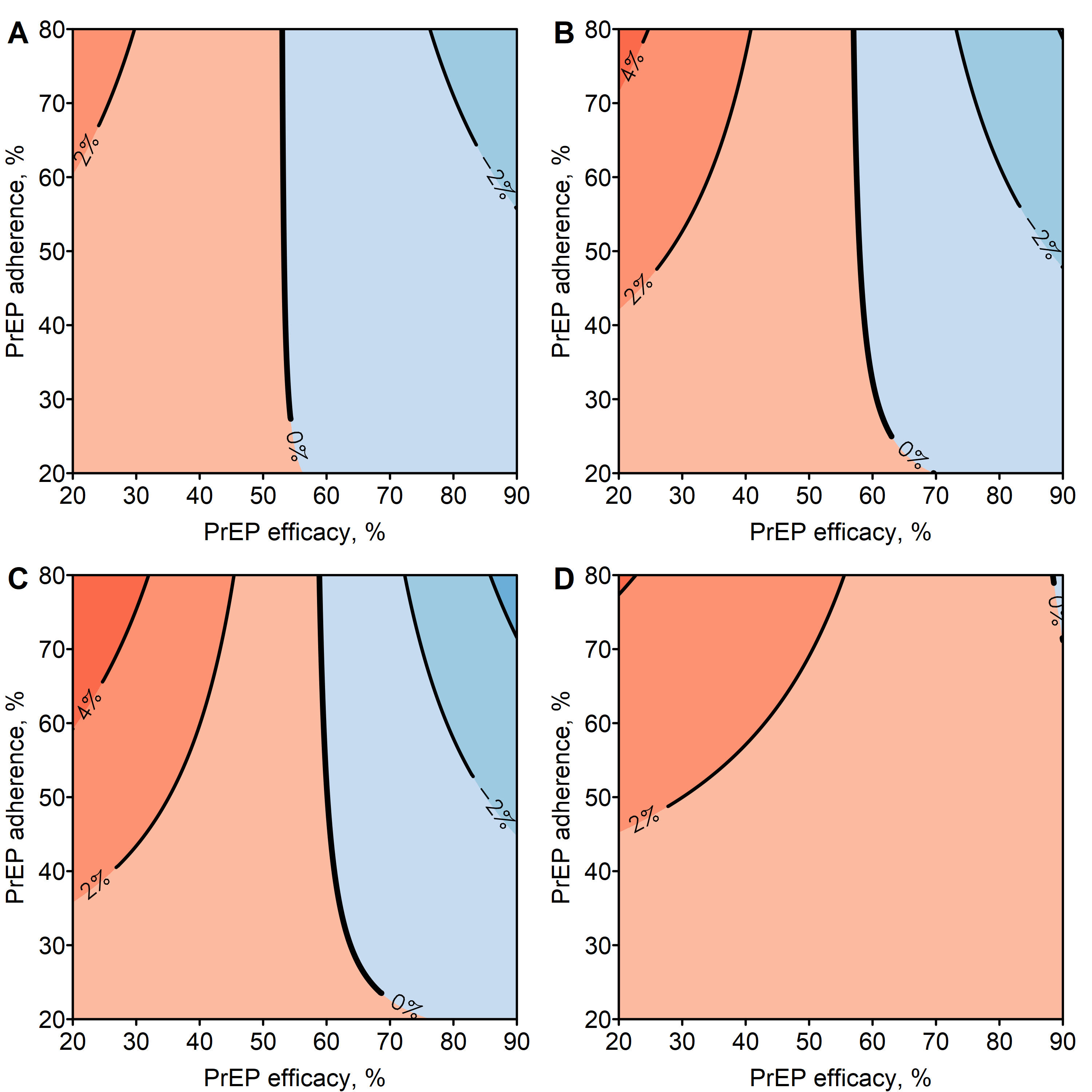


Supplementary Figure 3. Changes in drug resistance after PrEP implementation. Response surfaces show the percentage change in prevalent drug-resistant cases by 2030 after (A) unprioritized, (B) age-prioritized, (C) incidence-based, or (D) FSW PrEP implementation. Response surfaces are calculated as a function of PrEP efficacy and average PrEP adherence in probabilistic sensitivity analysis. Resistance decreases are shown in blue, increases are shown in red. FSW, female sex worker; PrEP, pre-exposure prophylaxis.

**
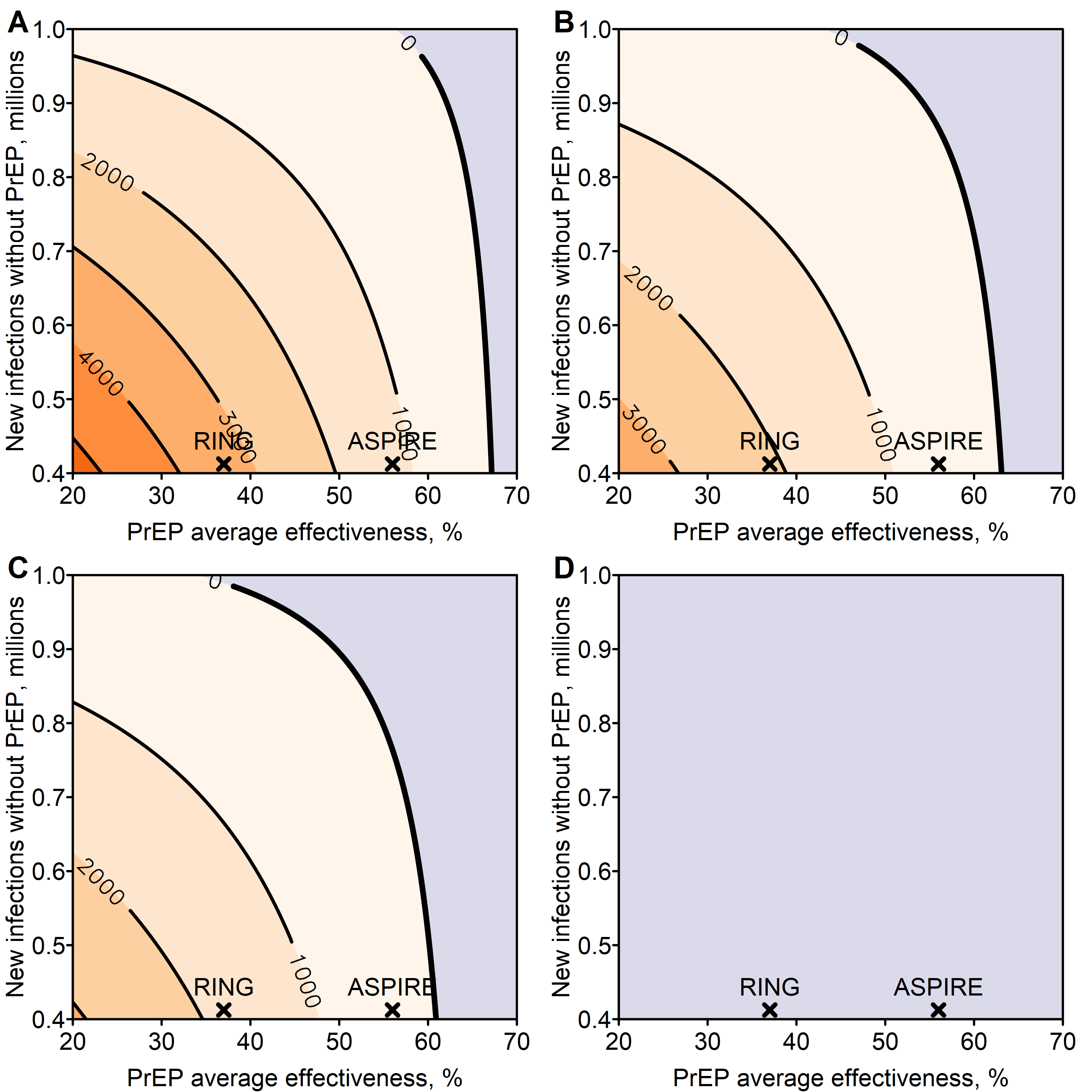
**

Supplementary Figure 4. Lifetime horizon cost-effectiveness of PrEP implementation. Response surfaces show the lifetime horizon cost per life-year gained relative to the reference scenario without PrEP after (A) unprioritized, (B) age-based, (C) incidence-based, or (D) FSW PrEP implementation. Response surfaces are calculated as a function of average PrEP effectiveness (efficacy times average adherence) and cumulative number of infections that occur during 2019-2030 without PrEP implementation. Regions where costs per life-year gained are positive are shown in orange and regions where PrEP is cost-saving are shown in purple. Points marked by ‘x’ denote PrEP effectiveness (RING: 37%, ASPIRE: 56%) and new infections (412,399) values from base-case analyses. FSW, female sex worker; PrEP, pre-exposure prophylaxis.

REFERENCES

1. Glaubius RL, Hood G, Penrose KJ, Parikh UM, Mellors JW, Bendavid E, et al. Cost-effectiveness of injectable preexposure prophylaxis for HIV prevention in South Africa. Clin Infect Dis. 2016;63(4):539-47. doi: 10.1093/cid/ciw321.

2. Glaubius RL, Parikh UM, Hood G, Penrose KJ, Bendavid E, Mellors JW, et al. Deciphering the effects of injectable pre-exposure prophylaxis for combination HIV prevention. Open Forum Infect Dis. 2016;3(3):ofw125. doi: 10.1093/ofid/ofw125.

3. Statistics South Africa. Mid-year population estimates 2013. Pretoria: Statistics South Africa; 2013.

4. Scorgie F, Chersich M, Ntaganira I, Gerbase A, Lule F, Lo Y-R. Socio-demographic characteristics and behavioral risk factors of female sex workers in sub-Saharan Africa: a systematic review. AIDS Behav. 2012;16(4):920-33. doi: 10.1007/s10461-011-9985-z.

5. McGrath N, Eaton JW, Bärnighausen TW, Tanser F, Newell M-L. Sexual behaviour in a rural high HIV prevalence South African community: time trends in the antiretroviral treatment era. AIDS. 2013;27(15):2461-70. doi: 10.1097/01.aids.0000432473.69250.19.

6. Johnson LF, Hallett TB, Rehle TM, Dorrington RE. The effect of changes in condom usage and antiretroviral treatment coverage on human immunodeficiency virus incidence in South Africa: a model-based analysis. J R Soc Interface. 2012;9(72):1544-54. doi: 10.1098/rsif.2011.0826.

7. Shisana O, Rehle T, Simbayi L, Zuma K, Jooste S, Zungu N, et al. South African National HIV Prevalence, Incidence and Behaviour Survey, 2012. Cape Town: HSRC Press; 2014.

8. Gray RH, Wawer MJ, Brookmeyer R, Sewankambo NK, Serwadda D, Wabwire-Mangen F, et al. Probability of HIV-1 transmission per coital act in monogamous, heterosexual, HIV-1-discordant couples in Rakai, Uganda. Lancet. 2001;357(9263):1149-53. doi: 10.1016/S0140-6736(00)04331-2.

9. Hughes JP, Baeten JM, Lingappa JR, Magaret AS, Wald A, de Bruyn G, et al. Determinants of per-coital-act HIV-1 infectivity among African HIV-1-serodiscordant couples. J Infect Dis. 2012;205(3):358-65. doi: 10.1093/infdis/jir747.

10. McClelland RS, Hassan WM, Lavreys L, Richardson BA, Mandaliya K, Ndinya-Achola JO, et al. HIV-1 acquisition and disease progression are associated with decreased high-risk sexual behaviour among Kenyan female sex workers. AIDS. 2006;20(15):1969-73. doi: 10.1097/01.aids.0000247119.12327.e6.

11. McClelland RS, Graham SM, Richardson BA, Peshu N, Masese LN, Wanje GH, et al. Treatment with antiretroviral therapy is not associated with increased sexual risk behavior in Kenyan female sex workers. AIDS. 2010;24(6):891-7. doi: 10.1097/QAD.0b013e32833616c7.

12. McGrath N, Richter L, Newell M-L. Sexual risk after HIV diagnosis: a comparison of pre-ART individuals with CD4>500 cells/microl and ART-eligible individuals in a HIV treatment and care programme in rural KwaZulu-Natal, South Africa. J Int AIDS Soc. 2013;16:18048. doi: 10.7448/IAS.16.1.18048.

13. Deeks SG, Hoh R, Neilands TB, Liegler T, Aweeka F, Petropoulos CJ, et al. Interruption of treatment with individual therapeutic drug classes in adults with multidrug-resistant HIV-1 infection. J Infect Dis. 2005;192(9):1537-44. doi: 10.1086/496892.

14. Paquet AC, Baxter J, Weidler J, Lie Y, Lawrence J, Kim R, et al. Differences in reversion of resistance mutations to wild-type under structured treatment interruption and related increase in replication capacity. PLoS One. 2011;6(1):e14638. doi: 10.1371/journal.pone.0014638.

15. Castro H, Pillay D, Cane P, Asboe D, Cambiano V, Phillips A, et al. Persistence of HIV-1 transmitted drug resistance mutations. J Infect Dis. 2013;208(9):1459-63. doi: 10.1093/infdis/jit345.

16. Jain V, Sucupira MC, Bacchetti P, Hartogensis W, Diaz RS, Kallas EG, et al. Differential persistence of transmitted HIV-1 drug resistance mutation classes. J Infect Dis. 2011;203(8):1174-81. doi: 10.1093/infdis/jiq167.

17. Pingen M, Nijhuis M, de Bruijn JA, Boucher CAB, Wensing AMJ. Evolutionary pathways of transmitted drug-resistant HIV-1. J Antimicrob Chemother. 2011;66(7):1467-80. doi: 10.1093/jac/dkr157.

18. Palmer S, Boltz V, Maldarelli F, Kearney M, Halvas EK, Rock D, et al. Selection and persistence of non-nucleoside reverse transcriptase inhibitor-resistant HIV-1 in patients starting and stopping non-nucleoside therapy. AIDS. 2006;20(5):701-10. doi: 10.1097/01.aids.0000216370.69066.7f.

19. Cong M-e, Youngpairoj AS, Aung W, Sharma S, Mitchell J, Dobard C, et al. Generation and mucosal transmissibility of emtricitabine- and tenofovir-resistant SHIV162P3 mutants in macaques. Virology. 2011;412(2):435-40. doi: 10.1016/j.virol.2011.01.038.

20. Leigh Brown AJ, Frost SDW, Mathews WC, Dawson K, Hellmann NS, Daar ES, et al. Transmission fitness of drug-resistant human immunodeficiency virus and the prevalence of resistance in the antiretroviral-treated population. J Infect Dis. 2003;187(4):683-6. doi: 10.1086/367989.

21. Deeks SG, Wrin T, Liegler T, Hoh R, Hayden M, Barbour JD, et al. Virologic and immunologic consequences of discontinuing combination antiretroviral-drug therapy in HIV-infected patients with detectable viremia. N Engl J Med. 2001;344(7):472-80. doi: 10.1056/NEJM200102153440702.

22. Cong M-e, Heneine W, García-Lerma JG. The fitness cost of mutations associated with human immunodeficiency virus type 1 drug resistance is modulated by mutational interactions. J Virol. 2007;81(6):3037-41. doi: 10.1128/JVI.02712-06.

23. Keele BF, Giorgi EE, Salazar-Gonzalez JF, Decker JM, Pham KT, Salazar MG, et al. Identification and characterization of transmitted and early founder virus envelopes in primary HIV-1 infection. Proc Natl Acad Sci U S A. 2008;105(21):7552-7. doi: 10.1073/pnas.0802203105.

24. Abrahams M-R, Anderson JA, Giorgi EE, Seoighe C, Mlisana K, Ping L-H, et al. Quantitating the multiplicity of infection with human immunodeficiency virus type 1 subtype C reveals a non-poisson distribution of transmitted variants. J Virol. 2009;83(8):3556-67. doi: 10.1128/jvi.02132-08.

25. Haaland RE, Hawkins PA, Salazar-Gonzalez JF, Johnson A, Tichacek A, Karita E, et al. Inflammatory genital infections mitigate a severe genetic bottleneck in heterosexual transmission of subtype A and C HIV-1. PLoS Pathog. 2009;5(1):e1000274. doi: 10.1371/journal.ppat.1000274.

26. Kemal KS, Burger H, Mayers D, Anastos K, Foley B, Kitchen C, et al. HIV-1 drug resistance in variants from the female genital tract and plasma. J Infect Dis. 2007;195(4):535-45. doi: 10.1086/510855.

27. Kantor R, Bettendorf D, Bosch RJ, Mann M, Katzenstein D, Cu-Uvin S, et al. HIV-1 RNA levels and antiretroviral drug resistance in blood and non-blood compartments from HIV-1-infected men and women enrolled in AIDS clinical trials group study A5077. PLoS One. 2014;9(4):e93537. doi: 10.1371/journal.pone.0093537.

28. Bull ME, Heath LM, McKernan-Mullin JL, Kraft KM, Acevedo L, Hitti JE, et al. Human immunodeficiency viruses appear compartmentalized to the female genital tract in cross-sectional analyses but genital lineages do not persist over time. J Infect Dis. 2013;207(8):1206-15. doi: 10.1093/infdis/jit016.

29. Mellors JW, Munoz A, Giorgi JV, Margolick JB, Tassoni CJ, Gupta P, et al. Plasma viral load and CD4+ lymphocytes as prognostic markers of HIV-1 infection. Ann Intern Med. 1997;126(12):946-54. doi: 10.1059/0003-4819-126-12-199706150-00003.

30. Modjarrad K, Chamot E, Vermund SH. Impact of small reductions in plasma HIV RNA levels on the risk of heterosexual transmission and disease progression. AIDS. 2008;22(16):2179-85. doi: 10.1097/QAD.0b013e328312c756.

31. Hamers RL, Schuurman R, Sigaloff KCE, Wallis CL, Kityo C, Siwale M, et al. Effect of pretreatment HIV-1 drug resistance on immunological, virological, and drug-resistance outcomes of first-line antiretroviral treatment in sub-Saharan Africa: a multicentre cohort study. Lancet Infect Dis. 2012;12(4):307-17. doi: 10.1016/S1473-3099(11)70255-9.

32. Baeten JM, Kahle E, Lingappa JR, Coombs RW, Delany-Moretlwe S, Nakku-Joloba E, et al. Genital HIV-1 RNA predicts risk of heterosexual HIV-1 transmission. Sci Transl Med. 2011;3(77):77ra29. doi: 10.1126/scitranslmed.3001888.

33. Penrose KJ, Wallis CL, Brumme CJ, Hamanishi KA, Gordon KC, Viana RV, et al. Frequent cross-resistance to dapivirine in HIV-1 subtype C-infected individuals on failing first-line antiretroviral therapy in South Africa. Antimicrob Agents Chemother. 2017;61(2):e01805-e16. doi: 10.1128/AAC.01805-16.

34. Baeten JM, Palanee-Phillips T, Brown ER, Schwartz K, Soto-Torres L, Govender V, et al. Use of a vaginal ring containing dapivirine for HIV-1 prevention in women. N Engl J Med. 2016;375(22):2121-32. doi: 10.1056/NEJMoa1506110.

35. Nel A, van Niekerk N, Kapiga S, Bekker L-G, Gama C, Gill K, et al. Safety and efficacy of a dapivirine vaginal ring for HIV prevention in women. N Engl J Med. 2016;375(22):2133-43. doi: 10.1056/NEJMoa1602046.

36. Brown E, Palanee-Phillips T, Marzinke M, Hendrix C, Dezutti C, Soto-Torres L, et al. Residual dapivirine ring levels indicate higher adherence to vaginal ring is associated with HIV-1 protection. AIDS 2016; Durban, South Africa2016.

37. Nel AM, Haazen W, Nuttall JP, Romano JW, Mesquita PMM, Herold BC, et al. Pharmacokinetics and safety assessment of anti-HIV dapivirine vaginal microbicide rings with multiple dosing. J AIDS Clin Res. 2014;5:355. doi: 10.4172/2155-6113.1000355.

38. Dobard C, Sharma S, Parikh UM, West R, Taylor A, Martin A, et al. Postexposure protection of macaques from vaginal SHIV infection by topical integrase inhibitors. Sci Transl Med. 2014;6(227):227ra35. doi: 10.1126/scitranslmed.3007701.

39. South Africa National Department of Health. National Consolidated Guidelines for the Prevention of Mother-to-Child Transmission of HIV (PMTCT) and the Management of HIV in Children, Adolescents and Adults. Pretoria: DoH; 2014.

40. Joint United Nations Programme on HIV/AIDS. Fast-Track: ending the AIDS epidemic by 2030. Geneva: Joint United Nations Programme on HIV/AIDS; 2014.

41. South African National AIDS Council (SANAC). South Africa's National Strategic Plan for HIV, TB and STIs 2017-2022. 2017.

42. Zaidi J, Grapsa E, Tanser F, Newell M-L, Bärnighausen T. Dramatic increase in HIV prevalence after scale-up of antiretroviral treatment. AIDS. 2013;27(14):2301-5. doi: 10.1097/QAD.0b013e328362e832.

43. Cohen MS, Chen YQ, McCauley M, Gamble T, Hosseinipour MC, Kumarasamy N, et al. Prevention of HIV-1 infection with early antiretroviral therapy. N Engl J Med. 2011;365(6):493-505. doi: 10.1056/NEJMoa1105243.

44. Clouse K, Pettifor AE, Maskew M, Bassett J, Van Rie A, Gay CL, et al. Initiating antiretroviral therapy when presenting with higher CD4 cell counts results in reduced loss to follow-up in a resource-limited setting. AIDS. 2013;27(4):645-50. doi: 10.1097/QAD.0b013e32835c12f9.

45. Johnson LF, Mossong J, Dorrington RE, Schomaker M, Hoffmann CJ, Keiser O, et al. Life expectancies of South African adults starting antiretroviral treatment: collaborative analysis of cohort studies. PLoS Med. 2013;10(4):e1001418. doi: 10.1371/journal.pmed.1001418.

46. When To Start Consortium, Sterne JAC, May M, Costagliola D, de Wolf F, Phillips AN, et al. Timing of initiation of antiretroviral therapy in AIDS-free HIV-1-infected patients: a collaborative analysis of 18 HIV cohort studies. Lancet. 2009;373(9672):1352-63. doi: 10.1016/S0140-6736(09)60612-7.

47. Hosseinipour MC, Gupta RK, Van Zyl G, Eron JJ, Nachega JB. Emergence of HIV drug resistance during first- and second-line antiretroviral therapy in resource-limited settings. J Infect Dis. 2013;207(Suppl 2):S49-S56. doi: 10.1093/infdis/jit107.

48. South African National AIDS Council (SANAC). National Strategic Plan on HIV, STIs and TB, 2012-2016. Pretoria: SANAC; 2012.

49. KwaZulu-Natal Department of Health. KwaZulu-Natal Department of Health Annual Report 2014/15. Pietermaritzburg, South Africa: KwaZulu-Natal Department of Health; 2015.

50. Auvert B, Taljaard D, Lagarde E, Sobngwi-Tambekou J, Sitta R, Puren A. Randomized, controlled intervention trial of male circumcision for reduction of HIV infection risk: the ANRS 1265 Trial. PLoS Med. 2005;2(11):e298. doi: 10.1371/journal.pmed.0020298.

51. Bailey RC, Moses S, Parker CB, Agot K, Maclean I, Krieger JN, et al. Male circumcision for HIV prevention in young men in Kisumu, Kenya: a randomised controlled trial. Lancet. 2007;369(9562):643-56. doi: 10.1016/S0140-6736(07)60312-2.

52. Gray RH, Kigozi G, Serwadda D, Makumbi F, Watya S, Nalugoda F, et al. Male circumcision for HIV prevention in men in Rakai, Uganda: a randomised trial. Lancet. 2007;369(9562):657-66. doi: 10.1016/S0140-6736(07)60313-4.

53. Wawer MJ, Makumbi F, Kigozi G, Serwadda D, Watya S, Nalugoda F, et al. Circumcision in HIV-infected men and its effect on HIV transmission to female partners in Rakai, Uganda: a randomised controlled trial. Lancet. 2009;374(9685):229-37. doi: 10.1016/S0140-6736(09)60998-3.

54. Levison JH, Orrell C, Gallien S, Kuritzkes DR, Fu N, Losina E, et al. Virologic failure of protease inhibitor-based second-line antiretroviral therapy without resistance in a large HIV treatment program in South Africa. PLoS One. 2012;7(3):e32144. doi: 10.1371/journal.pone.0032144.

55. Garnett GP, Anderson RM. Balancing sexual partnerships in an age and activity stratified model of HIV transmission in heterosexual populations. IMA J Math Appl Med Biol. 1994;11(3):161-92. doi: 10.1093/imammb/11.3.161.

56. Garnett GP, Anderson RM. Factors controlling the spread of HIV in heterosexual communities in developing countries: patterns of mixing between different age and sexual activity classes. Philos Trans R Soc Lond B Biol Sci. 1993;342(1300):137-59. doi: 10.1098/rstb.1993.0143.

57. Cremin I, Alsallaq R, Dybul M, Piot P, Garnett GP, Hallett TB. The new role of antiretrovirals in combination HIV prevention: a mathematical modelling analysis. AIDS. 2013;27(3):447-58. doi: 10.1097/QAD.0b013e32835ca2dd.

58. World Health Organization. Antiretroviral Therapy for HIV Infection in Adults and Adolescents: Recommendations for a Public Health Approach: 2010 Revision. Geneva: World Health Organization; 2010.

59. Venter F. Second line and future regimens in sub-Saharan Africa: what's coming? XXV International HIV Drug Resistance Workshop; Boston, MA2016.

60. Mossong J, Grapsa E, Tanser F, Bärnighausen T, Newell M-L. Modelling HIV incidence and survival from age-specific seroprevalence after antiretroviral treatment scale-up in rural South Africa. AIDS. 2013;27(15):2471-9. doi: 10.1097/01.aids.0000432475.14992.da.

61. Tanser F, Bärnighausen T, Grapsa E, Zaidi J, Newell M-L. High coverage of ART associated with decline in risk of HIV acquisition in rural KwaZulu-Natal, South Africa. Science. 2013;339(6122):966-71. doi: 10.1126/science.1228160.

62. South African Centre for Epidemiological Modelling and Analysis. The Modes of Transmission of HIV in South Africa. Stellenbosch: South African Centre for Epidemiological Modelling and Analysis; 2009.

63. Raftery AE, Bao L. Estimating and projecting trends in HIV/AIDS generalized epidemics using incremental mixture importance sampling. Biometrics. 2010;66(4):1162-73. doi: 10.1111/j.1541-0420.2010.01399.x.

64. Alkema L, Raftery AE, Clark SJ. Probabilistic projections of HIV prevalence using Bayesian melding. Ann Appl Stat. 2007;1(1):229-48. doi: 10.1214/07-aoas111.

65. Johnson L, Dorrington R, Bradshaw D, Pillay-Van Wyk V, Rehle T. Sexual behaviour patterns in South Africa and their association with the spread of HIV: insights from a mathematical model. Demogr Res. 2009;21(11):289-340. doi: 10.4054/DemRes.2009.21.11.

66. Ramjee G, Weber AE, Morar NS. Recording sexual behavior: comparison of recall questionnaires with a coital diary. Sex Transm Dis. 1999;26(7):374-80. doi: 10.1097/00007435-199908000-00002.

67. Caraël M, Slaymaker E, Lyerla R, Sarkar S. Clients of sex workers in different regions of the world: hard to count. Sex Transm Infect. 2006;82(Suppl 3):iii26-iii33. doi: 10.1136/sti.2006.021196.

68. Vandepitte J, Lyerla R, Dallabetta G, Crabbé F, Alary M, Buvé A. Estimates of the number of female sex workers in different regions of the world. Sex Transm Infect. 2006;82(Suppl 3):iii18-iii25. doi: 10.1136/sti.2006.020081.

69. Brown MS. Coitus, the proximate determinant of conception: inter-country variance in sub-Saharan Africa. J Biosoc Sci. 2000;32(2):145-59. doi: 10.1017/S0021932000001450.

70. Myer L, Mathews C, Little F. Condom use and sexual behaviors among individuals procuring free male condoms in South Africa: a prospective study. Sex Transm Dis. 2002;29(4):239-41. doi: 10.1097/00007435-200204000-00009.

71. Weller SC, Davis-Beaty K. Condom effectiveness in reducing heterosexual HIV transmission. Cochrane Database Syst Rev. 2002;(1):CD003255. doi: 10.1002/14651858.CD003255.

72. Boily M-C, Baggaley RF, Wang L, Mâsse B, White RG, Hayes RJ, et al. Heterosexual risk of HIV-1 infection per sexual act: systematic review and meta-analysis of observational studies. Lancet Infect Dis. 2009;9(2):118-29. doi: 10.1016/S1473-3099(09)70021-0.

73. Quinn TC, Wawer MJ, Sewankambo N, Serwadda D, Li C, Wabwire-Mangen F, et al. Viral load and heterosexual transmission of human immunodeficiency virus type 1. N Engl J Med. 2000;342(13):921-9. doi: 10.1056/NEJM200003303421303.

74. Wawer MJ, Gray RH, Sewankambo NK, Serwadda D, Li X, Laeyendecker O, et al. Rates of HIV-1 transmission per coital act, by stage of HIV-1 infection, in Rakai, Uganda. J Infect Dis. 2005;191(9):1403-9. doi: 10.1086/429411.

75. Pilcher CD, Price MA, Hoffman IF, Galvin S, Martinson FEA, Kazembe PN, et al. Frequent detection of acute primary HIV infection in men in Malawi. AIDS. 2004;18(3):517-24. doi: 10.1097/01.aids.0000111398.02002.de.

76. Novitsky V, Wang R, Bussmann H, Lockman S, Baum M, Shapiro R, et al. HIV-1 subtype C-infected individuals maintaining high viral load as potential targets for the "test-and-treat" approach to reduce HIV transmission. PLoS One. 2010;5(4):e10148. doi: 10.1371/journal.pone.0010148.

77. Cohen MS, Gay CL, Busch MP, Hecht FM. The detection of acute HIV infection. J Infect Dis. 2010;202(Suppl 2):S270-S7. doi: 10.1086/655651.

78. Fiebig EW, Wright DJ, Rawal BD, Garrett PE, Schumacher RT, Peddada L, et al. Dynamics of HIV viremia and antibody seroconversion in plasma donors: implications for diagnosis and staging of primary HIV infection. AIDS. 2003;17(13):1871-9. doi: 10.1097/01.aids.0000076308.76477.b8.

79. Lodi S, Phillips A, Touloumi G, Geskus R, Meyer L, Thiébaut R, et al. Time from human immunodeficiency virus seroconversion to reaching CD4+ cell count thresholds <200, <350, and <500 cells/mm^3^: assessment of need following changes in treatment guidelines. Clin Infect Dis. 2011;53(8):817-25. doi: 10.1093/cid/cir494.

80. Van der Paal L, Shafer LA, Todd J, Mayanja BN, Whitworth JAG, Grosskurth H. HIV-1 disease progression and mortality before the introduction of highly active antiretroviral therapy in rural Uganda. AIDS. 2007;21(Suppl 6):S21-S9. doi: 10.1097/01.aids.0000299407.52399.05.

81. U.S. Census Bureau. International data base: South Africa, 1985 Washington D.C.: U.S. Census Bureau; 2014 [cited 2014 Mar 27, 2014]. Available from: <http://www.census.gov/population/international/data/idb/informationGateway.php>.

82. WHO. Global Health Observatory Data Repository World Health Organization, Geneva2013 [cited 2013 2013-11-15]. South Africa life tables]. Available from: <http://apps.who.int/gho/data/node.main>.

83. Kripke K, Chen P-A, Vazzano A, Thambinayagam A, Pillay Y, Loykissoonlal D, et al. Cost and impact of voluntary medical male circumcision in South Africa: focusing the program on specific age groups and provinces. PLoS One. 2016;11(7):e0157071. doi: 10.1371/journal.pone.0157071.

84. Mehta SD, Moses S, Agot K, Odoyo-June E, Li H, Maclean I, et al. The long-term efficacy of medical male circumcision against HIV acquisition. AIDS. 2013;27(18):2709-899. doi: 10.1097/01.aids.0000432444.30308.2d.

85. Grimsrud AT, Cornell M, Egger M, Boulle A, Myer L. Impact of definitions of loss to follow-up (LTFU) in antiretroviral therapy program evaluation: variation in the definition can have an appreciable impact on estimated proportions of LTFU. J Clin Epidemiol. 2013;66(9):1006-13. doi: 10.1016/j.jclinepi.2013.03.013.

86. Johnston V, Cohen K, Wiesner L, Morris L, Ledwaba J, Fielding KL, et al. Viral suppression following switch to second-line antiretroviral therapy: associations with nucleoside reverse transcriptase inhibitor resistance and subtherapeutic drug concentrations prior to switch. J Infect Dis. 2014;209(5):711-20. doi: 10.1093/infdis/jit411.

87. Fox MP, Ive P, Long L, Maskew M, Sanne I. High rates of survival, immune reconstitution, and virologic suppression on second-line antiretroviral therapy in South Africa. J Acquir Immune Defic Syndr. 2010;53(4):500-6. doi: 10.1097/QAI.0b013e3181bcdac1.

88. Barth RE, van der Loeff MFS, Schuurman R, Hoepelman AIM, Wensing AMJ. Virological follow-up of adult patients in antiretroviral treatment programmes in sub-Saharan Africa: a systematic review. Lancet Infect Dis. 2010;10(3):155-66. doi: 10.1016/S1473-3099(09)70328-7.

89. Li JZ, Paredes R, Ribaudo HJ, Svarovskaia ES, Metzner KJ, Kozal MJ, et al. Low-frequency HIV-1 drug resistance mutations and risk of NNRTI-based antiretroviral treatment failure: a systematic review and pooled analysis. JAMA. 2011;305(13):1327-35. doi: 10.1001/jama.2011.375.

90. Joint United Nations Programme on HIV/AIDS. Global AIDS Update 2017 - Ending AIDS: Progress Towards the 90-90-90 Targets. Geneva: Joint United Nations Programme on HIV/AIDS; 2017.

91. World Health Organization. WHO HIV Drug Resistance Report 2012. Geneva: World Health Organization; 2012.

92. Aghokeng AF, Monleau M, Eymard-Duvernay S, Dagnra A, Kania D, Ngo-Giang-Huong N, et al. Extraordinary heterogeneity of virological outcomes in patients receiving highly antiretroviral therapy and monitored with the World Health Organization public health approach in sub-Saharan Africa and southeast Asia. Clin Infect Dis. 2014;58(1):99-109. doi: 10.1093/cid/cit627.

93. Hamers RL, Sigaloff KCE, Wensing AM, Wallis CL, Kityo C, Siwale M, et al. Patterns of HIV-1 drug resistance after first-line antiretroviral therapy (ART) failure in 6 sub-Saharan African countries: implications for second-line ART strategies. Clin Infect Dis. 2012;54(11):1660-9. doi: 10.1093/cid/cis254.

94. Wallis CL, Mellors JW, Venter WDF, Sanne I, Stevens W. Protease inhibitor resistance is uncommon in HIV-1 subtype C infected patients on failing second-line lopinavir/r-containing antiretroviral therapy in South Africa. AIDS Res Treat. 2011;2011:769627. doi: 10.1155/2011/769627.

95. Boender TS, Hamers RL, Ondoa P, Wellington M, Siwale M, Kityo CM, et al. Protease inhibitor resistance at 2nd-line HIV treatment failure in sub-Saharan Africa. 23rd Conference on Retroviruses and Opportunistic Infections (CROI 2016); Feb 22-25; Boston, Massachusetts2016.

96. Baeten J, Palanee-Phillips T, Mgodi N, Mayo A, Nel A, Rosenberg Z, et al. High uptake and reduced HIV-1 incidence in an open-label trial of the dapvirine ring. 25th Conference on Retroviruses and Opportunistic Infections (CROI 2018); Boston, Massachusetts2018.

97. Schader SM, Oliveira M, Ibanescu R-I, Moisi D, Colby-Germinario SP, Wainberg MA. In vitro resistance profile of the candidate HIV-1 microbicide drug dapivirine. Antimicrob Agents Chemother. 2012;56(2):751-6. doi: 10.1128/AAC.05821-11.

98. Hsu M, Keele BF, Aravantinou M, Krawczyk N, Seidor S, Abraham CJ, et al. Exposure to MIV-150 from a high-dose intravaginal ring results in limited emergence of drug resistance mutations in SHIV-RT infected rhesus macaques. PLoS One. 2014;9(2):e89300. doi: 10.1371/journal.pone.0089300.

99. El-Khatib Z, Ekstrom AM, Ledwaba J, Mohapi L, Laher F, Karstaedt A, et al. Viremia and drug resistance among HIV-1 patients on antiretroviral treatment: a cross-sectional study in Soweto, South Africa. AIDS. 2010;24(11):1679-87. doi: 10.1097/QAD.0b013e32833a097b.

100. Hosseinipour MC, van Oosterhout JJG, Weigel R, Phiri S, Kamwendo D, Parkin N, et al. The public health approach to identify antiretroviral therapy failure: high-level nucleoside reverse transcriptase inhibitor resistance among Malawians failing first-line antiretroviral therapy. AIDS. 2009;23(9):1127-34. doi: 10.1097/QAD.0b013e32832ac34e.

101. Armstrong KL, Lee T-H, Essex M. Replicative fitness costs of nonnucleoside reverse transcriptase inhibitor drug resistance mutations on HIV subtype C. Antimicrob Agents Chemother. 2011;55(5):2146-53. doi: 10.1128/AAC.01505-10.

102. Hsieh S-M, Pan S-C, Chang S-Y, Hung C-C, Sheng W-H, Chen M-Y, et al. Differential impact of resistance-associated mutations to protease inhibitors and nonnucleoside reverse transcriptase inhibitors on HIV-1 replication capacity. AIDS Res Hum Retroviruses. 2013;29(8):1117-22. doi: 10.1089/AID.2013.0038.

103. Xu H-T, Oliveira M, Quashie PK, McCallum M, Han Y, Quan Y, et al. Subunit-selective mutational analysis and tissue culture evaluations of the interactions of the E138K and M184I mutations in HIV-1 reverse transcriptase. J Virol. 2012;86(16):8422-31. doi: 10.1128/JVI.00271-12.

104. World Health Organization. Making choices in health: WHO guide to cost-effectiveness analysis. World Health Organization, Geneva: 2003.

105. Meyer-Rath G, Johnson LF, Pillay Y, Blecher M, Brennan AT, Long L, et al. Changing the South African national antiretroviral therapy guidelines: The role of cost modelling. PLoS One. 2017;12(10):e0186557. doi: 10.1371/journal.pone.0186557.

106. Tagar E, Sundaram M, Condliffe K, Matatiyo B, Chimbwandira F, Chilima B, et al. Multi-country analysis of treatment costs for HIV/AIDS (MATCH): facility-level ART unit cost analysis in Ethiopia, Malawi, Rwanda, South Africa and Zambia. PLoS One. 2014;9(11):e108304. doi: 10.1371/journal.pone.0108304.

107. World Health Organization. Global Price Reporting Mechanism 2018 [cited 2018 June 30]. Available from: <http://apps.who.int/hiv/amds/price/hdd/>.

108. Smith JA, Sharma M, Levin C, Baeten JM, van Rooyen H, Celum C, et al. Cost-effectiveness of community-based strategies to strengthen the continuum of HIV care in rural South Africa: a health economic modelling analysis. Lancet HIV. 2015;2(4):e159-e68. doi: 10.1016/S2352-3018(15)00016-8.

109. Fatti G, Jackson D, Goga AE, Shaikh N, Eley B, Nachega JB, et al. The effectiveness and cost-effectiveness of community-based support for adolescents receiving antiretroviral treatment: an operational research study in South Africa. J Int AIDS Soc. 2018;21(Suppl 1). doi: 10.1002/jia2.25041.

110. Meyer-Rath G, Brennan AT, Fox MP, Modisenyane T, Tshabangu N, Mohapi L, et al. Rates and cost of hospitalization before and after initiation of antiretroviral therapy in urban and rural settings in South Africa. J Acquir Immune Defic Syndr. 2013;62(3):322-8. doi: 10.1097/QAI.0b013e31827e8785.

111. Tchuenche M, Palmer E, Haté V, Thambinayagam A, Loykissoonlal D, Stegman P, et al. The cost of voluntary medical male circumcision in South Africa. AIDS 2016; Durban, South Africa2016.

112. FSG. Dapivirine Ring: The Case For Action. 2017.

113. Phillips A, Nakagawa F, Cambiano V, Homan R, Rehle T, Meyer-Rath G, et al. Potential effectiveness and cost effectiveness of condomless sex targeted PrEP in KZN, South Africa: considerations of drug resistance, ART regimen and HIV testing frequency. 22nd International AIDS Conference (AIDS 2018); Amsterdam, Netherlands2018.

114. Ndlovu N, Vilakazi M, Majozi M, Sithole F, Mbatha K, Guthrie T. Trends in national and provincial health and HIV/AIDS budgeting and spending in South Africa. Cape Town: Centre for Economic Governance and AIDS in Africa; 2013.

115. Drummond MF, Sculpher MJ, Torrance GW, O'Brien BJ, Stoddart GL. Methods for the Economic Evaluation of Healthcare Programmes. 3rd ed. New York: Oxford University Press; 2005.
